# An integrated framework for UAV-based precision plant protection in complex terrain: the ACHAGA solution for multi-tea fields

**DOI:** 10.3389/fpls.2024.1440234

**Published:** 2024-09-26

**Authors:** Pengyang Zhang, Yangyang Liu, Hongbin Du

**Affiliations:** ^1^ College of Horticulture and Forestry, Tarim University, Alar, China; ^2^ Xinjiang Production and Construction Corps Key Laboratory of Facility Agriculture, Alar, China; ^3^ College of Engineering, Anhui Agricultural University, Hefei, China

**Keywords:** UAV-based plant protection, multi-tea field plant protection, unmanned aerial vehicle, replenishment -scheduling optimization, scheduling route planning

## Abstract

UAV-based plant protection represents an efficient, energy-saving agricultural technology with significant potential to enhance tea production. However, the complex terrain of hilly and mountainous tea fields, coupled with the limited endurance of UAVs, presents substantial challenges for efficient route planning. This study introduces a novel methodological framework for UAV-based precision plant protection across multiple tea fields, addressing the difficulties in planning the shortest routes and optimal flights for UAVs constrained by their endurance. The framework employs a hyperbolic genetic annealing algorithm (ACHAGA) to optimize UAV plant protection routes with the objectives of minimizing flight distance, reducing the number of turns, and enhancing route stability. The method involves two primary steps: cluster partitioning and sortie allocation for multiple tea fields based on UAV range capabilities, followed by refining the UAV’s flight path using a combination of hyperbolic genetic and simulated annealing algorithms with an adaptive temperature control mechanism. Simulation experiments and UAV route validation tests confirm the effectiveness of ACHAGA. The algorithm consistently identified optimal solutions within an average of 40 iterations, demonstrating robust global search capabilities and stability. It achieved an average reduction of 45.75 iterations and 1811.93 meters in the optimal route, with lower variation coefficients and extreme deviations across repeated simulations. ACHAGA significantly outperforms these algorithms, GA, GA-ACO, AFSA and BSO, which are also heuristic search strategies, in the multi-tea field route scheduling problem, reducing the optimal routes by 4904.82 m, 926.07 m, 3803.96 m and 800.11 m, respectively. Field tests revealed that ACHAGA reduced actual flight routes by 791.9 meters and 359.6 meters compared to manual and brainstorming-based planning methods, respectively. Additionally, the algorithm reduced flight scheduling distance and the number of turns by 11 compared to manual planning. This study provides a theoretical and technical foundation for managing large-scale tea plantations in challenging landscapes and serves as a reference for UAV precision operation planning in complex environments.

## 1 Introduction

In recent years, Unmanned Aerial Vehicles (UAVs) have emerged as a crucial tool in modern agriculture, offering efficient and eco-friendly solutions tailored to specific needs. The tea industry, with its intricate field layouts, diverse topography, and varied cultivation patterns, exemplifies the unique challenges of plant protection that UAV technology can address (Zhang and Zhu, 2023; Paraforos et al., 2022). Historically, agriculture has witnessed a gradual but significant shift towards automation and technological integration. UAVs, once a novelty, are now at the forefront of this evolution, playing a pivotal role in crop monitoring, pest control, and resource management (Bao et al., 2023; Verma et al., 2023). Their ability to navigate difficult terrains and distribute treatments with unprecedented precision has not only enhanced crop yield and quality but also reduced environmental impact.

Despite the clear advantages, scheduling and routing UAVs in the tea industry remain complex tasks. Traditional methods, heavily reliant on human experience and subjective judgment, fall short in addressing the non-linear and dynamic nature of tea field layouts. The existing literature on UAV route optimization in complex terrains highlights several approaches, such as the A* algorithm, Rapidly-exploring Random Trees (RRT), and various meta-heuristic algorithms (Ait Saadi et al., 2022; He et al., 2024). However, these methods often struggle with the high computational demands and adaptability required for real-time operations in uneven and unpredictable environments. Additionally, the limited battery life of UAVs imposes stringent constraints on flight distance and operational time. This necessitates highly efficient and adaptive route optimization to ensure UAVs can complete their tasks within a single charge. The non-linear and dynamic nature of tea field layouts further complicates the planning process, as routes must continuously adapt to the intricate and changing topography to ensure comprehensive coverage and efficient resource use.

This paper explores the UAV multi-tea field resupply and dispatch route planning challenge, encompassing both site selection and route optimization. Extensive research has been conducted globally on UAV dispatch center location planning. For instance, Bian et al. (Bian et al., 2022). introduced a UAV base station positioning approach utilizing the spiral algorithm, which formulates an air-to-ground propagation model reflecting the actual geographic environment to identify optimal UAV base station sites in complex settings. However, this approach primarily targets communication optimization rather than the logistical challenges specific to agricultural operations. Famili et al. (Famili and Stavrou, 2022) acknowledged the constraints of UAV battery life, proposing an optimization framework based on an approximation algorithm to ascertain the minimal number of charging stations needed for sustained flight capability. This study, while addressing the critical issue of battery life, does not fully tackle the complexities of dynamic route planning in varied terrains. Saavedrai et al. (Saavedra et al., 2021) developed an adaptive and comprehensive capacity-constrained localization-routing (CLRP) model for UAV identification in post-disaster relief, effectively pinpointing ideal locations and routes for UAV hubs in preparation for disasters. Although this model is robust for emergency scenarios, it does not account for the continuous and repetitive nature of agricultural UAV operations. Li et al. (Li et al., 2019) enhanced the firefly algorithm for logistics distribution center siting focuses on maximizing benefits based on the performance characteristics and limitations of logistics UAVs. This approach is effective for static distribution centers but lacks the adaptability required for real-time agricultural resupply and dispatch. While these studies above primarily focused on identifying optimal locations for specific purposes such as communication, charging, and rescue, the current paper addresses the distinct challenge of planning dynamic multi-tea field supply and dispatch routes. This entails UAV route optimization for efficient resupply and dispatch across tea fields, ensuring effective operations and comprehensive coverage—a novel and complex aspect of aerial plant protection planning.

The scheduling of UAV route tasks is a significant research area, involving the development of optimal flight schemes for UAVs across various application scenarios. Researchers worldwide have devised numerous route planning algorithms for this domain, engaging in comprehensive discussions centered on diverse optimization goals. For instance, Li et al. (Li et al., 2022c) redefined the challenge of optimal task distribution among multiple UAVs as a combinatorial optimization problem and developed a sequence-independent enumeration algorithm, significantly reducing flight frequency. However, this method does not integrate resupply point planning, which is crucial for large-scale agricultural settings. Xu et al. (Xu et al., 2020) addressed the assignment and sequencing of UAV operational tasks by formulating a bi-objective model, prioritizing non-operational flight distance and total operational time, and introduced an enhanced MOSFLA algorithm. This model is effective for minimizing travel distances, but it does not consider the dynamic nature of resupply in multi-field environments. Sun et al. (Sun et al., 2020) presented a dragonfly-inspired scheduling method for agricultural UAVs, focusing on plant protection and charging operations, which efficiently identifies near-optimal schedules. Despite its efficiency, it lacks a comprehensive approach to resupply point selection. Li et al. (Li et al., 2022b) proposed a novel approach for route planning and task allocation during UAV fly-over operations, optimizing task distribution through a particle swarm algorithm with flight control time as the objective, constrained by UAV battery life and payload capacity. This approach, while innovative, does not address the specific requirements of multi-field agricultural resupply. Fesenko et al. (Fesenko et al., 2020) responded to the nascent state of UAV monitoring technology for nuclear power plants by proposing an algorithm for automated battery exchange at aerial deployment sites, facilitating the development of UAV flight schedules and automatic replacement systems. This algorithm is tailored for fixed installation sites and does not accommodate the fluid resupply needs of agricultural UAVs. While these aforementioned algorithms achieve cost-effective and concise scheduling solutions for their respective problems, they diverge from the multi-tea field route scheduling problem based on supply partitioning addressed in this paper. Primarily, the problem models employed by the aforementioned methods differ from the one in this study. Moreover, previous methods have not considered UAV resupply point planning and selection, focusing instead on route optimization for small-scale operations with predetermined fixed resupply points. In contrast, this study’s multi-tea field scheduling route planning problem also encompasses the UAV multi-tea field resupply challenges including resupply location selection and multi-task allocation planning. This research area has seen limited exploration, thus this study contributes to bridging a notable gap in the field.

Despite its efficiency, it lacks a comprehensive approach to resupply point selection. To overcome these limitations, this paper introduces a novel scheduling and routing methodology for UAVs in multi-tea field operations. By analyzing the operational area’s characteristics and requirements, we have developed a model that not only identifies optimal supply points but also provides a comprehensive route plan for multi-field plant protection. The non-linear and dynamic nature of tea field layouts, combined with the limited battery life of UAVs, poses significant challenges in ensuring efficient and continuous operations. This study redefines the UAV multi-field tea plantation resupply and routing challenge as a multiple traveling salesman problem (mTSP), offering a tailored solution to the unique distribution and routing complexities of tea fields. Through this innovative approach, we aim to facilitate swift, effective, and consistent plant protection operations, setting a new benchmark for precision agriculture in the tea industry.

## 2 Details of optimization techniques

### 2.1 Environmental projection of tea fields

**Figure 1:**
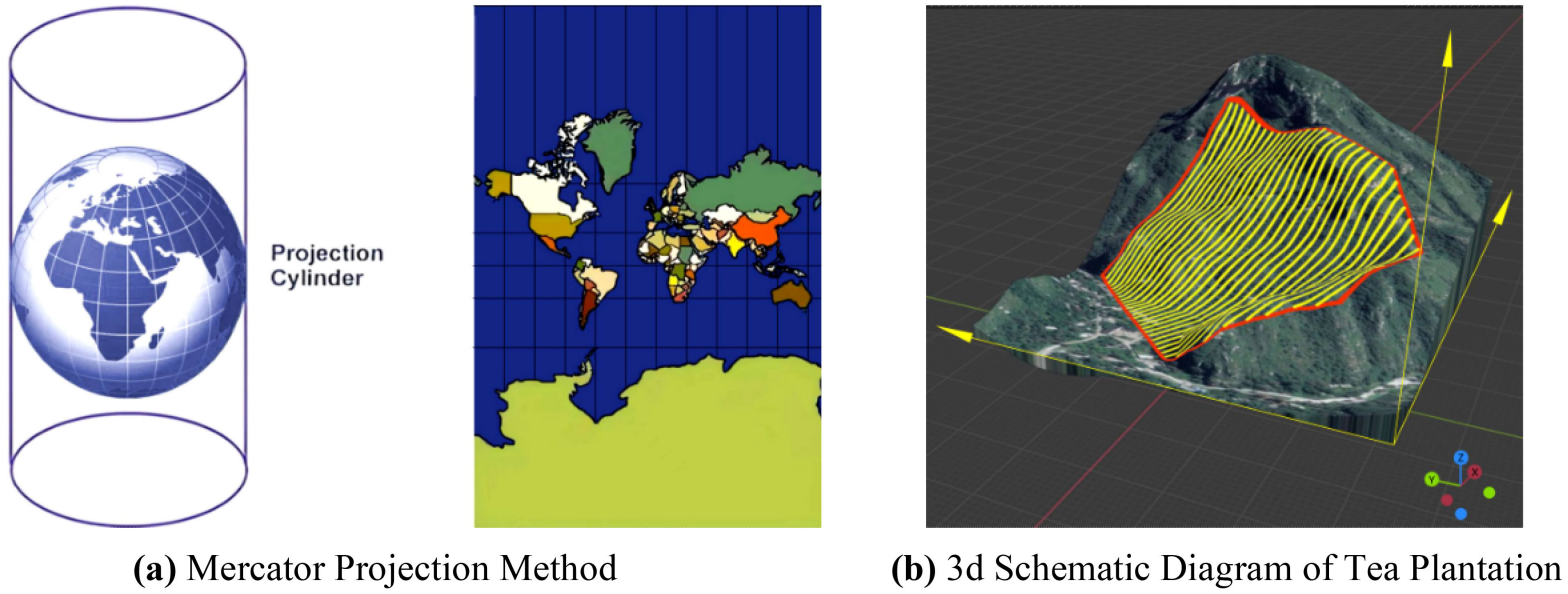
Schematic diagram of projected coordinate system of tea plantation. **(A)** Mercator Projection Method. **(B)** 3d Schematic Diagram of Tea Plantation.

This study aims to facilitate the scheduling and route planning of UAV operations across multiple tea fields, necessitating environmental modeling of the tea plantation to acquire geographic coordinates of individual tea field. As depicted in **Figures 1A, B**, the orthophoto map of the tea plantation is derived using a remote sensing dataset, which processes and translates the geographic coordinates into planar coordinates via the Mercator projection method:

The Mercator projection is a way to represent the curved surface of the Earth on a flat plane. It preserves the right angles between latitude and longitude lines, making it useful for navigation. However, it can cause distortions, especially near the poles. In this method, points on the Earth’s surface with coordinates (*0*, *λ*_0_) are transformed to planar coordinates (*x*, *y*), where *x* represents horizontal distances and *y* is determined by the following Equations 1 and 2:


(1) $$x=R(\lambda -\lambda_{0})\cos(\varphi_{0})$$

(2) $$y=R\ln[\tan(\frac{\pi }{4}+\frac{\varphi }{2\sec(\varphi_{0})}]$$

Given known planar coordinates, conversion back to geographic coordinates is achievable through Equations 3 and 4.


(3) $$\varphi =2\tan^{-1}\left({\text{e}}^{y}\right)-\frac{1}{2}\pi$$

(4) $$\lambda =\text{x}+\lambda_{0}$$

Where *x* is the projected horizontal coordinate; *y* is the projected vertical coordinate; *R* is the Earth’s radius; $\lambda_{0}$ is the central meridian’s longitude; $\varphi_{0}$ is the central meridian’s latitude.

**Figure 2:**
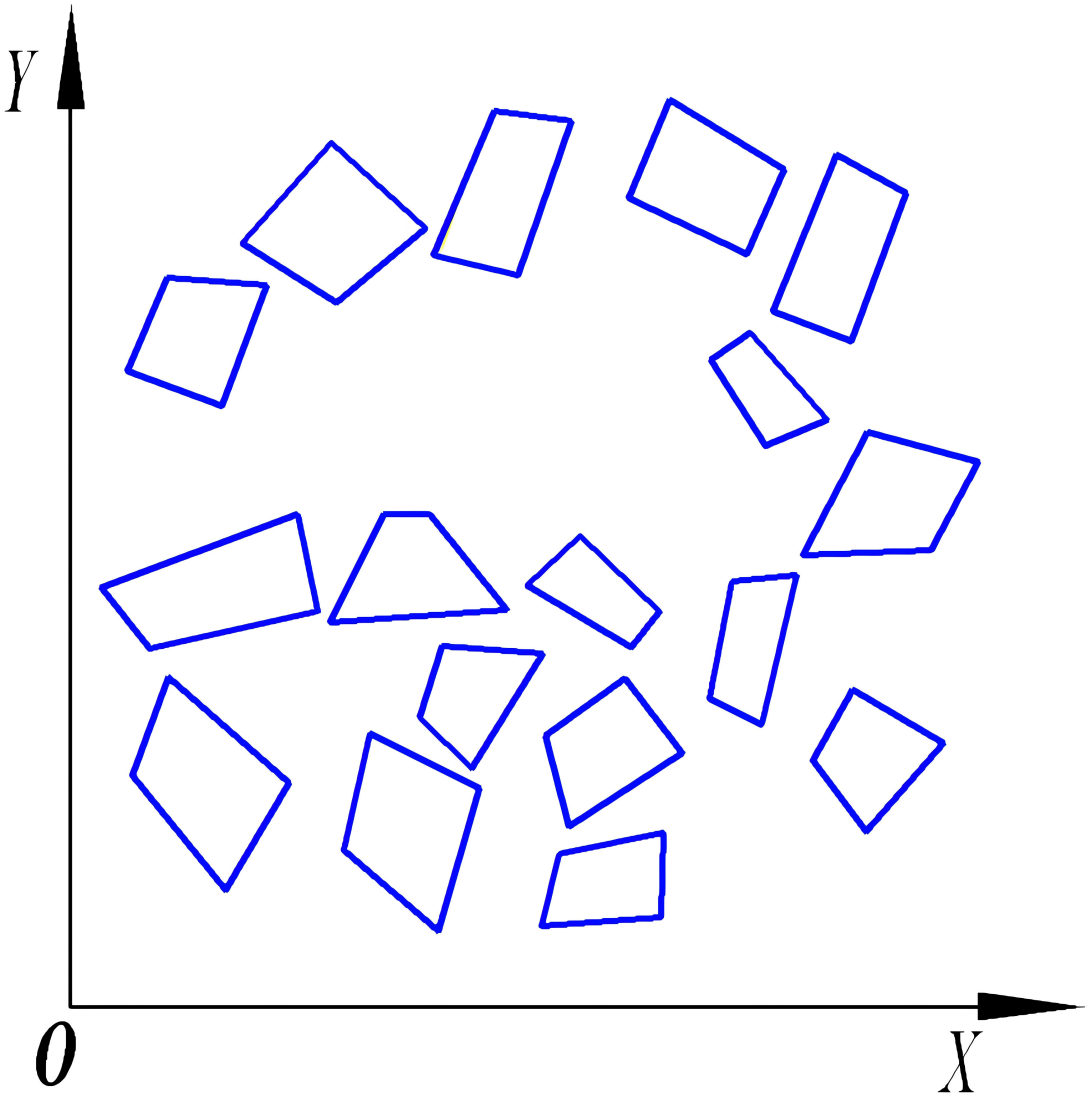
Constructing the coordinate system of multi-tea field operation area.

UAV route planning for plant protection can be categorized into two approaches: single-area planning and multi-field planning. Single-area planning focuses solely on operational width to determine the shortest coverage route, while multi-field planning involves projecting regional boundaries and establishing a planar coordinate system. This study, using a hilly mountainous region as a reference, selects several polygonal fields at random for the operational area. These fields reside within the eastern longitudinal and northern latitudinal zones. Employing the two fields with the minimal latitude and longitude as the reference point, all operational area boundaries fall within the first quadrant, leading to the establishment of a multi-tea field environmental coordinate system, depicted in **Figure 2**.

**Figure 3:**
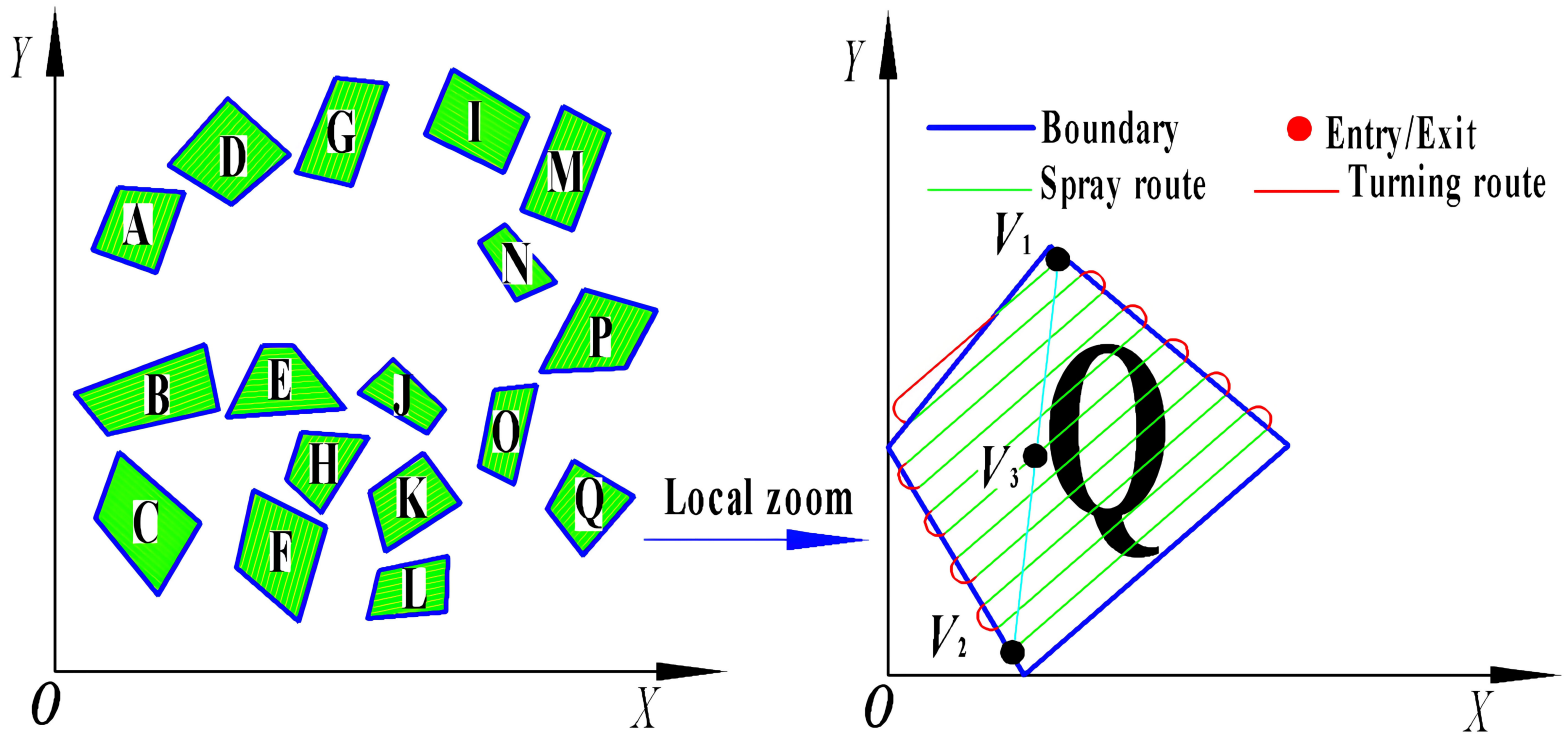
Extract field feature vertices.

Previous research results indicate that by employing a coordinate system—where the boundaries of an operational area define the x-axis origins—we can calculate and compare to ascertain the most efficient full-coverage routes within a single operational area (Liu et al., 2022). These routes minimize the journey length, reduce excess coverage, and ensure that there is exactly one optimal full-coverage route per operational area, characterized by unique starting and ending points. For multi-area scheduling, the voyage encompasses the distance from the endpoint of one field to the starting point of the next, as depicted in **Figure 3**. This paper introduces the midpoint V3, between the start point V1 and the endpoint V2 of a single area, as the defining vertex representing the area. By extracting these characteristic vertices from multiple fields, we generate a discrete point distribution graph, thereby transforming the multi-tea field scheduling problem into a multiple traveling salesman problem model.

This study investigates the UAV planting operation planning in hilly, mountainous tea fields, a variant of the multiple traveling salesman problem with unmanned aerial vehicle (MTSPU) (Zhang et al., 2023; Yan et al., 2024). It accounts for the dynamic distances between the supply center and each plant protection operational areas. To address this intricate issue, we propose a hybrid optimization approach combining the K-means clustering and heuristic optimization algorithms. The methodology unfolds in two phases: initially, the K-means algorithm partitions the tea fields, and then supplemental locations and UAV schedules are determined based on workload. Subsequently, the heuristic algorithm orders the operational sequences within each sector, formulating the UAVs’ planned routes.

### 2.2 Efficient hierarchical clustering assignment algorithms

#### 2.2.1 Partitioning of operational areas using clustering algorithms

The division clustering method primarily computes distances between sample data to facilitate uniform partitioning of dispersed field vertices based on proximity (Das et al., 2023; Kuo et al., 2016). This study employs the K-means clustering algorithm to segment tea fields into sub-areas and strategize the replenishment locations according to workload. Silhouette coefficient, as defined in Equations 5 and 6, are calculated to ascertain the optimal number of clusters, thereby enhancing UAV operational efficiency and partitioning quality.


(5) $$S(i)=\frac{b(i)-a(i)}{max(a(i),b(i))}$$

(6) $$a(i)=\frac{a_{1}+a_{2}+a_{3}\ldots a_{n}}{n_{x}},\;b(i)=\frac{b_{1}+b_{2}+b_{3}\ldots b_{n}}{n_{y}}$$

Where a(i) is the denseness of clusters; b(i) is the dispersion between clusters; S(i) is the silhouette coefficient; $n_{x}$ is the count of vertices within the data of a specific cluster; $n_{y}$ is the count of vertices within the nearest neighboring cluster.

#### 2.2.2 Resupply location planning algorithm

For selecting supply points post-clustering for each plant protection partition, it is essential to consider the return points optimally. As delineated in Equations 7 and 8, the planning of supply points is conceptualized as an optimization problem. The objective is to minimize the aggregate distance from the target point coordinates *c* to the surrounding point coordinates $c_{i}$ , thereby identifying the most proximal points. The algorithm’s pseudo-code flow is detailed in Appendix A.


(7) $$f=\min\sum_{i=1}^{n}D_{{\text{c}}_{i}\text{c}}$$

(8) $$D_{c_{i}c}=\sqrt{{\left(x_{i}-x\right)}^{2}+{\left(y_{i}-y\right)}^{2}}$$

Where $D_{{\text{c}}_{i}\text{c}}$ is the distance from random point coordinates to the target point, m; $c_{i}$ is the position of the discrete point; c is the target position; $x_{i}$ , $y_{i}$ are the coordinates of the discrete point; x,y are the coordinates of the target point.

#### 2.2.3 Allocation of unmanned aerial vehicle operational sorties

Prior assessment of pest severity or crop fertilizer requirements allows for the pre-setting of the UAV’s spraying flow rate per hectare F, operating speed v, and width d. The operational area formula is presented in Equation 9, while the UAV payload formula is detailed in Equation 10.


(9) $$\left\{ \begin{array}{l} R=\sum_{1}^{n}R_{1}+R_{2}+\ldots R_{n}\\ S=Rd \end{array}\right.$$

(10) $$L_{j}=\frac{S_{n}-S_{n-1}}{dv}F$$

Prior assessment of pest severity or crop fertilizer requirements allows for the pre-setting of the UAV’s spraying flow rate per hectare *F*, operating speed *v*, and width *d*. The operational area formula is presented in Equation 9, while the UAV payload formula is detailed in Equation 10.

Where, *R* is the total range of plant protection operations on fields, m; *n* is the number of UAV sorties, sorties; *S* is the total area of UAV operations, hm^2^; $S_{n}$ is the area covered in the nth sortie, hm^2^; $S_{n-1}$ is the area covered in the (n−1) th sortie, hm^2^; $L_{j}$ is the payload of the *j*th sortie kg; *F* is the flow rate of spraying L/min.

Utilizing the optimal route from the coverage algorithm, the workload for each plant protection area and the requisite load per sortie are computed, with quantitative loading guided by Equation 10. Given that the operational area may not be an exact multiple of a single sortie’s area, pre-allocation of the load and strategic arrangement of racks and payload are essential to conserve energy and minimize sorties. Equation 11 outlines two methods for rack allocation.


(11) $$j=\left\{ \begin{array}{l} 1,S_{m}<S_{d}\\ \mathrm{int}(\frac{S_{m}}{S_{d}})+1,\frac{S_{m}}{S_{d}}\notin Z,S_{m}\geq S_{d} \end{array}\right.$$

Where $S_{d}$ is the maximum area covered in a single UAV sortie in hectares, hm^2^; $S_{m}$ is the cumulative area of plant protection operations within the operational area, hm^2^; *Z* is a positive integer.

As stipulated in Equation 11, there are two scenarios for UAV sortie allocation:

1. If the operational area is less than the maximum coverage of a single sortie, the UAV does not require recharging.
2. If the operational area exceeds the maximum coverage, the UAV must recharge at least once. The number of flights is the quotient of the operational area and the maximum single sortie area, rounded to the nearest whole number. Typically, the number of flights ranges from 1 < j < int $(\frac{S_{m}}{S_{d}})+1$ .
3. The algorithm’s pseudo-code flow is provided in **Appendix B**.

### 2.3 Design of route planning algorithm for multi-tea field scheduling

#### 2.3.1 Optimization of genetic algorithms

To effectively encode the characteristic points of tea fields, this study utilizes integer coding (Asim and Abd El-Latif, 2023; Yan et al., 2024). Within genetic algorithms, the fitness function gauges an individual’s environmental adaptability, with higher values denoting superior individuals, that is, shorter routes. Here, we consider a coded chromosome represented by | k_1_ | k_2_ |… | k_i_ |… | k_n_ |, with its fitness function defined in Equation 12.


(12) $$f_{n}=\frac{1}{\sum_{i=1}^{n}D_{k_{i}k_{j}}}$$

Where the $D_{k_{i}k_{j}}$ is the distance from operational field *i* to operational field *j*, m; $f_{\text{n}}$ is the reciprocal of the distance to the start vertex after completing the circuit.

To enhance the genetic algorithm’s search efficiency across various evolutionary stages, this study introduces the Hyperbolic Tangent Mapping Crossover and Fitness-Inverse Adjusted Mutation, predicated on fitness values, as delineated in Equations 13 and 14:


(13) $${P^{\prime }}_{c}=\{\begin{array}{ll} \frac{1}{2}(1+\tanh(\frac{2f^{\prime }-f_{\min}-f_{\max}}{f_{\max}-f_{\min}})), & f'⩾f_{avg}\\ 1, & f'<f_{avg} \end{array}$$

(14) $$P_{m}=\{\begin{array}{c} \frac{k_{2}(f-f_{\min})}{f_{\max}-f_{avg}},\;f\geq f_{avg}\\ \frac{k_{3}}{f},\;f<f_{avg} \end{array}$$

Where f' is the mean fitness value of the individuals undergoing crossover; $f_{\text{min}}$ , $f_{\max}$ are the minimum and maximum fitness value respectively; $k_{1}$ is a constant set to 0.5; *f* is the variant individual’s fitness; $k_{2}$ , $k_{3}$ are constants; $k_{2}$ ∈[0,0.001]; $k_{3}$ =0.2.

In the early stages of the algorithm, exploration of new solution regions yields more varied results. To enhance optimization efficiency during this phase, we directly apply the crossover operation ( ${P^{\prime }}_{c}=1$ ) to less adapted routes. Additionally, we set a fixed variance probability ( $k_{3}$ =0.2) inversely related to fitness. This encourages less adapted individuals to explore new solutions aggressively, preventing premature convergence to local optima. Furthermore, we prevent computationally expensive over-exploration by highly fit individuals within better candidate solutions.

As the algorithm progresses, the adaptability of route results improves in later stages. Our study aims to strike a balance between diversity (highly adapted individuals) and retention (better-adapted individuals). To achieve this, we introduce a hyperbolic tangent function with Steeper Gradient and Smoothness properties during the mid- and late algorithm stages. This function controls the transition from high to low crossover probability. Additionally, we dynamically adjust the difference between an individual’s maximum fitness and average fitness, fine-tuning it to the mutation probability using a small interval ( $k_{2}$ ∈ [0, 0.001]). Our designed crossover and mutation operators adapt the population’s fitness distribution across different periods, promoting efficient convergence to the optimal solution.

#### 2.3.2 Optimization of simulated annealing algorithms

In the standard simulated annealing approach, a new solution is accepted if it results in a decrease in system energy; otherwise, its acceptance is determined by a predefined probability (Li et al., 2022a; Sajid et al., 2022). This acceptance criterion dictates the transition probability between the current and new solutions, impacting the algorithm’s optimization performance and convergence velocity. Accordingly, this paper proposes an optimized Metropolis criterion, formulated in Equations 15 and 16.


(15) $$P=\{\begin{array}{ll} 1, & E_{t+1}<E_{t}\\ p=e^{-\frac{E_{t+1}-E_{t}}{T_{m}T}}, & E_{t+1}\geq E_{t} \end{array}$$

(16) $$T_{m}=e^{-\frac{S(x_{\max})-S(x_{\min})}{T_{0}}}$$

Where $E_{t+1}$ , $E_{t}$ are the new and preceding solutions, respectively; $T_{m}$ is the temperature adjustment coefficient; *T* is the current temperature; $S(x_{\max})$ , $S(x_{\min})$ are the maximum and minimum values of the objective function corresponding to the N feasible solutions randomly selected from the top 20% of the pre-algorithmic solution set, respectively; $T_{0}$ is the initial temperature.

If the solution set derived from the genetic algorithm serves as the initial solution for the simulated annealing algorithm, the discrepancy between $E_{t+1}$ and $E_{t}$ may not correspond to the current temperature *T*, potentially leading to an impractical selection probability under the Metropolis criterion. This discrepancy can influence the algorithm’s optimization efficacy and computational duration. The dynamic genetic algorithm introduced in this study adjusts the Metropolis criterion with $T_{m}$ by equating the extreme difference $S(x_{\max})-S(x_{\min})$ of the feasible solutions within the top 20% of the solution set to $T_{0}$ , enabling the Metropolis criterion to adapt to solution space fluctuations and maintain the stability of probability *P*.

In the adaptive Metropolis mechanism, the cooling rate must be modulated based on the proportion *ΔN* of suboptimal solutions accepted in the inner loop. Equation 17 demonstrates that *ΔN* assesses the appropriateness of the cooling rate; a value approaching 0 implies excessive rapidity necessitating a decrease, whereas a value nearing 1 suggests insufficient speed, requiring an increase to enhance algorithmic efficiency.


(17) $$\Delta N=\frac{N_{2}}{N_{1}}$$

Where ΔN is the cooling rate; $N_{2}$ is the count of inferior solutions accepted within $N_{1}$ iterations; $N_{1}$ is the total number of instances where $E_{t+1}\geq E_{t}$ at temperature *T*.

Given the complexity of the UAV plant protection scheduling problem with multiple traveling salesman problem studied in this paper, an efficient parallel algorithm is needed to solve the traveling salesman problem in each partition. Thus, this paper introduces an adaptive cooling function that amalgamates logarithmic and exponential functions to define the temperature function, as depicted in Equations 18 and 19.


(18) $$T_{k}=\frac{T_{s}}{\log(k)},k\leq m$$

(19) $$T_{k}=\{\begin{array}{ll} T_{s}*0.99^{k}, & k>m,\Delta N\in (0.75,1)\\ \frac{T_{s}}{\log(k)}, & k>m,\Delta N\in (0,0.25) \end{array}$$

Where *m* is a predetermined constant; $T_{s}$ is the set initial temperature; *k* is the current number of iterations.

**Figure 4:**
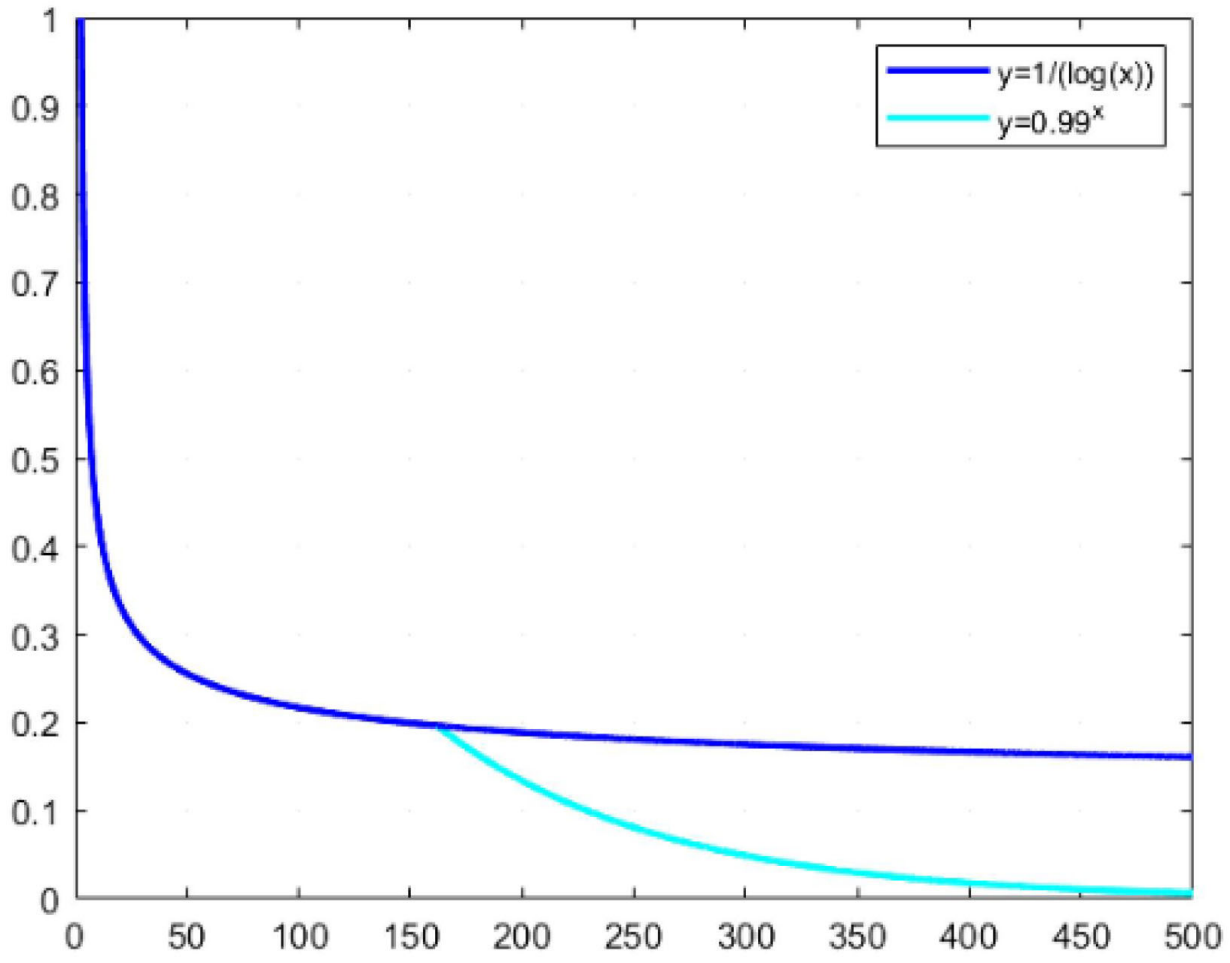
Optimized cooling curve intervals for the simulated annealing algorithm.

As shown in **Figure 4**, the intersection of the logarithmic and exponential functions determines m, with the derivation of the logarithmic function yielding *m* = 161.8473. Initially, the algorithm employs the logarithmic function for swift cooling to approximate the optimal solution’s vicinity. Subsequently, the cooling magnitude is dynamically adjusted based on the acceptance rate of suboptimal solutions *ΔN*, with real-time modulation of the cooling pace achieved through alternating use of the exponential and logarithmic functions.

### 2.4 Design of the algorithmic fusion approach

**Figure 5:**
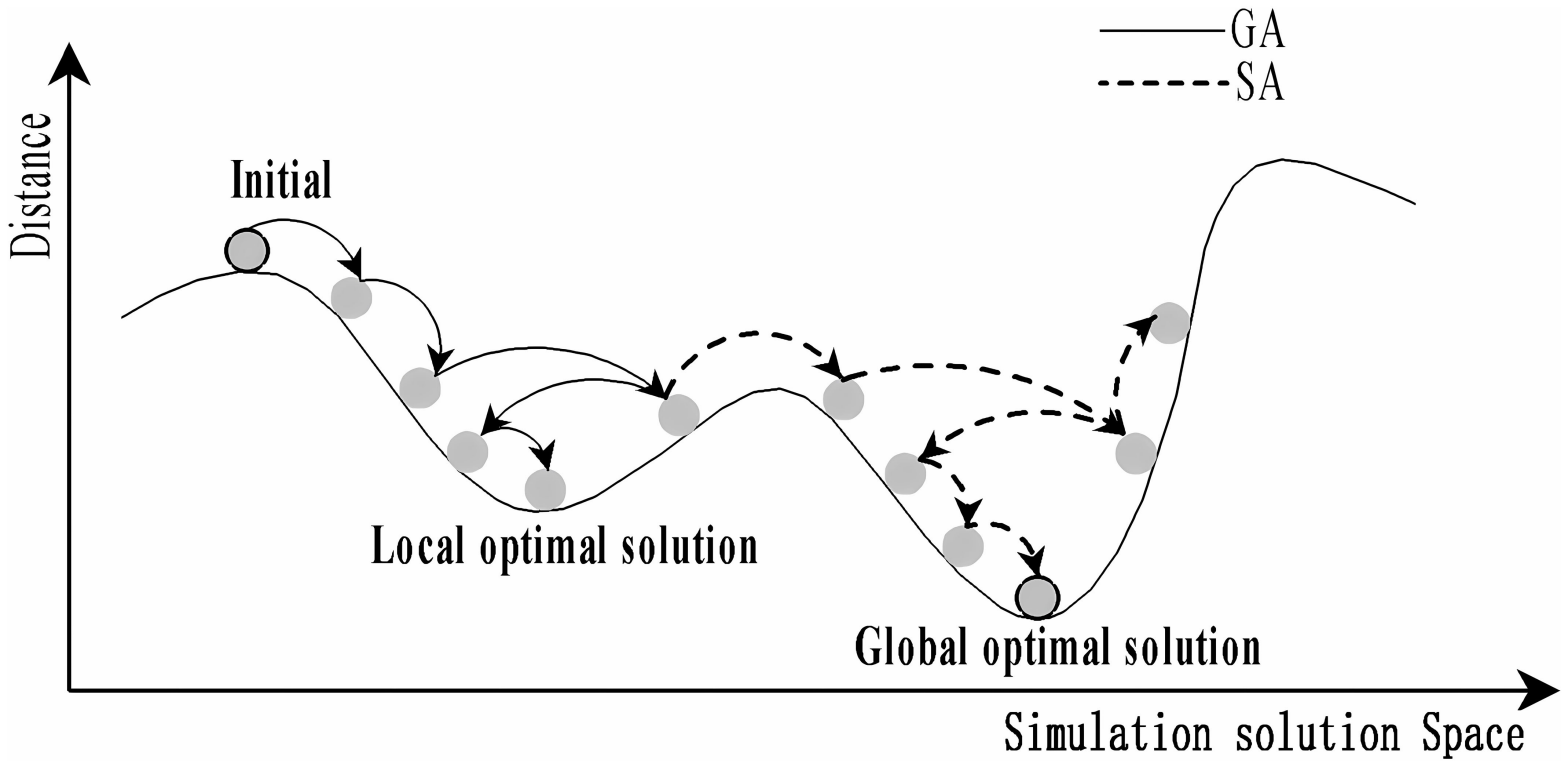
Schematic representation of the solution space for Genetic Algorithm (GA) and Simulated Annealing (SA).

**Figure 5** illustrates the optimization search processes of the genetic algorithm (GA) and the simulated annealing algorithm (SA), likened to a mountain climbing strategy. In GA, an individual’s position, indicated by a solid arrow, often converges prematurely to a local optimum. This paper integrates the concept of simulated annealing into GA, as depicted by dashed arrows, which promotes the pursuit of the global optimum by probabilistically pushing GA beyond the local minima through the jump mutation property of adaptive annealing itself (Marjit et al., 2023; Ozkan, 2021).

**Figure 6:**
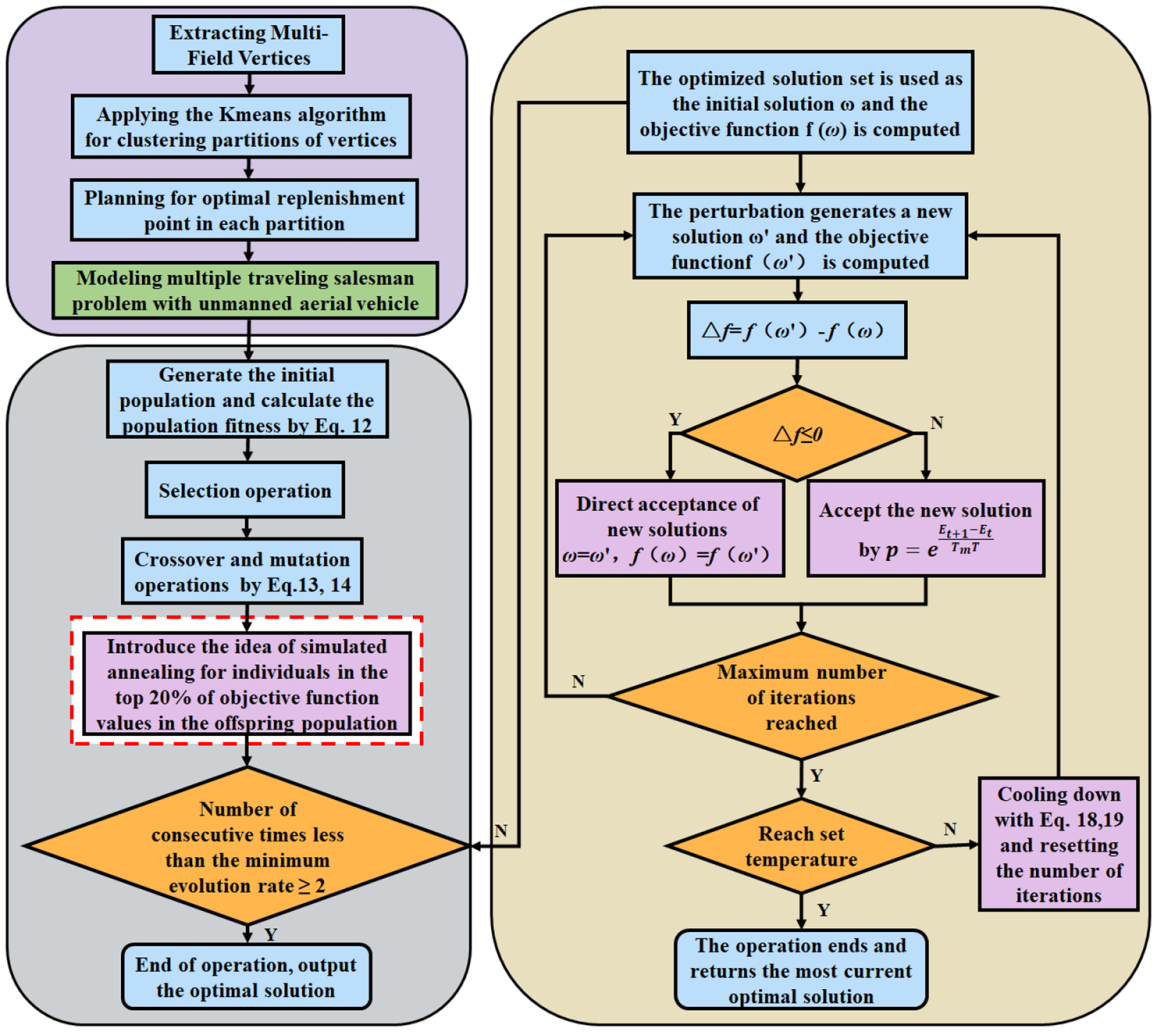
Search flowchart of Adaptive Clustering Hyperbolic Annealing Genetic Algorithm (ACHAGA).

Upon examining the optimization trajectories of GA and SA, it becomes evident that they offer complementary advantages in exploring the solution space’s breadth and depth. This synergy suggests the potential for a hybrid approach, leveraging GA’s prowess in global search and SA’s finesse in local refinement (Santé et al., 2016). The dynamic crossover and mutation mechanisms in GA promote robust, undirected population evolution, while SA’s targeted filtering fine-tunes the search direction. This study proposes amalgamating this enhanced algorithmic framework with clustering techniques and supply point strategies to devise a proficient planning method tailored to the multiple traveling salesman problem with unmanned aerial vehicle (MTSPU) in multi-tea field scenarios. The workflow of the ACHAGA fusion algorithm is presented in **Figure 6**.

### 2.5 Design of the experiment

**Figure 7:**
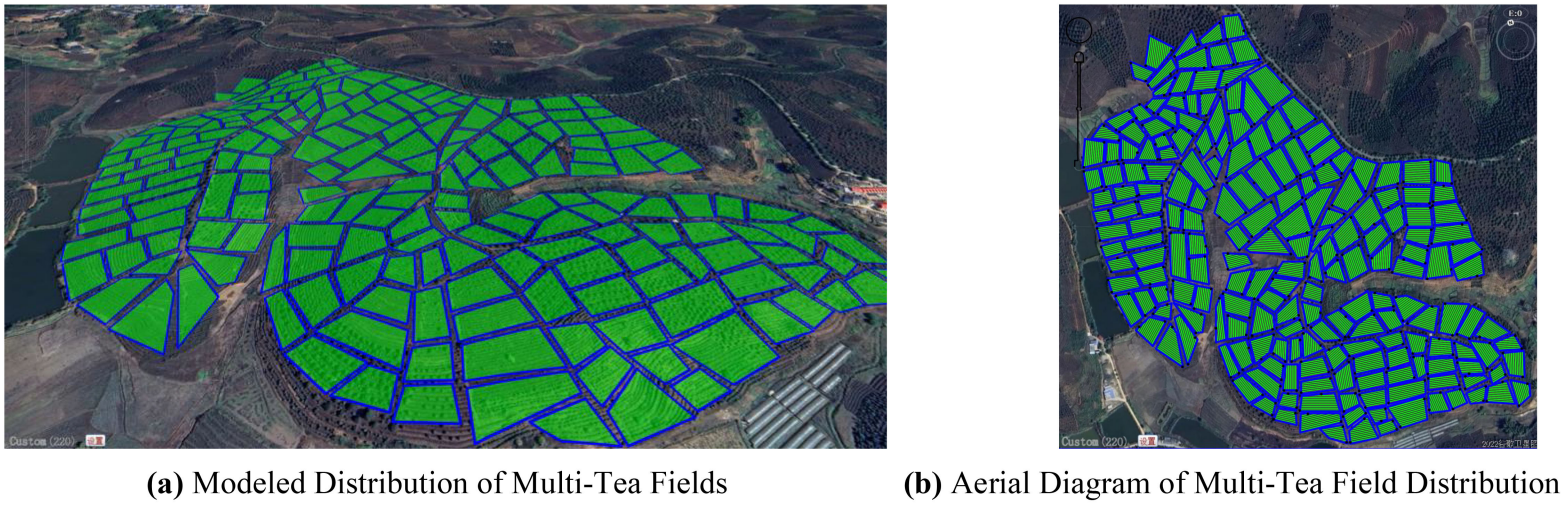
Schematic of the model distribution for the multi-tea field problem. **(A)** Modeled Distribution of Multi-Tea Fields. **(B)** Aerial Diagram of Multi-Tea Field Distribution.

In this experiment, the DJI T20p plant protection UAV served as a model to simulate and evaluate its operational efficiency within a tea cultivation region in Dadugang Township, Jinghong City, Xishuangbanna Prefecture, Yunnan Province, China. As depicted in **Figure 7A**, the selected tea cultivation area spans from 100°43’ to 101°12’ east longitude and 22°30’ north latitude, encompassing 200 tea fields with a cumulative area of 28.2 hectares (hm²), averaging 0.3 hm² per field. The T20p’s operational efficiency, as per official specifications, is 1.67 hm² per flight. For this analysis, the tea regions were digitally rendered using OWI 3D mapping software (Version 9.1.6 X64), illustrated in **Figure 7B**, where the blue bold line demarcates the tea field boundaries, and the green shaded regions represent the fields themselves.

#### 2.5.1 Adaptive clustering hyperbolic annealing genetic algorithm for multi-field plant protection operation scheduling route simulation experiments

1. This study aims to evaluate the enhancements made to the Hyperbolic Genetic Algorithm (HGA) and the Adaptive Simulated Annealing (ASA), and to assess the performance of the newly developed Adaptive Clustering Hyperbolic Annealing Genetic Algorithm (ACHAGA). To achieve this, we conducted a comparative evaluation via computer simulation, analyzing the search accuracy and optimization capabilities of each algorithm iteration. The simulations were performed on a test PC with an Intel(R) Core(TM) i7-6700HQ CPU @ 2.60GHz, 8 GB RAM, running on a Windows 10 platform using Matlab-2018a, focusing on the multi-tea field problem model. The selected hardware and software specifications ensure a balance between computational efficiency and accessibility, providing a realistic benchmark for practical applications. For consistency, the maximum number of iterations was set at 200 and the population size at 50. These parameters were chosen based on preliminary tests that indicated they provide a good balance between computational time and optimization performance. A higher number of iterations allows the algorithm to explore the solution space more thoroughly, increasing the likelihood of finding a global optimum. Meanwhile, a population size of 50 ensures sufficient genetic diversity without overwhelming computational resources. Additional algorithm parameters are detailed in **Table 1**, which includes specifics on mutation rates, crossover probabilities, and the cooling coefficient for the ASA component. These parameters were fine-tuned through a series of preliminary experiments to ensure optimal performance of ACHAGA in solving the multi-tea field problem. The software specifications are listed in **Table 2**.

**Table 1:** Various algorithm parameters.

Algorithm	Parameters
AFSA	Population size: n=100, Maximum number of iterations: Max_gen=200, Maximum number of attempts: trynumber=500, Perception distance: Visual=16, Crowding factor: deta=0.8
BSO	Population size: n=100, Number of clusters: cluster_num=x, Maximum number of iterations: Max_gen=200, Probability of selecting one cluster: p_one=0.5, Probability of selecting two clusters: p_two=1-p_one
GA-ACO	Population size: n=100, Maximum number of iterations: Max_gen=200, Crossover probability: Pc=0.8, Mutation probability: Pm=0.2, Importance of pheromone: Alpha=1, Importance of heuristic factor: Beta=5, Pheromone enhancement coefficient: Rho=0.1, Pheromone enhancement coefficient: Q=100
ACHAGA	Population size: n=100, Crossover probability: as in Equation 13, Mutation probability: as in Equation 14, Maximum number of iterations: Max_gen=200, Initial temperature: T0 = 100, Final temperature: Tend=1e-8, Selection probability: as in Equation 15, Cooling coefficient: alpha as in Equations 18, 19
GA	Population size: n=100, Crossover probability: Pc=0.8, Mutation probability: Pm=0.2, Maximum number of iterations: Max_gen=200

**Table 2:** Test platform and software version.

Test platforms	Platform configuration parameters
CPU	Intel(R) Core(TM) i7-6700HQ CPU @ 2.60GHz, 2.59 GHz
GPU	GTX 1060 6GB
RAM	8GB
Operating system version	Windows-10
Software version	Matlab-2018(a)

**Figure 8:**
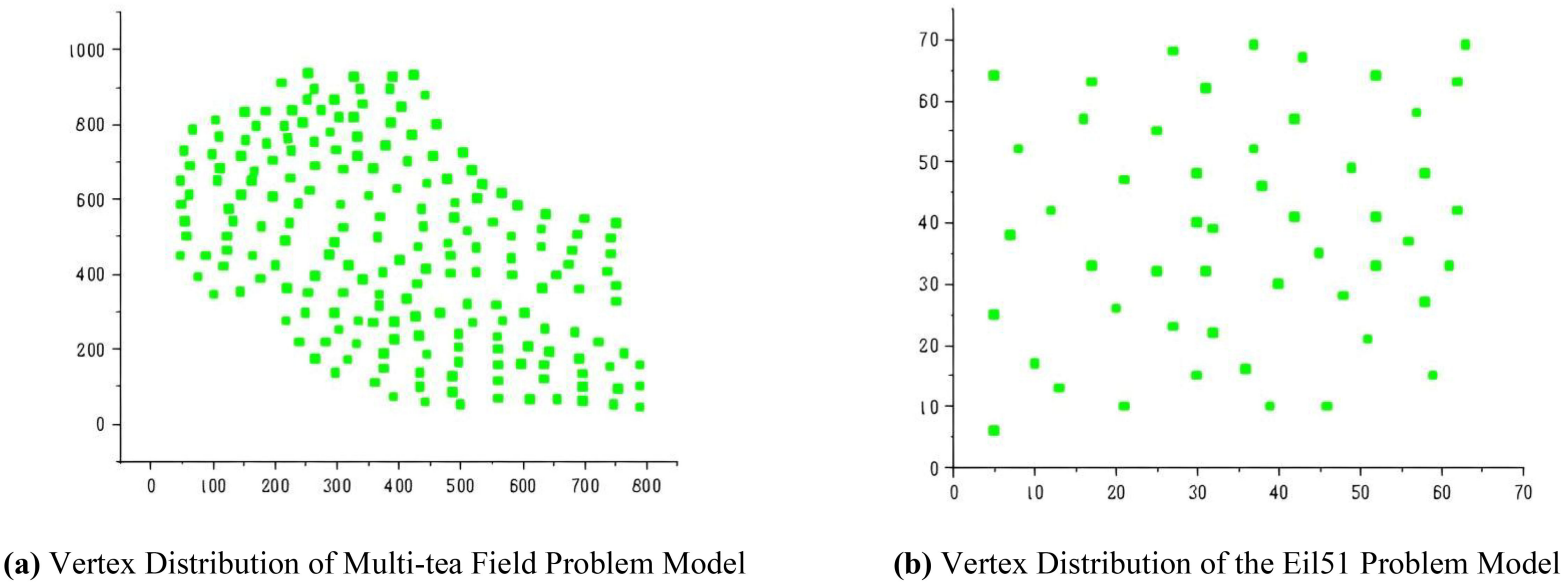
Problem objects of the optimization performance test. **(A)** Vertex Distribution of Multi-tea Field Problem Model. **(B)** Vertex Distribution of the Eil51 Problem Model.

2. To evaluate the optimization efficacy of the proposed ACHAGA on the multi-tea field problem model, it was benchmarked against established swarm intelligence algorithms, namely the Brainstorming Algorithm (BSO) and the Artificial Fish Swarm Algorithm (AFSA) (Qiu et al., 2015; Neshat et al., 2014). Each algorithm underwent 20 trials, maintaining identical iteration and population constraints as specified in **Table 1**, with the mean outcome representing the final result. As illustrated in **Figures 8A, B**, two distinct test scenarios were selected: a self-constructed 200-tea field problem model and the eil51 problem from the TSPLIB standard database, featuring uniformly distributed nodes. The eil51 nodes were presumed to be ideal tea field vertices, averaging 0.3 hectares (hm²) per vertex, totaling 15.3 hm².

**Figure 9:**
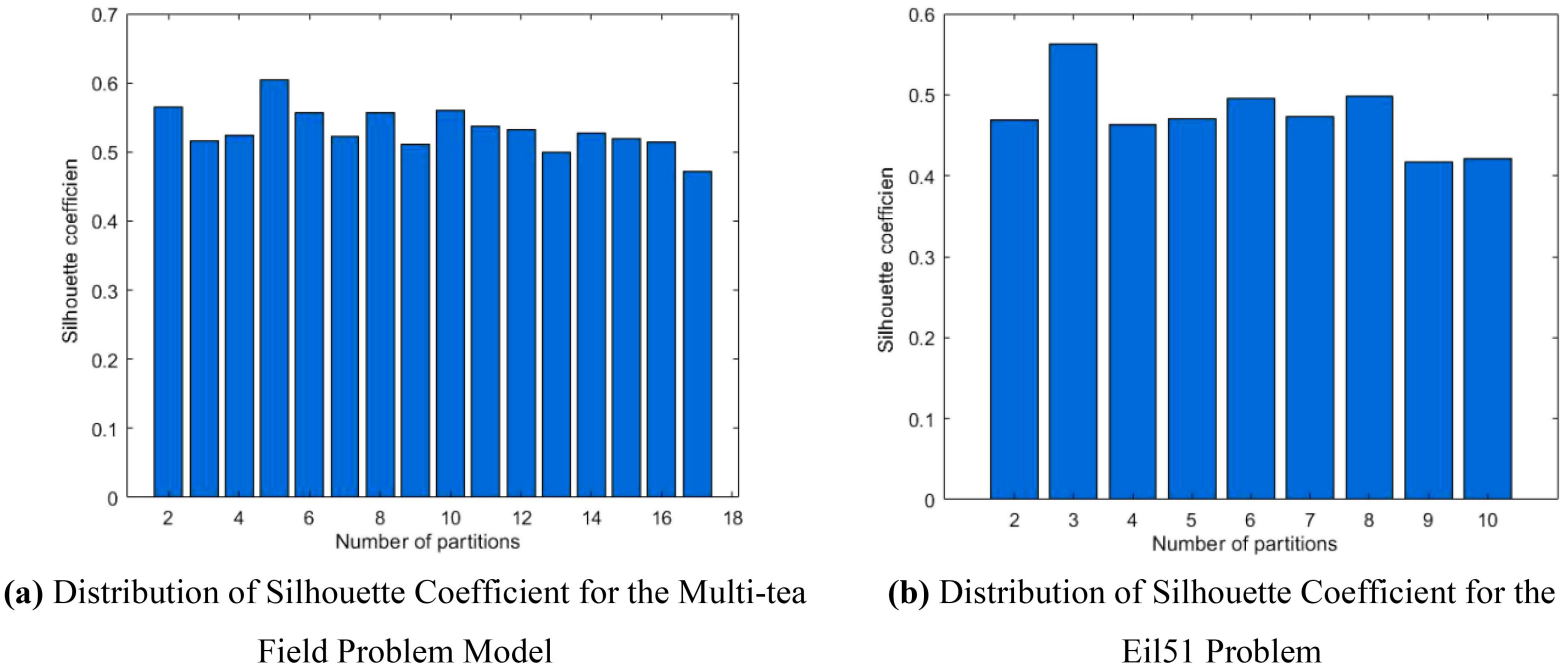
Comparative histogram of silhouette coefficient for each problem. **(A)** Distribution of Silhouette Coefficient for the Multi-tea Field Problem Model. **(B)** Distribution of Silhouette Coefficient for the Eil51 Problem.

To optimize the preprocessing clustering division scheme for simulation trials of the multi-tea field problem model and the eil51 problem, it is necessary to determine the optimal silhouette coefficient distributions across various UAV sortie ranges, depicted in **Figures 9A, B**. The UAV sortie range is calculable from Equation 11, considering the UAV’s working efficiency ( $S_{d}$ ) and the working area ( $S_{m}$ ) for each problem, as detailed in Section 2.5. The silhouette coefficient distributions for the refined multi-tea field problem model and the eil51 problem fall within the ranges of (1, 17] and (1, 10], respectively.

**Figure 10:**
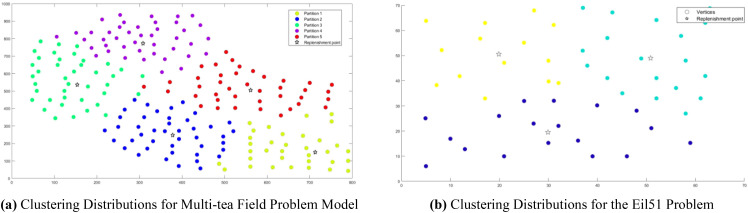
Clustering Distributions for Multi-tea Field Problem Model and Eil51 Problem. **(A)** Clustering Distributions for Multi-tea Field Problem Model. **(B)** Clustering Distributions for the Eil51 Problem.

Analysis of **Figures 9A, B** indicates that the silhouette coefficient for both problem types peak at k = 5 and k = 3. Consequently, the k-means algorithm was utilized to segment the two problem types into 5 and 3 plant protection work areas, respectively, as shown in **Figures 10A, B**. In these figures, dots represent vertices within the tea fields, colors denote work zones, and stars symbolize supply points.

3. To evaluate the effectiveness of the ACHAGA algorithm on the multi-tea field problem model and the eil51 problem, a Wilcoxon rank-sum test was conducted to perform a non-parametric statistical comparison with four other related algorithms (Kochengin et al., 2019). A significance level of α= 5% was established; a p-value less than 5% signifies a statistically significant difference, while a value greater than or equal to this threshold indicates no significant difference. To provide a more rigorous statistical analysis, confidence intervals were calculated to estimate the precision of the observed effect sizes. The effect sizes were also reported to quantify the magnitude of the differences between the ACHAGA algorithm and the other algorithms.

#### 2.5.2 Multi-tea field plant protection site test based on adaptive clustering hyperbolic annealing genetic algorithm

**Figure 11:**
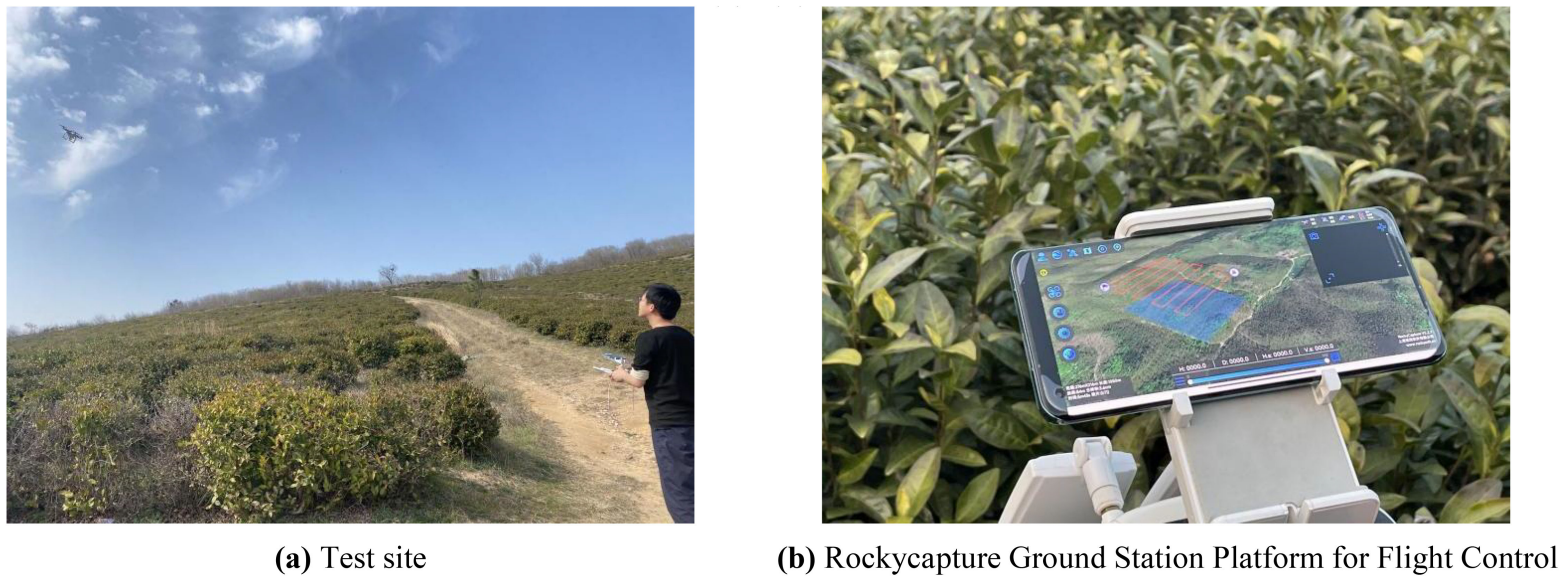
Field validation tests. **(A)** Test site. **(B)** Rockycapture Ground Station Platform for Flight Control.

This study introduces an algorithm designed for path planning across multi-tea field in hilly, mountainous terrains. To validate its feasibility and accuracy, the algorithm was evaluated based on four criteria: operational voyage, coverage, excess coverage, and dispatch path length. Additionally, a field trial was conducted to compare the actual operating conditions with simulation results and to assess the experimental design’s soundness. Drawing on the methodologies of scholars both domestic and international (Tian et al., 2023; Li et al., 2022c), the study utilized a DJI Phantom 4 Pro UAV, boasting a top horizontal velocity of 72 km/h and a maximum endurance of approximately 30 minutes. It is equipped with a 42° tilt capability and a satellite positioning system supporting GPS/GLONASS dual-mode for superior navigational precision. The route planning was facilitated by the RockyCapture flight control system, which offers features such as free planning, one-key control, and real-time tracking, as illustrated in **Figures 11A, B**.

##### 2.5.2.1 Trial Site Conditions

**Figure 12:**
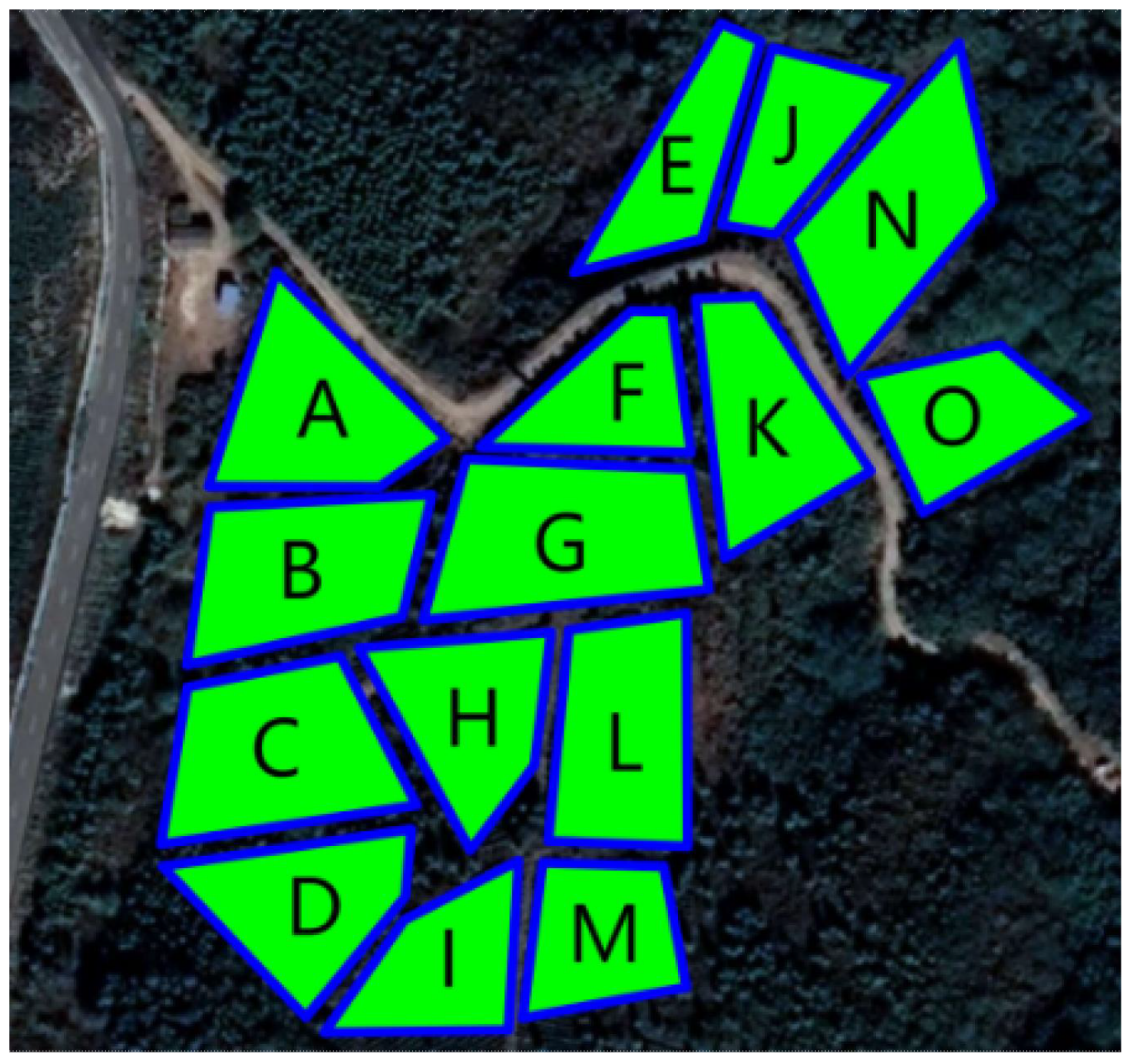
Diagram of the test site.

The trial was conducted from February 16 to 21, 2023, at the Dashanmiao Tea Farm in Shitang Town, Feidong County, Hefei City, Anhui Province, China, featuring a tea plantation with an approximate 7° slope. The weather conditions during the test period included sunny skies, an average temperature of 17°C, relative humidity of 38%, and wind speeds ranging from 1 to 2 on the Beaufort scale. Given the UAV’s operational range limitations, the selected test area comprised 15 fields with an average area of 0.12 hectares (hm²), detailed in **Figure 12**, with the geographical coordinates provided in **Table 3**.

**Table 3:** Longitudinal and latitudinal coordinates of field plots in the test area.

Field	Field apex coordinates	Area (m^2^)	Projected perimeter (m)
A	117°39′47.56″; 31°51′5.65″	1400	161.21
B	117°39′47.50″; 31°51′4.51″	1500	164.29
C	117°39′47.27″; 31°51′3.17″	1600	167.63
D	117°39′47.50″; 31°51′2.06″	1200	155.06
E	117°39′50.75″; 31°51′7.57″	900	152.08
F	117°39′50.22″, 31°51′5.77″	800	134.76
G	117°39′49.73″; 31°51′4.75″	1800	181.25
H	117°39′49.00″; 31°51′3.32″	1000	136.84
I	117°39′48.69″; 31°51′1.54″	900	140.34
J	117°39′51.66″; 31°51′7.61″	800	128.29
K	117°39′51.42″, 31°51′5.62″	1300	157.69
L	117°39′50.20″; 31°51′3.22″	1400	157.75
M	117°39′50.04″; 31°51′1.83″	900	127.84
N	117°39′52.56″; 31°51′7.11″	1600	179.24
O	117°39′53.18″; 31°51′5.63″	1000	133.38

##### 2.5.2.2 Field trial program

**Figure 13:**
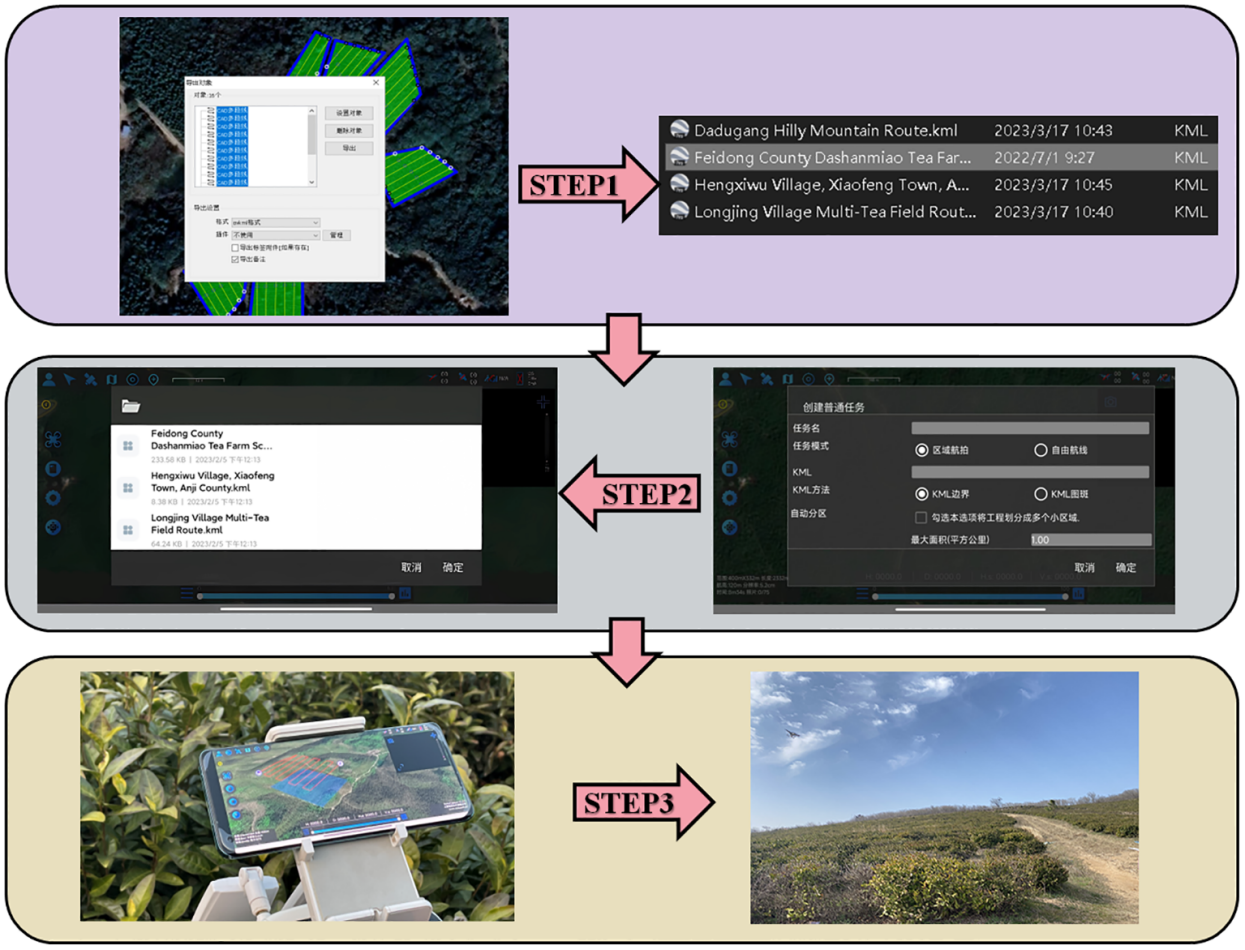
Importing the planned route KML file into the ground station operation process.

The test flowchart, depicted in **Figure 13**, outlines the specific test procedures:

Step 1: Utilizing OWI Interactive Map Software (Version 9.1.6 X64), the test area’s coordinates, boundaries, and elevation data were acquired to establish a planar right-angle coordinate system. Subsequently, the k-means algorithm and Mercator inverse formula were applied to determine and label the test site’s supply points on the map. Each algorithm was then used to devise a corresponding route scheme, culminating in the export of a KML file of the planned route.

Step 2: The ground station flight control platform was accessed to import the route by selecting the import function, locating the recently exported KML file, and integrating it into the platform. The route’s accuracy was verified, parameters and settings were adjusted, and preparations were made for UAV flight control.

Step 3: The flight control platform was employed for testing: the UAV was connected to the ground station flight control platform, initiated, and navigated according to the imported route. The UAV’s status and data were monitored throughout the flight, with test outcomes and issues being meticulously recorded.

##### 2.5.2.3 Pre-Treatment Clustering Division of the Trial Area

**Figure 14:**
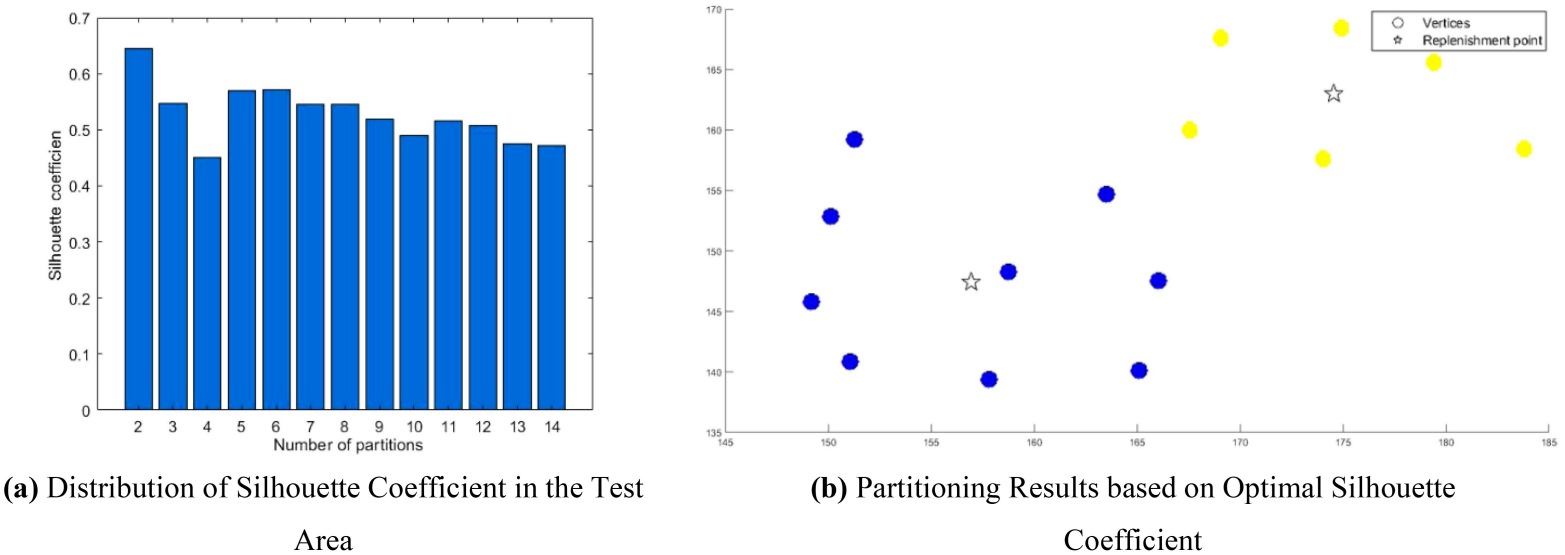
Clustering of the trial area. **(A)** Distribution of Silhouette Coefficient in the Test Area. **(B)** Partitioning Results based on Optimal Silhouette Coefficient.

To enhance the clustering partitioning effect, the study introduced a contour coefficient calculation method prior to partitioning. This yielded a contour coefficient comparison chart for varying cluster numbers k, as illustrated in **Figures 14A, B**.

The analysis of **Figure 14A** reveals that the contour coefficient is maximized when k=2. Consequently, the study opted to segment the multi-tea field into two task areas, achieving the results displayed in **Figure 14B** through clustering division.

##### 2.5.2.4 Trial Methods and Data Calculation

To assess the algorithm’s performance and feasibility, site experiments were conducted for both the test group (BSO) and the control group (ACHAGA). The test group employed manual empirical planning and the brainstorming algorithm, while the control group utilized the clustering division planning algorithm proposed in this study for site operation scheduling route planning. **Table 1** presents the theoretical parameters of the algorithms. Accounting for navigation errors and wind speed effects, five field trials were executed for each algorithm, selecting the optimal voyages and routes for comparative analysis. Subsequently, the optimal route data were derived and subjected to computational analysis.

For evaluating the accuracy of full-coverage routes, the index of excess coverage was employed. The excess coverage rate is calculated using Equation 20, where the product of the total operating range *L* and the operating width *B* constitutes the operating area. In full-coverage operations, a smaller operating area correlates with a lower excess coverage rate, indicating greater accuracy of the coverage area.


(20) $$\eta =(|\frac{LB}{S_{0}}-1|)\times 100\%$$

Where *η* is excess coverage; *L* is the total operational range, m.

## 3 Results

### 3.1 Multi-tea field plant protection site trials using adaptive clustering hyperbolic annealing genetic algorithm

The field trial outcomes are presented in **Tables 4** and **5**. The black line in the planning route column (left side of the table) represents the full-coverage operation route at 2.5-meter intervals, with an operational width set at 5 meters. The pink line indicates the dispatch route, and the ‘H’ point marks the return to the supply point. In the optimal flight path column (left side of the table), the red line signifies the full-coverage operation route, the yellow line the scheduling route, and the coordinates for the two supply points in the south and north are determined using the inverse Mercator projection formula as (117°39′49.31″, 31°51′2.66″) and (117°39′51.31″, 31°51′6.70″), corresponding to the circular charge markers in the north and south, respectively.

**Table 4:** Comparison of various route planning methods and actual flight routes.

Methodology	Planning the optimal route range (m)	Actual flight range (m)
ACHAGA	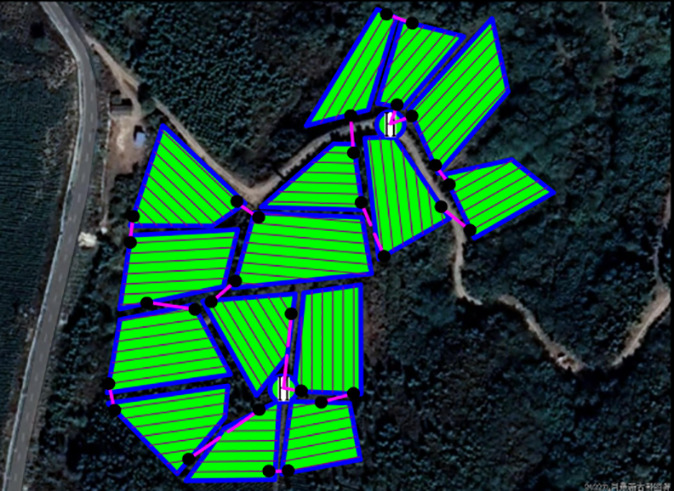	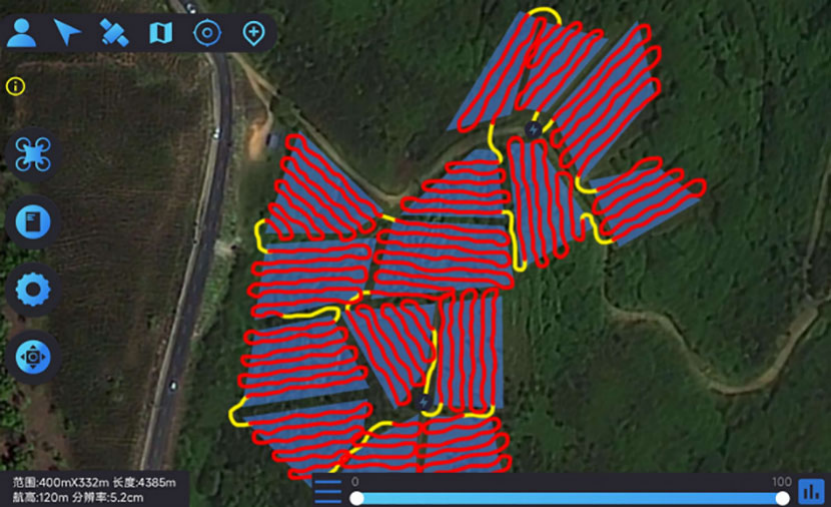
	3861.2	4385.2
BSO	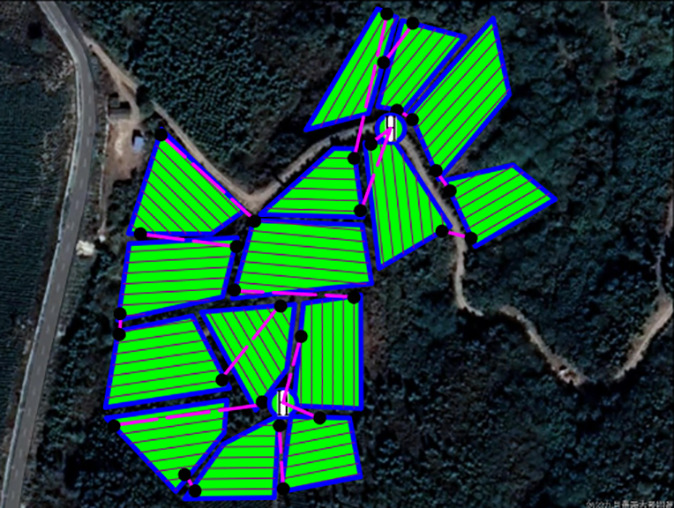	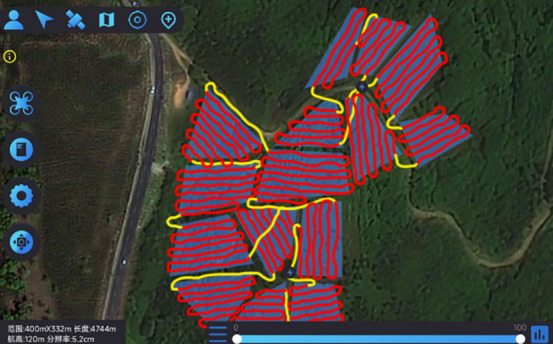
	4275.9	4744.8
AE	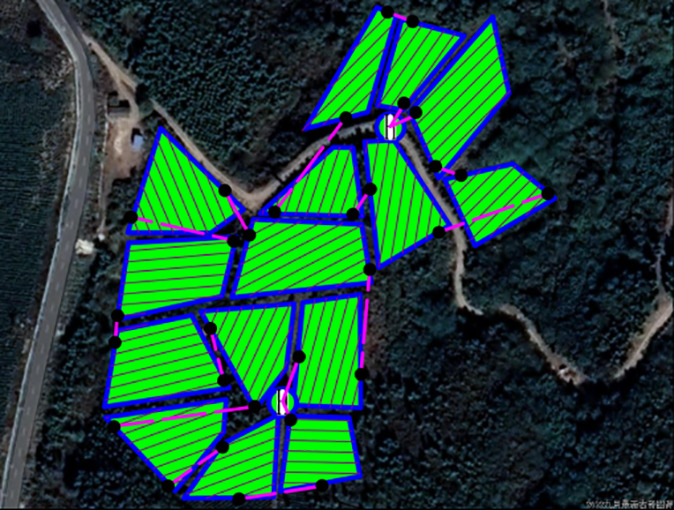	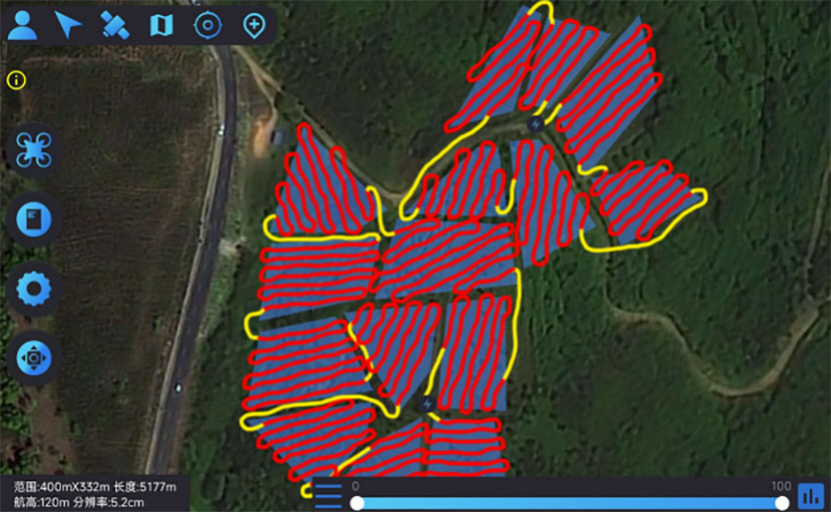
	4358.3	5177.1

**Table 5:** Comparison of optimal results from five planning routes by different methods.

Planning methodology	ACHAGA		AE		BSO	
Export Route	Planned Routes	Flight routes	Planned Routes	Flight routes	Planned Routes	Flight routes
Minimum dispatch range (m)	277.1	322.7	501.9	592.2	591.8	689.1
Number of U-turns (times)	83		94		83	
Percentage of non-operational time	–	8.40%	–	12.60%	–	15.10%
Rate of coverage	–	9.48%	–	13.85%	–	9.36%

## 4 Discussion

### 4.1 Convergence curve performance test of hyperbolic annealing genetic algorithm for adaptive clustering

#### 4.1.1 Robustness and optimization capability analysis of the proposed algorithm’s iterative curve

**Figure 15:**
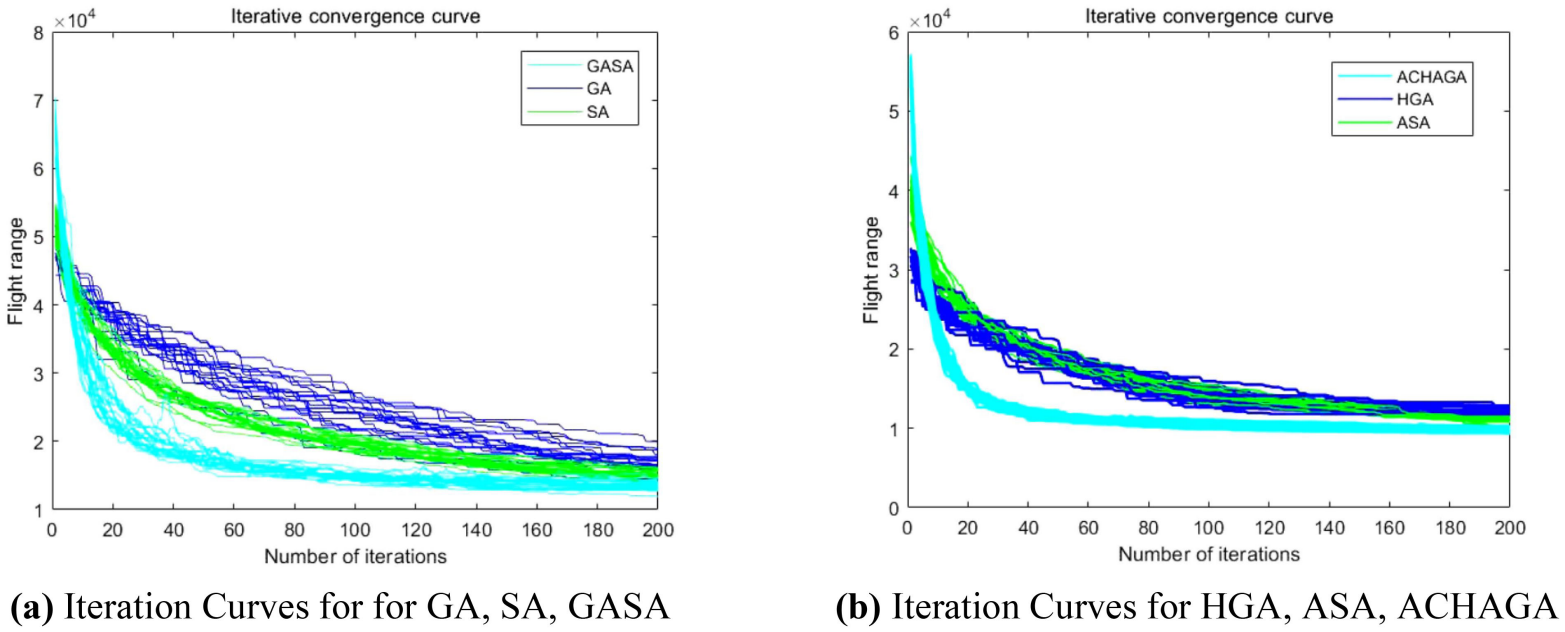
Comparison of convergence curves for each algorithm pre and post optimization Fusion. **(A)** Iteration Curves for GA, SA, GASA. **(B)** Iteration Curves for HGA, ASA, ACHAGA.

The analysis of iteration curves in **Figures 15A, B** demonstrates that both the Hyperbolic Annealing Genetic Algorithm (HGA) and the Genetic Algorithm (GA) exhibit superior optimization efficiency in the initial phase. HGA presents smoother iteration curves and achieves a more refined global optimal solution. This suggests that early direct crossover operations and optimization strategies, such as hyperbolic tangent mapping crossover and fitness-inverse adjusted mutation, expedite the removal of low-fit individuals, curtail ineffective searches, and enhance HGA’s efficiency and stability, thereby broadening its optimization search space. The Adaptive Simulated Annealing (ASA) and Simulated Annealing (SA) algorithms display greater overall optimization capability with minimal fluctuations in the optimal solution. The iteration curves for ACHAGA and GASA are characterized by smoothness and stability, with an observable “discontinuity” in the early stages and a transition from rapid to gradual convergence within the first thirty iterations. This indicates that the ACHAGA algorithm, which integrates HGA and ASA, significantly improves robustness, stability, and optimization efficiency. Furthermore, the intersection of dark blue and green iteration curves in **Figures 15A, B** reveals overlapping segments during the iterative process, particularly between HGA and ASA post-improvement (15-165 iterations). This overlap effectively balances the performance of both algorithms, mitigating the impact of individual algorithm performance and substantiating the efficacy of incorporating hyperbolic genetic algorithm concepts into adaptive annealing, thereby leveraging the strengths of both approaches.

#### 4.1.2 Optimization accuracy and stability analysis of the proposed algorithm

The analysis of iteration curves for each partition, as shown in **Tables 6** and **7**, reveals that while all five algorithms successfully generate flight routes covering all fields, they exhibit significant variance in search accuracy, convergence speed, and iteration performance. The algorithm introduced in this paper demonstrates a pronounced ability to converge to the optimal solution within an average of 40 iterations for the multi-tea field problem model. Notably, the ACHAGA algorithm’s average number of iterations and optimal route lengths are reduced by 113.32 iterations and 1811.93 meters, respectively, compared to other biomimetic algorithms. This efficiency indicates that ACHAGA can achieve superior solutions with fewer iterations. Further examination of **Table 8** indicates that the mean optimal solution lengths for ACHAGA are 9693.27 meters for the multi-tea field problem model and 461.26 meters for the standard eil51 problem. These results are significantly lower than those achieved by the other four biomimetic algorithms. For the multi-tea field problem model, ACHAGA’s results show substantial reductions of 4904.82, 926.07, 3803.96, and 800.11 meters compared to the optimal solutions of GA, Genetic ACO Fusion Algorithm, AFSA, and BSO, underscoring ACHAGA’s considerable advantage in model optimization accuracy. Additionally, ACHAGA exhibits the smallest polar deviation and coefficient of variation in data for both the multi-tea field problem model and the standard eil51 problem, with values of 56.29 and 4.56, and coefficients of variation of 0.00406241 and 0.00326200, respectively. These metrics suggest a low dispersion in test results, affirming that the outcomes are not due to random chance and that the data’s reliability is robust. The narrowest confidence interval for ACHAGA not only suggests more reliable results compared to other algorithms but also confirms its stability in delivering consistent outcomes.

**Table 6:** Optimal routes and convergence curves comparison for clustering partitioning.

Algorithm	Optimal route range (m)	Average number of iterations
AFSA	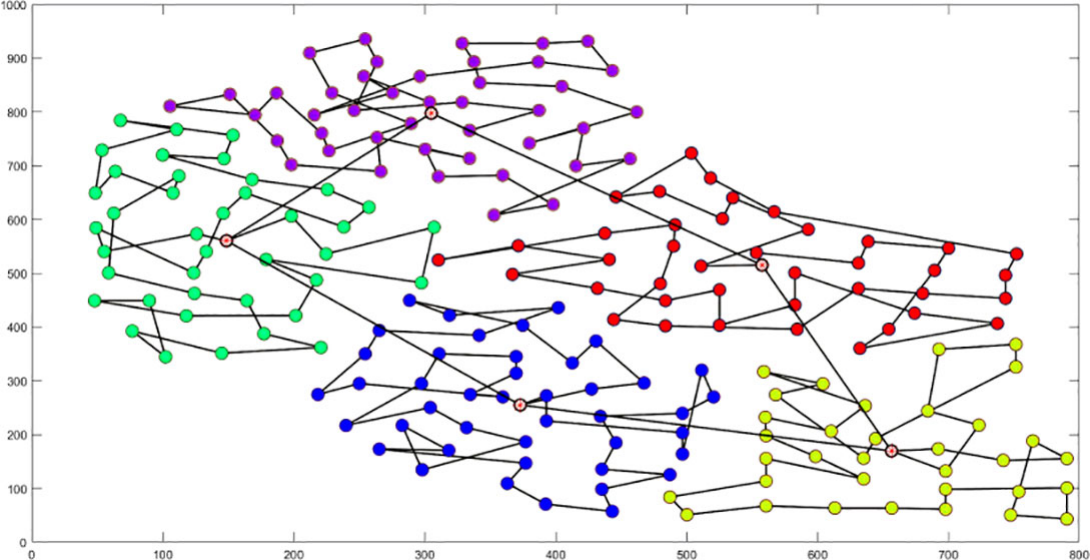 12480.2	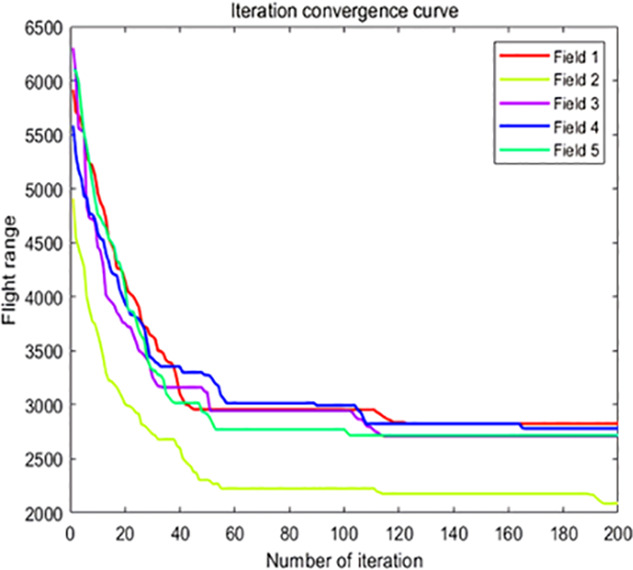 137.6
BSO	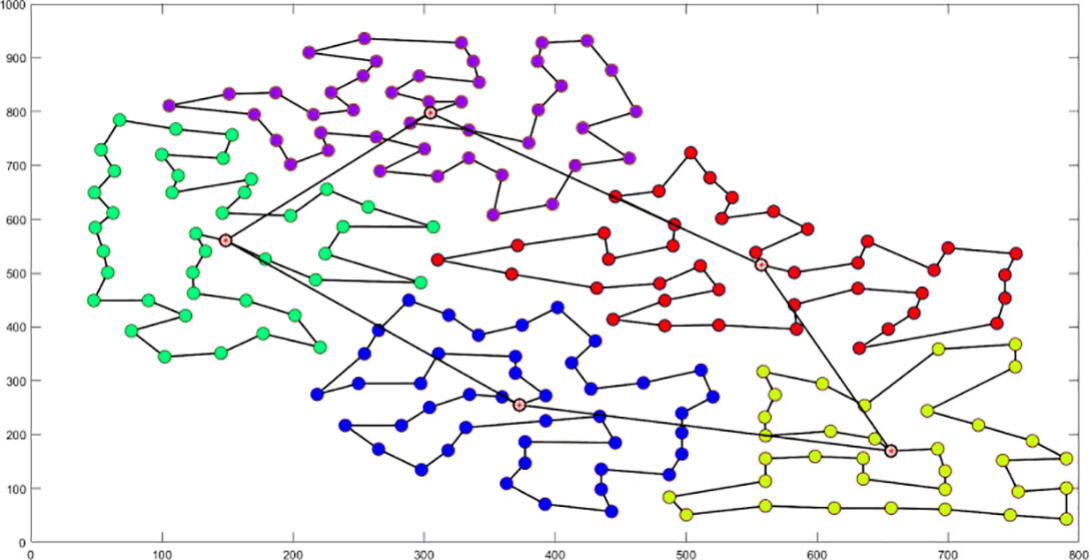 10035.9	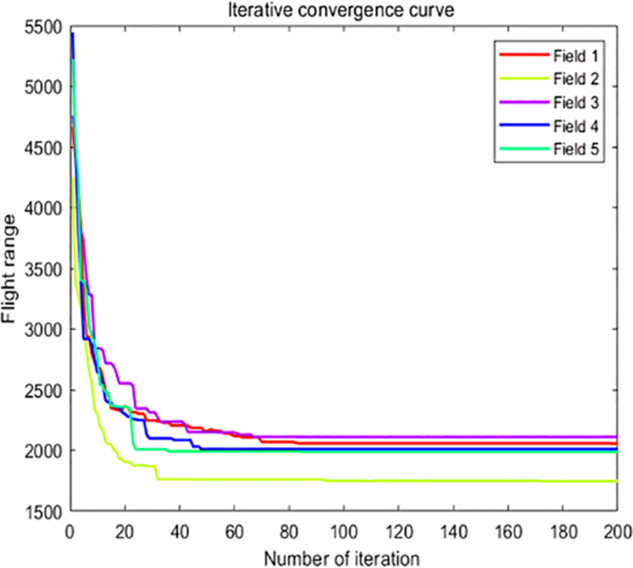 66.2
GA-ACO	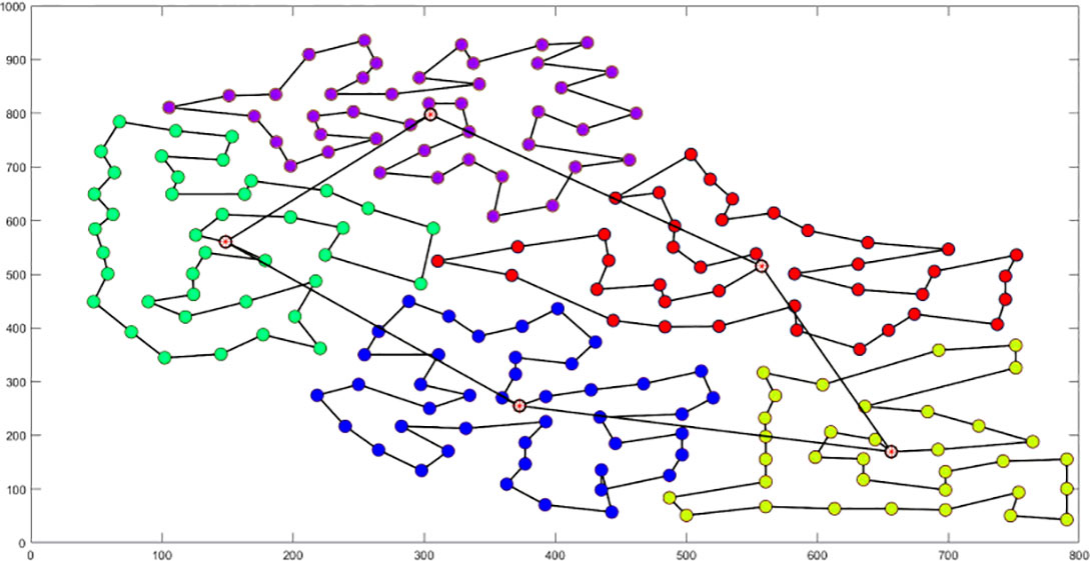 10256.2	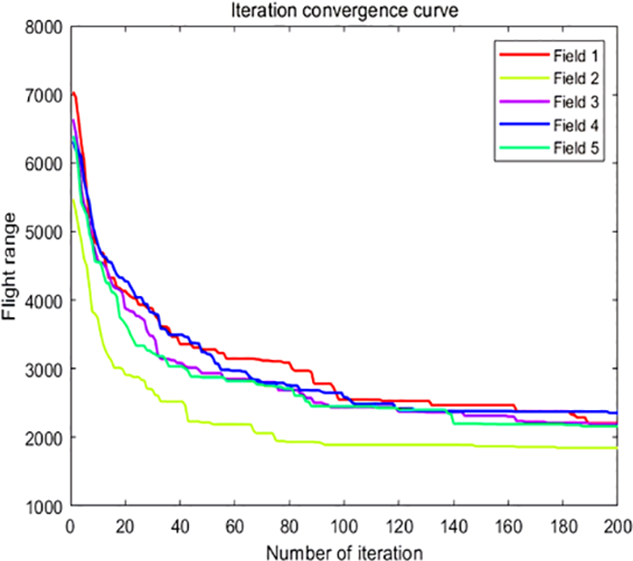 185.6
ACHAGA	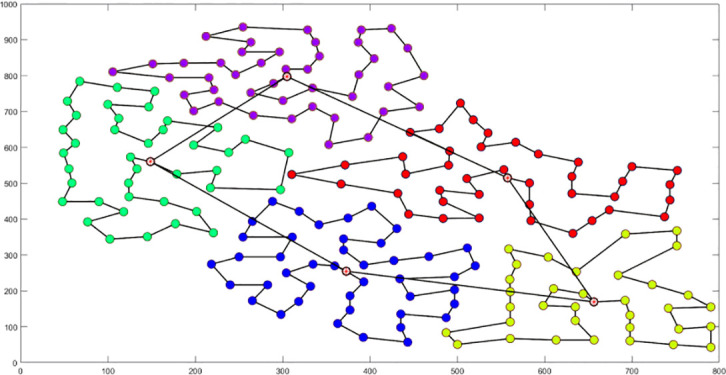 9659.7	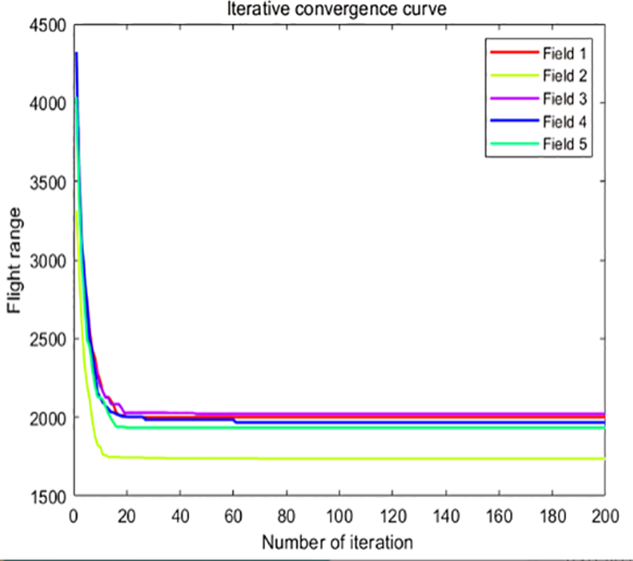 31.8
GA	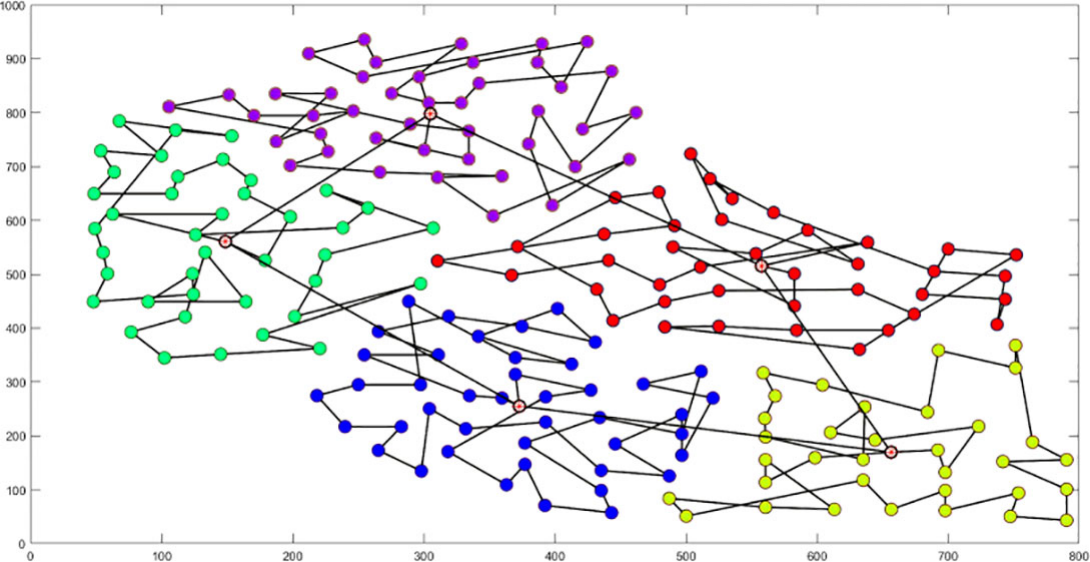 13114.2	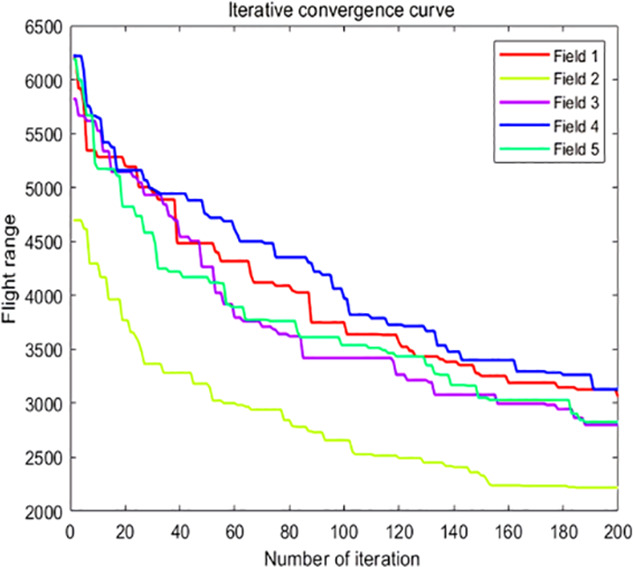 191.1

**Table 7:** Optimal routes and convergence curves comparison for various algorithms’ clustering partitions.

Algorithm	Optimal route range (m)	Average number of iterations
AFSA	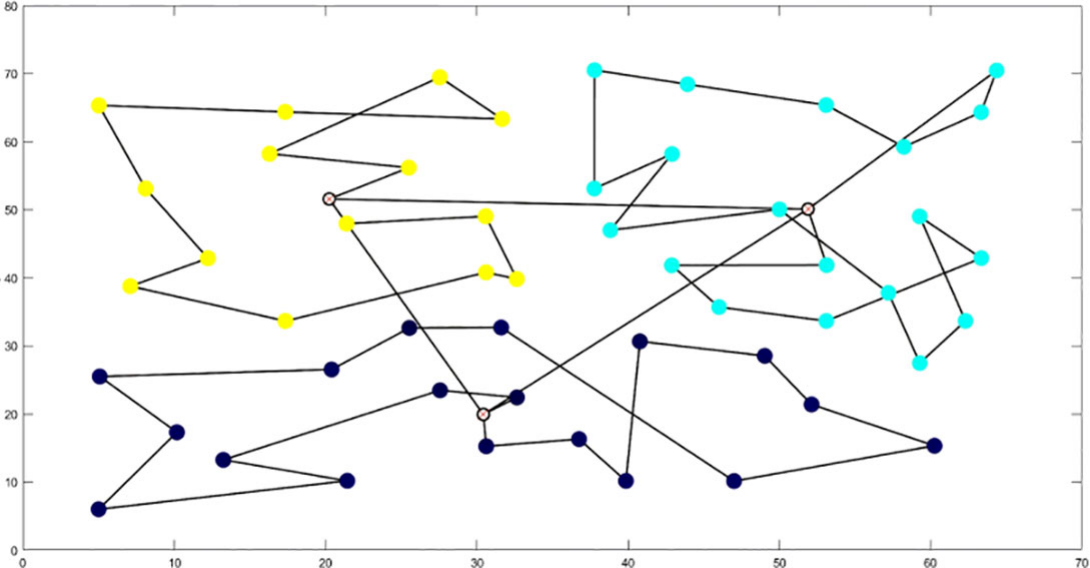 557.6	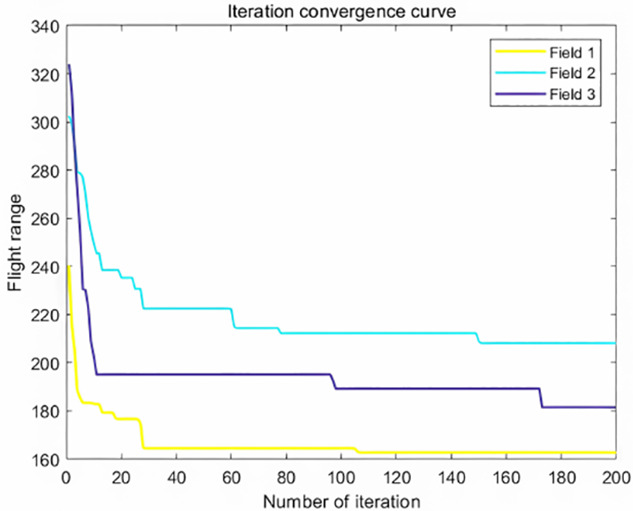 141.7
BSO	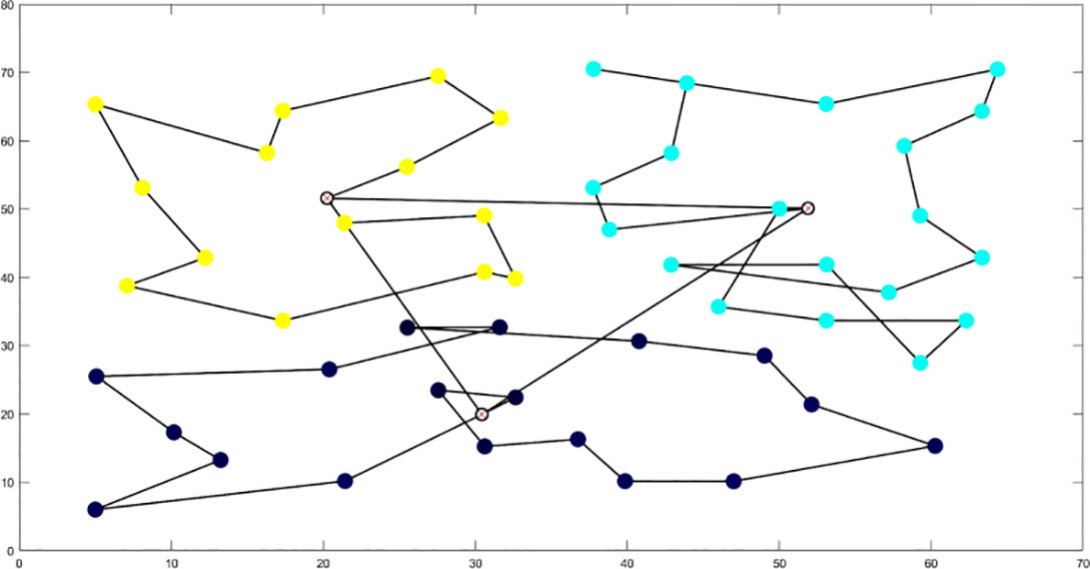 541.6	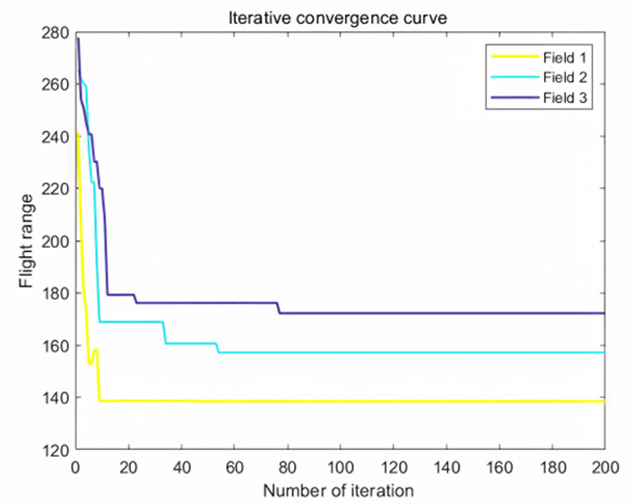 46.7
GA-ACO	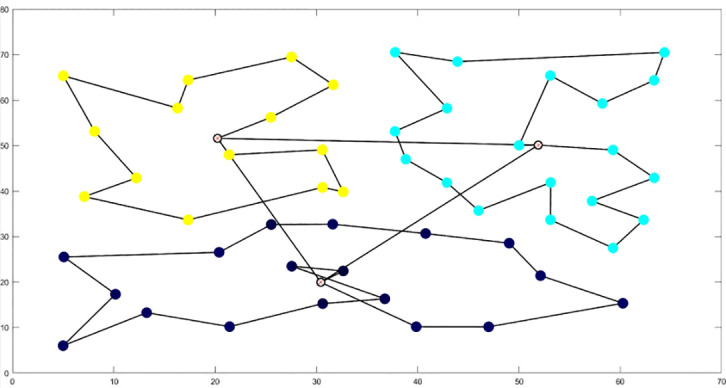 507.3	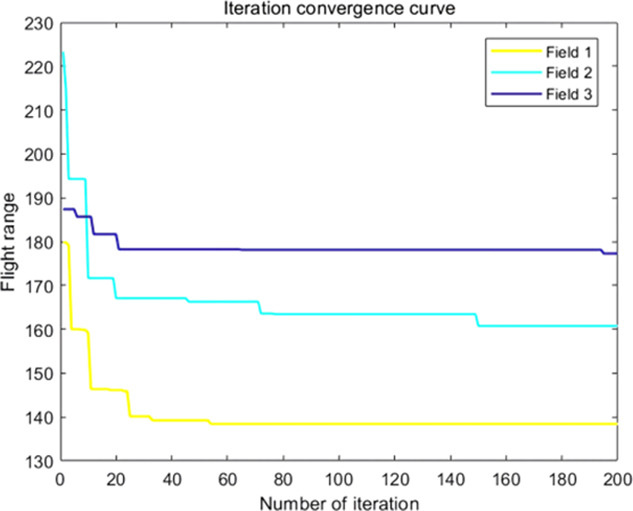 133.4
ACHAGA	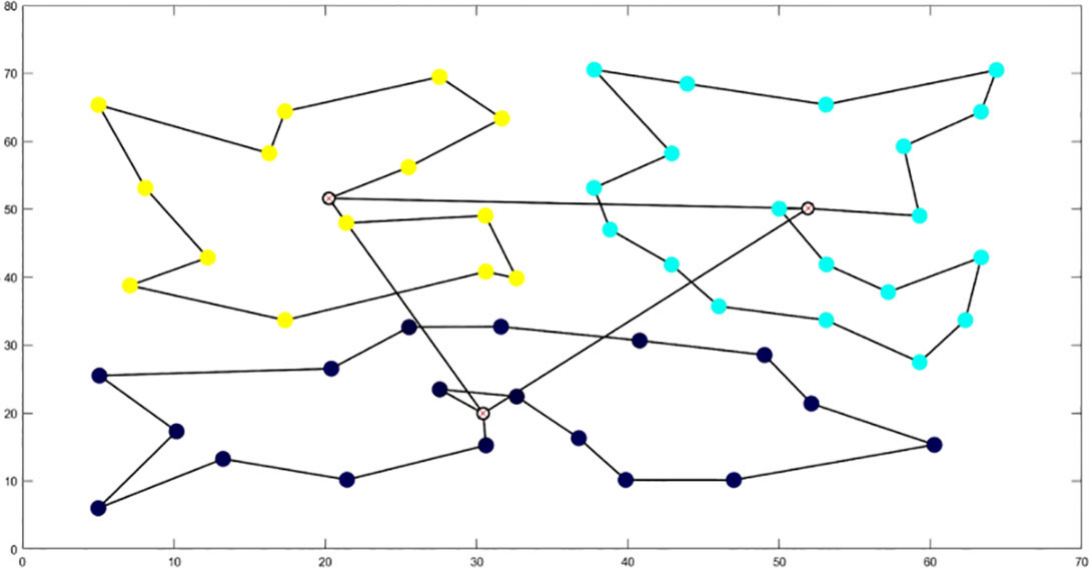 460.5	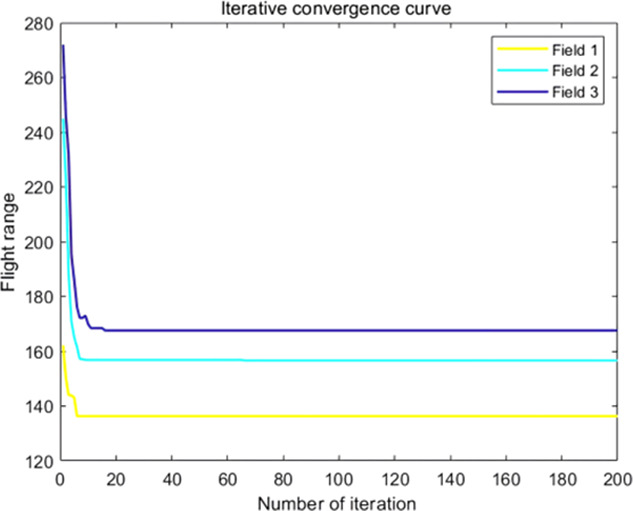 11.7
**GA**	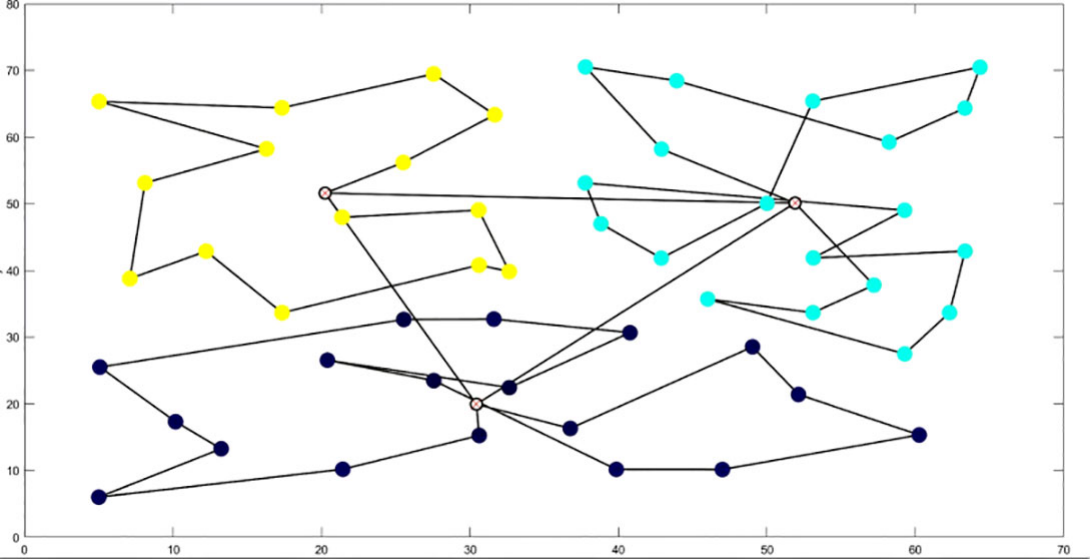 533.7	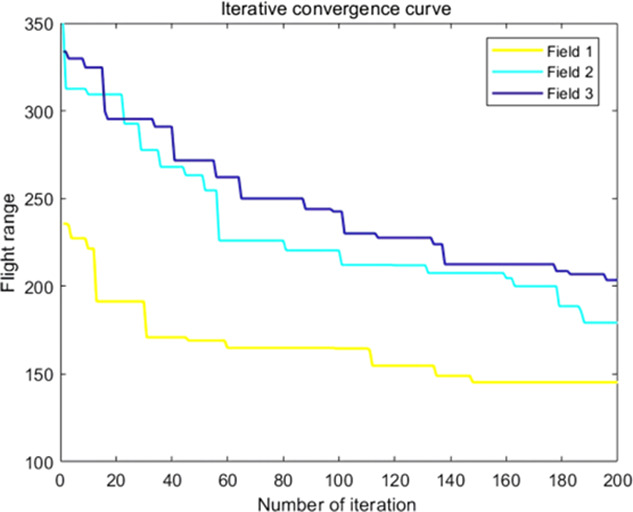 177.3

**Table 8:** Simulation outcomes for different multi-objective problems.

Problem	Algorithm	Average Value(m)	Range(m)	CV	Confidence Interval
Multi-tea Field Problem Model	GA	14598.09	1819.64	0.02797023	[14390.09, 14806.09]
	GA-ACO	10619.34	777.67	0.01629405	[10534.79,10703.89]
	AFSA	13497.23	585.71	0.01340668	[13430.37,13564.086]
	BSO	10493.38	358.70	0.00930587	[10444.34, 10542.42]
	ACHAGA	9693.27	56.29	0.00406241	[9677.34, 9709.20]
Eil51	GA	563.66	32.62	0.01904130	[560.17, 567.15]
	GA-ACO	512.34	11.07	0.00754431	[510.40, 514.28]
	AFSA	577.61	29.86	0.01807257	[572.86, 582.36]
	BSO	550.43	15.53	0.01157147	[546.52, 554.34]
	ACHAGA	462.26	4.56	0.00326200	[461.67, 462.85]

#### 4.1.3 Disparities in algorithmic optimization performance across various problem complexities

Analysis of **Table 8** indicates minor discrepancies in the simulation outcomes for the standard eil51 problem across all algorithms. However, when addressing the multi-tea field problem model with expanded solution spaces, AFSA and GA, which typically yield superior results, exhibit a marked decline in performance. Conversely, other algorithms demonstrate less variability in solution quality across different problems but still show significant divergence from ACHAGA’s outcomes. This variation is likely attributable to the distinct mechanisms and coding methodologies of each algorithm, which may limit their ability to derive reasonable results within a finite number of iterations. The p-values in **Table 9**, being under 5%, substantiate the enhanced performance resulting from the optimization research conducted in this study. ACHAGA has proven its efficacy in identifying optimal solutions for the problem models within 200 iterations, showcasing robust adaptability suitable for more intricate scenarios. The Pearson correlation coefficients in **Table 9** provide insight into the relationship between the performance metrics of ACHAGA and the other algorithms. For the multi-tea field problem and eil51, the coefficients are generally low, with both positive and negative values, indicating weak correlations. This suggests that the performance patterns of ACHAGA are somewhat independent of those of the other algorithms. For eil51, the coefficient variance is relatively greater. The negative correlations with GA and GA-ACO suggest that as ACHAGA’s performance improves, the performance of these algorithms tends to worsen, and vice versa. The results demonstrate that ACHAGA significantly outperforms the other algorithms across both problem instances. The statistical significance of the p-values confirms the robustness of ACHAGA’s performance improvements. The Pearson correlation coefficients further illustrate the nature of these performance differences, highlighting the unique strengths of ACHAGA in addressing the complexities of both the multi-tea field problem and eil51.

**Table 9:** Comparison of significance level results between ACHAGA and each algorithm.

Comparison of algorithms	*p*-Value		*PCCs*	
	Multi-tea Field Problem	Eil51	Multi-tea Field Problem	Eil51
ACHAGA *vs*.GA	2.24E-37	1.47E-14	-0.015273	-0.513957
ACHAGA *vs*.GA-ACO	3.94E-24	1.67E-12	0.374056	-0.150358
ACHAGA *vs*.BSO	4.77E-15	3.26E-14	-0.275725	0.057610
ACHAGA *vs*.AFSA	3.09E-46	8.49E-17	-0.118178	0.386316

#### 4.1.4 Comparative analysis of the proposed algorithm with other algorithms

The performance analysis of ACHAGA indicates that it surpasses traditional heuristic algorithms in several key performance indicators. To understand why, we examine the principles and characteristics of various algorithms. GA, which relies on random mutations and crossover, often faces issues with slow convergence and premature convergence. ACHAGA addresses these issues with its hybrid GA-Simulated Annealing (SA) approach, combining GA’s global search capabilities with SA’s local precision for faster convergence. The Genetic ACO Fusion Algorithm, while enhancing exploration by integrating GA and Ant Colony Optimization (ACO), suffers from higher iteration counts and complex parameter control. ACHAGA’s adaptive crossover and mutation rates dynamically adjust based on fitness, ensuring efficient convergence with fewer iterations. AFSA’s performance is hindered by sensitivity to parameter settings and slower convergence in complex spaces. ACHAGA’s adaptive Metropolis criterion, which incorporates temperature and solution space fluctuations, balances exploration and exploitation effectively, preventing premature convergence. BSO, which mimics brainstorming, generates diverse solutions but shows higher variance and slower convergence. ACHAGA’s efficient cooling strategy, using logarithmic and exponential functions, enhances convergence speed and accuracy, avoiding local minima and ensuring robust optimization. These optimization features enable ACHAGA to achieve superior performance with shorter optimal solution lengths, fewer iterations, and higher stability, making it an efficient solution for complex optimization problems in a multi-tea field environment.

### 4.2 Field trial results analysis

**Table 4** delineates the full-coverage and scheduling routes devised by three methodologies. The algorithm from this study yields more evenly distributed routes with reduced crossover, mitigating potential UAV interference. A comparison between the theoretical and actual flight paths reveals deviations due to factors such as wind speed, terrain gradient, and positioning stability, with the actual flight distance exceeding theoretical estimates. **Table 5** enumerates the total real flight, operational, and dispatch ranges planned by the three methods, highlighting the superiority of this study’s algorithm in these metrics and its ability to effectively minimize operational and dispatch distances. Specifically, compared to manual empirical and brainstorming methods, this algorithm decreases the actual flight’s operational distance by 791.9 meters and 359.6 meters, respectively, and reduces the non-operational time share by 4.2% and 6.7%. Notably, the scheduling distance is nearly halved on average, suggesting that the clustering fusion planning algorithm substantially curtails UAVs’ non-operational flights. Moreover, full-coverage planning utilizing heuristic algorithms reduces the operational distance by an average of 11.4% and the number of turns by 11, compared to manual empirical planning. This demonstrates its superior applicability for multi-terrain field route scheduling in the hilly, mountainous environment examined in this study.

### 4.3 Research limitations

Despite the considerable advancements of the ACHAGA framework in UAV-based precision plant protection, several limitations warrant attention. Firstly, potential biases inherent in simulation studies may undermine the applicability of the findings to real-world scenarios, as the simulations did not account for unforeseen weather changes, equipment malfunctions, or human interventions. Secondly, the scope of field tests was geographically constrained and specific to certain types of tea fields, potentially limiting the generalizability of the results to other terrains and crop types. To address these limitations, we need to expand field tests to encompass a broader range of geographic regions and crop varieties to enhance the robustness of the algorithm’s performance. Additionally, developing methods for dynamic resupply point management based on real-time data could further optimize UAV operations. Integrating advanced sensing technologies, such as LiDAR and multispectral imaging, could significantly improve terrain mapping and environmental monitoring, thereby refining route planning precision. Addressing these limitations through rigorous real-world testing and technological enhancements will be crucial for the broader adoption and efficacy of the ACHAGA framework in diverse agricultural contexts.

## 5 Conclusion

This study introduces the Adaptive Clustering Hyperbolic Annealing Genetic Algorithm (ACHAGA), a hybrid optimization algorithm that integrates the principles of hyperbolic genetic algorithms and simulated annealing. The primary objective is to enhance the precision of UAV-based plant protection scheduling in complex multi-terrain environments. The algorithm leverages efficient crossover and mutation operations inherent to hyperbolic genetic algorithms to achieve rapid convergence and maintain spatial diversity. High-quality solutions derived from this process serve as initial inputs for the simulated annealing algorithm, which, through its jump mutation properties and innovative temperature control mechanism, further refines solution quality and robustness. By employing cluster analysis, multi-tea field regions are segmented, and operational supply centers and flight schedules are meticulously designed to ensure UAVs adhere to the shortest feasible routes within their endurance limits in designated plant protection areas. ACHAGA demonstrates superior performance in both standard traveler’s problem scenarios and multi-tea field applications, swiftly identifying optimal solutions and exhibiting robust global search capabilities and high stability. Future research should prioritize incorporating real-time environmental data, such as weather conditions, soil moisture levels, and pest distribution patterns, to refine route planning further. Evaluating the algorithm’s scalability and adaptability across robustness crop types and terrains, and developing strategies to optimize UAV battery usage and recharge cycles for more sustainable operations, is also crucial. These research directions aim to build on the current study’s foundation, advancing the capabilities of UAV-based precision agriculture. This work establishes a theoretical and technical foundation for the efficient management and operation of extensive tea plantations in hilly and mountainous regions, offering new perspectives on precision planning and management of UAV plant protection operations in complex settings. Beyond the immediate scope of this study, the findings have broader implications for the field of agricultural technology. The integration of real-time environmental data into UAV route planning could lead to more responsive and adaptive agricultural management systems, capable of adjusting to changing conditions such as sudden weather changes or pest outbreaks. This adaptability is crucial for improving crop yields and reducing losses, thereby supporting food security initiatives. The scalability of the ACHAGA algorithm also suggests its potential use in larger and more diverse agricultural settings.

## Data Availability

The original contributions presented in the study are included in the article/**Supplementary Material**. Further inquiries can be directed to the corresponding authors.
