# Supplementary figures and images for: An integrated framework for UAV-based precision plant protection in complex terrain: the ACHAGA solution for multi-tea fields
Source: Front Plant Sci. 2024 Sep 26;15:1440234. doi: 10.3389/fpls.2024.1440234 (PMC11464358; doi:10.3389/fpls.2024.1440234)

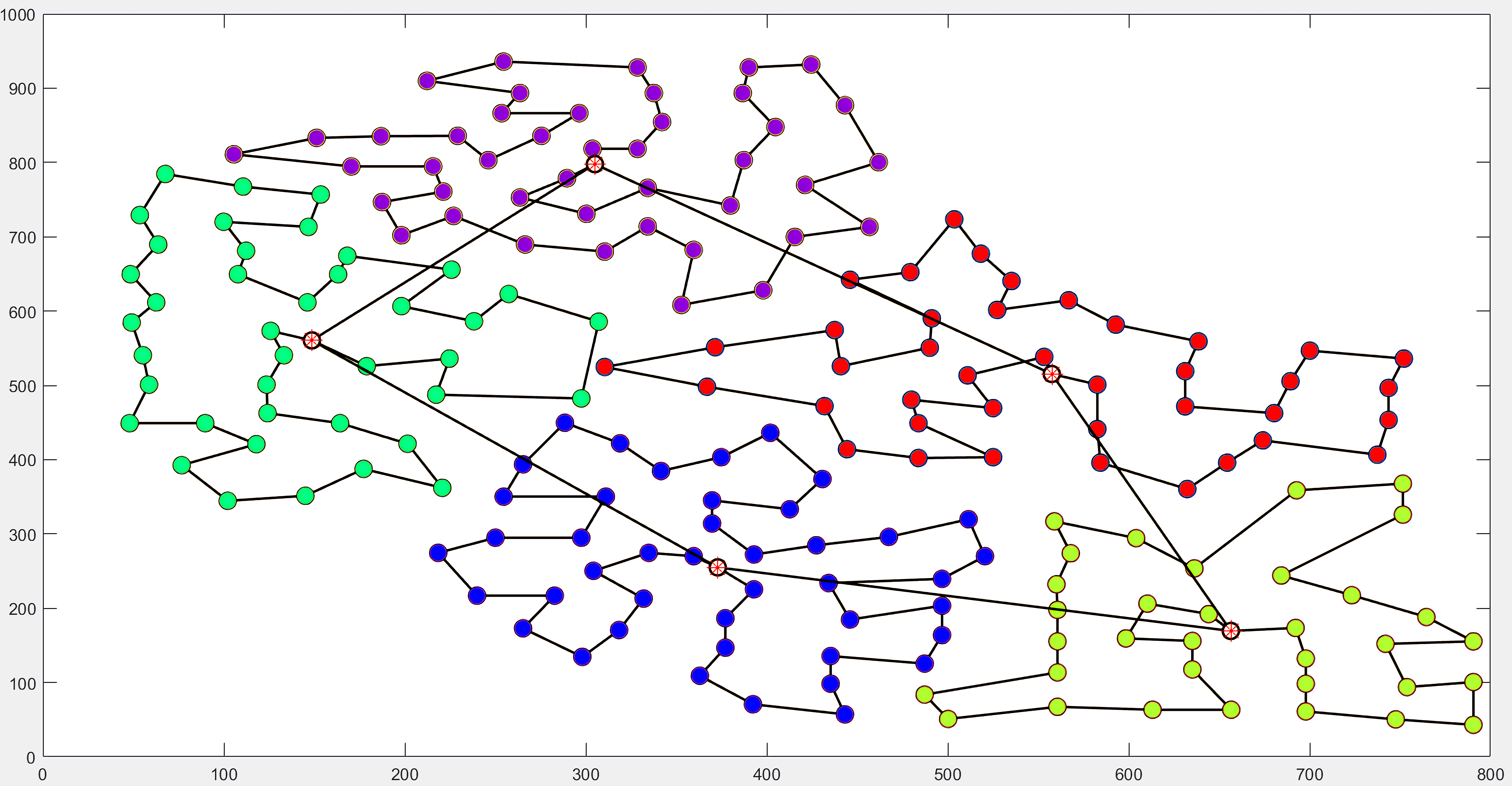

Supplement: Supplementary file 1 [file DataSheet1.zip › Table 4/ACHAGA(left).jpg]

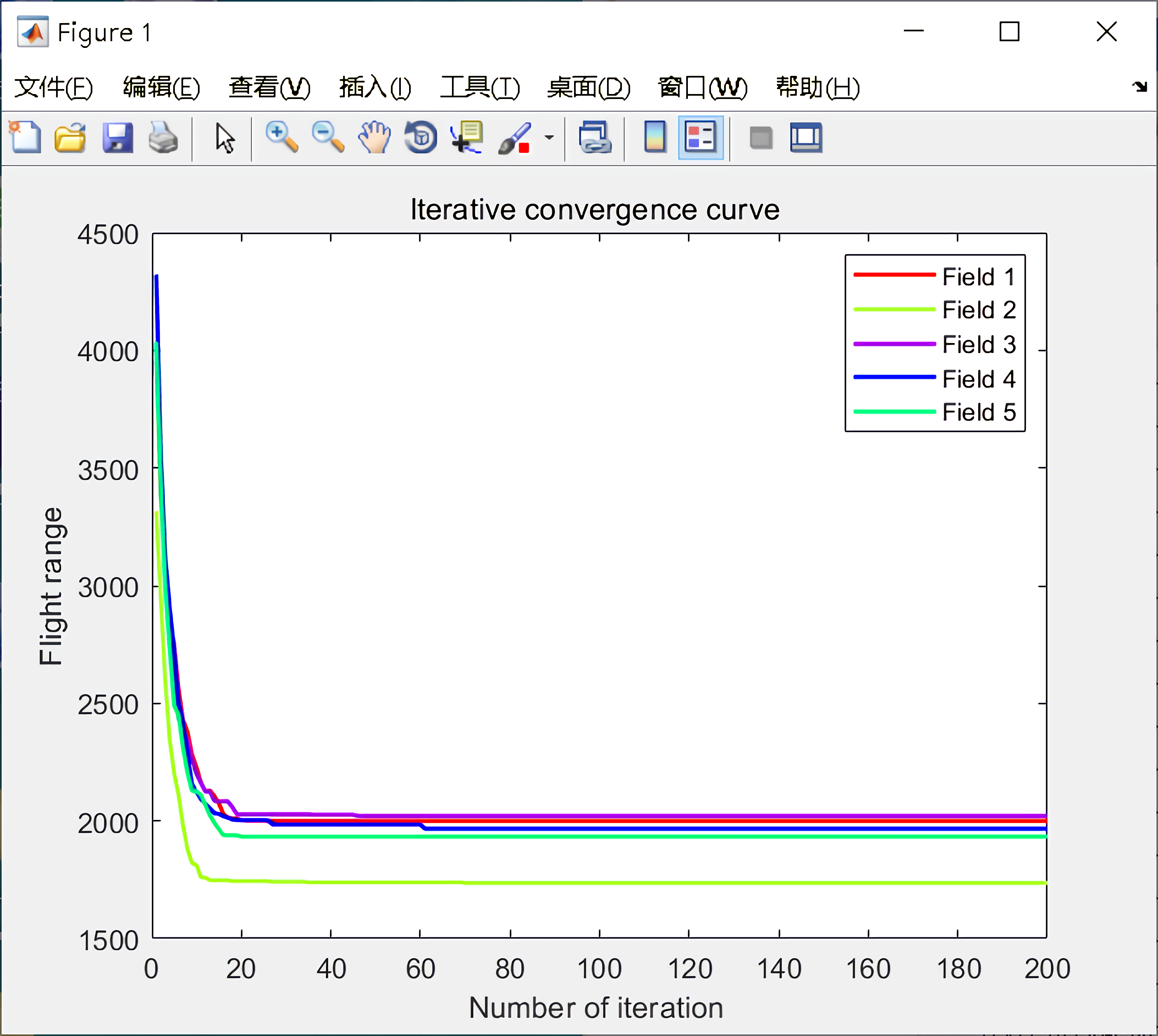

Supplement: Supplementary file 1 [file DataSheet1.zip › Table 4/ACHAGA(right).jpg]

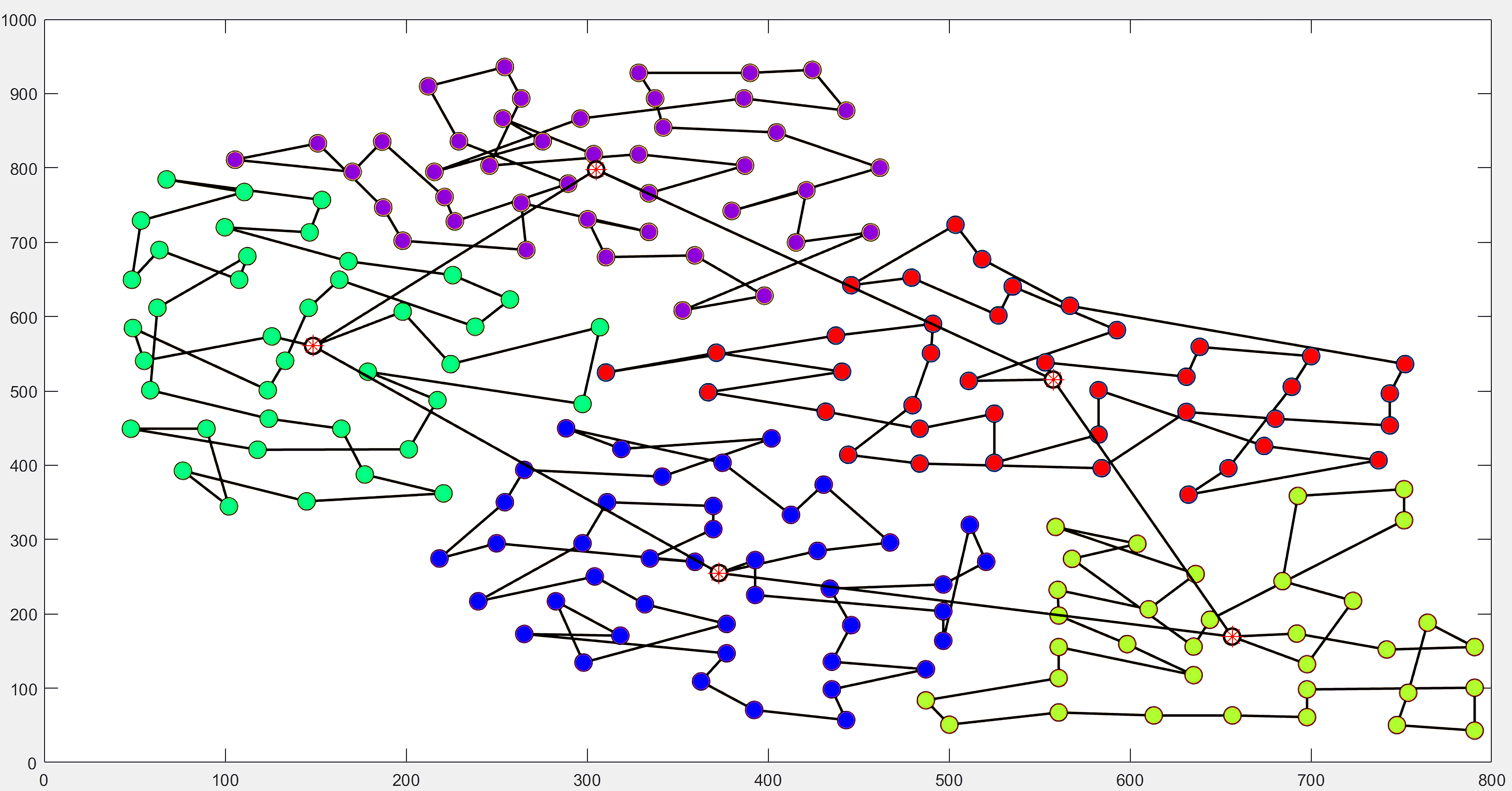

Supplement: Supplementary file 1 [file DataSheet1.zip › Table 4/AFSA(left).jpg]

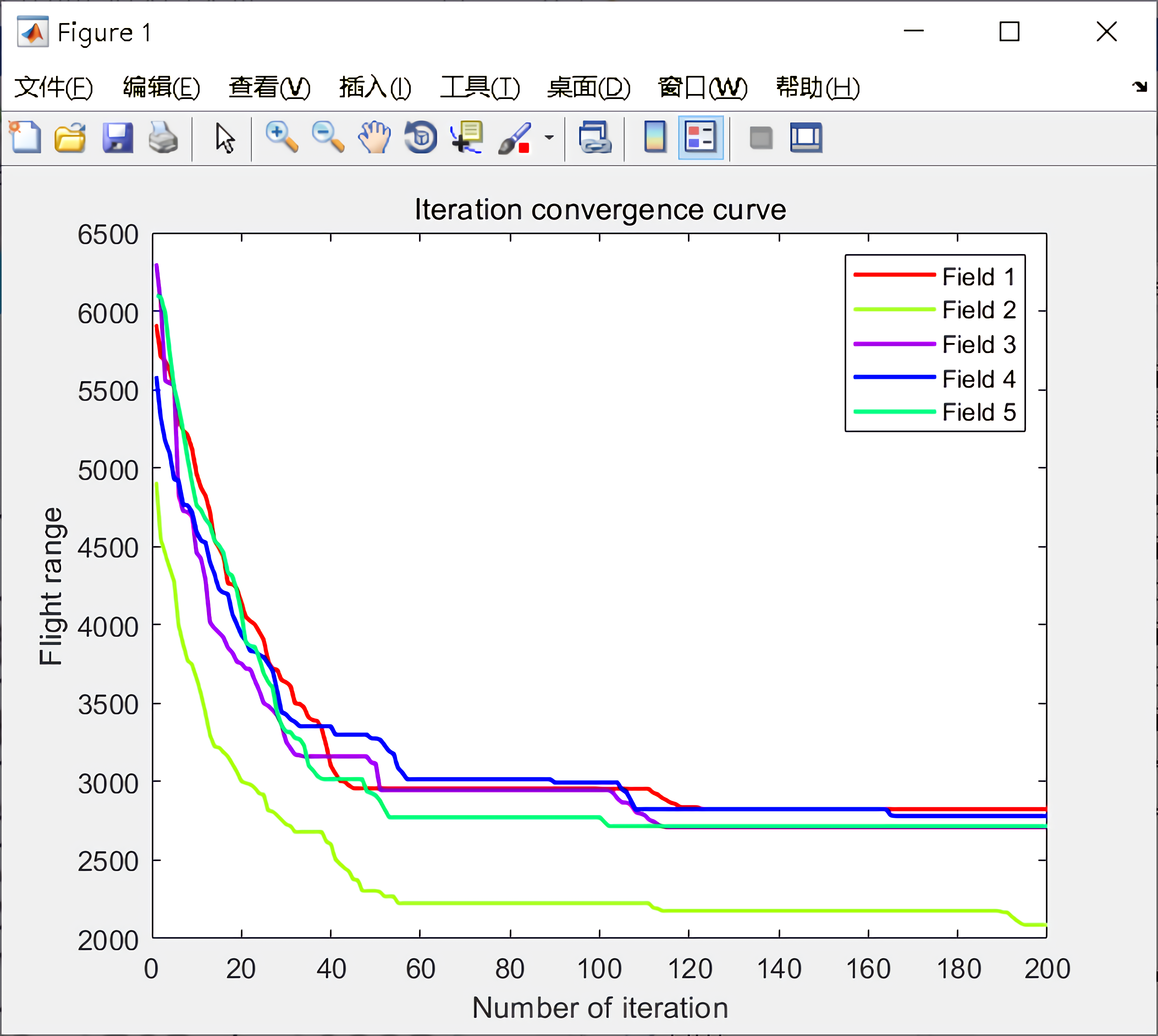

Supplement: Supplementary file 1 [file DataSheet1.zip › Table 4/AFSA(right).jpg]

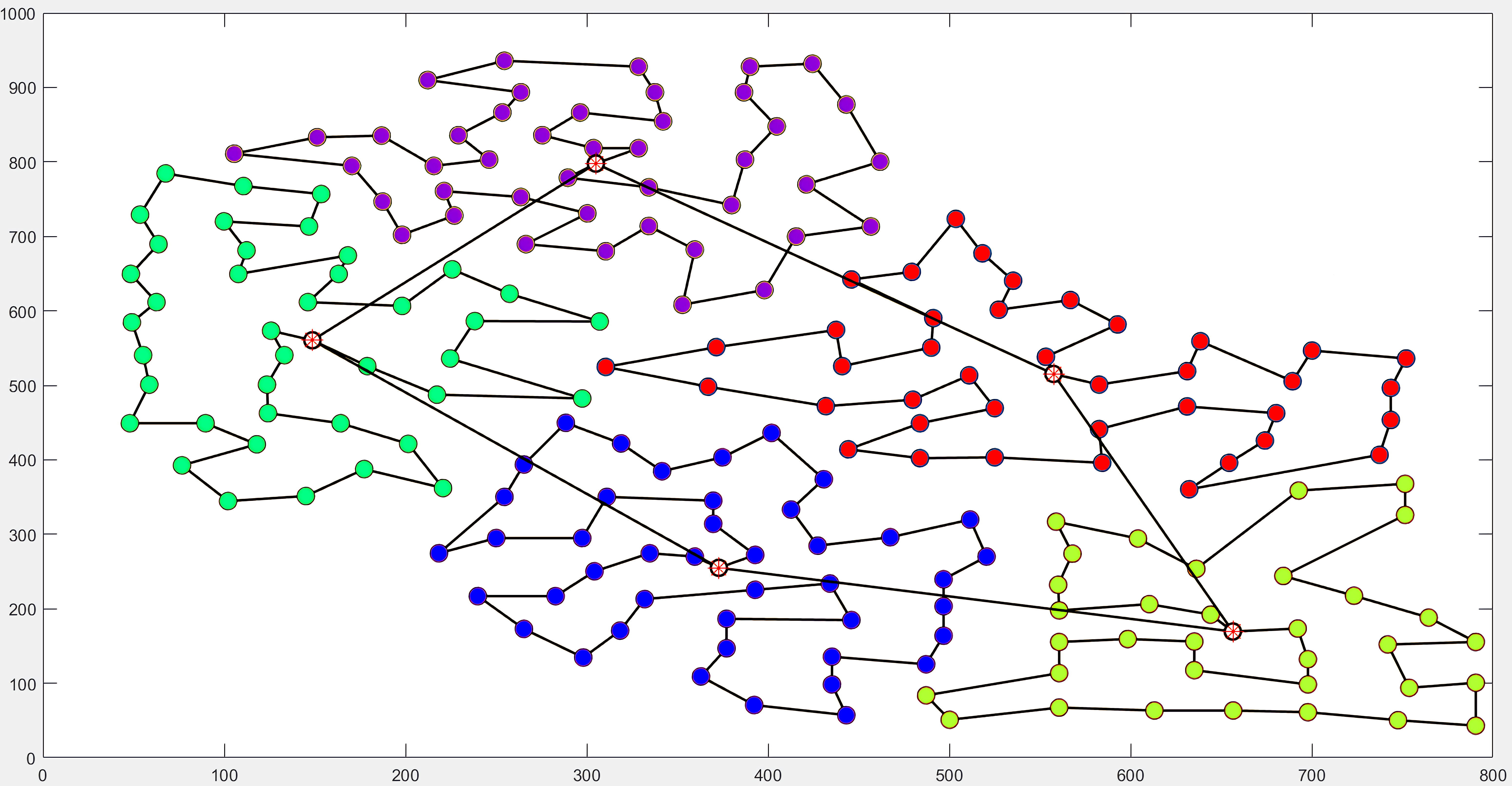

Supplement: Supplementary file 1 [file DataSheet1.zip › Table 4/BSO(left).jpg]

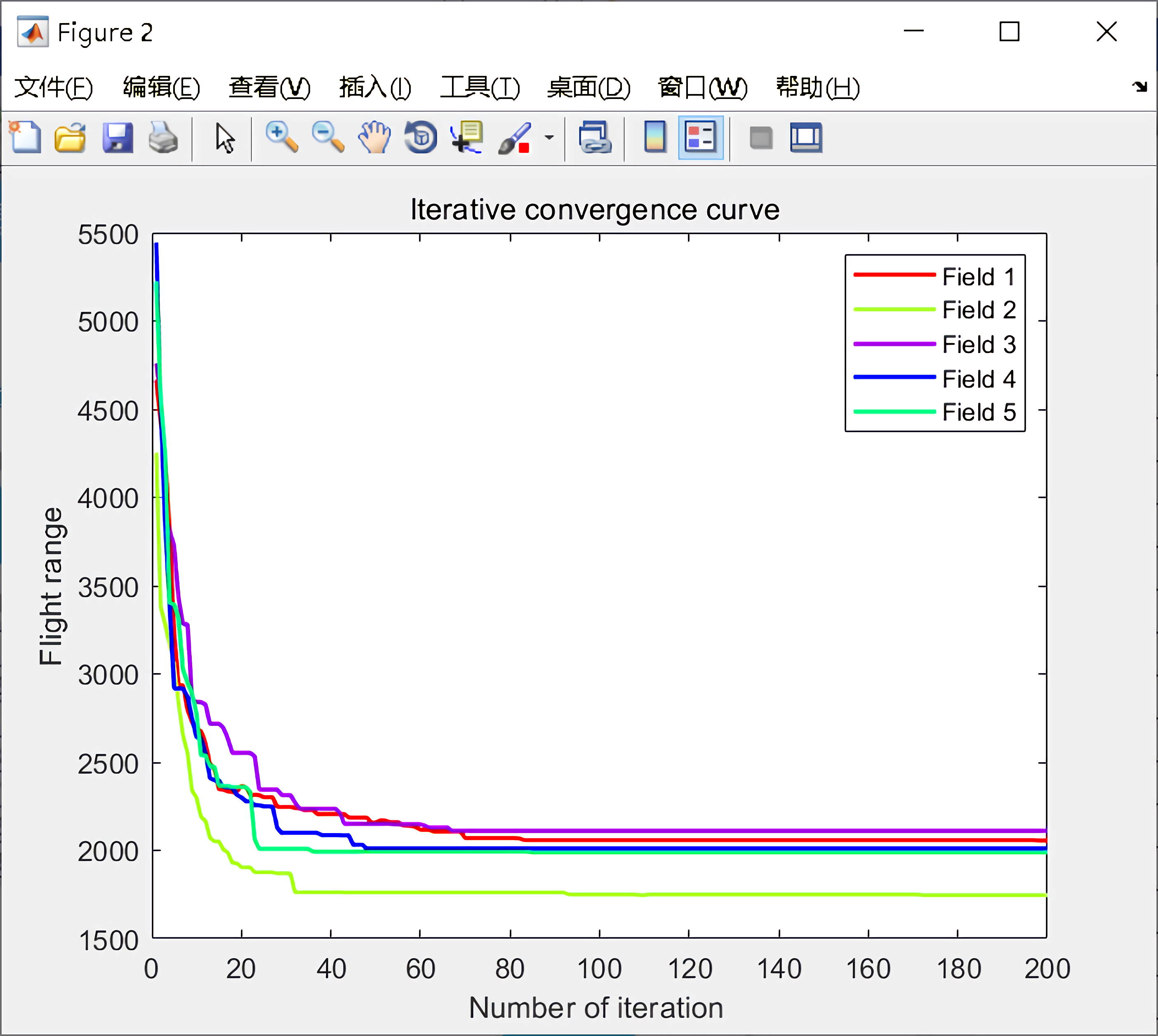

Supplement: Supplementary file 1 [file DataSheet1.zip › Table 4/BSO(right).jpg]

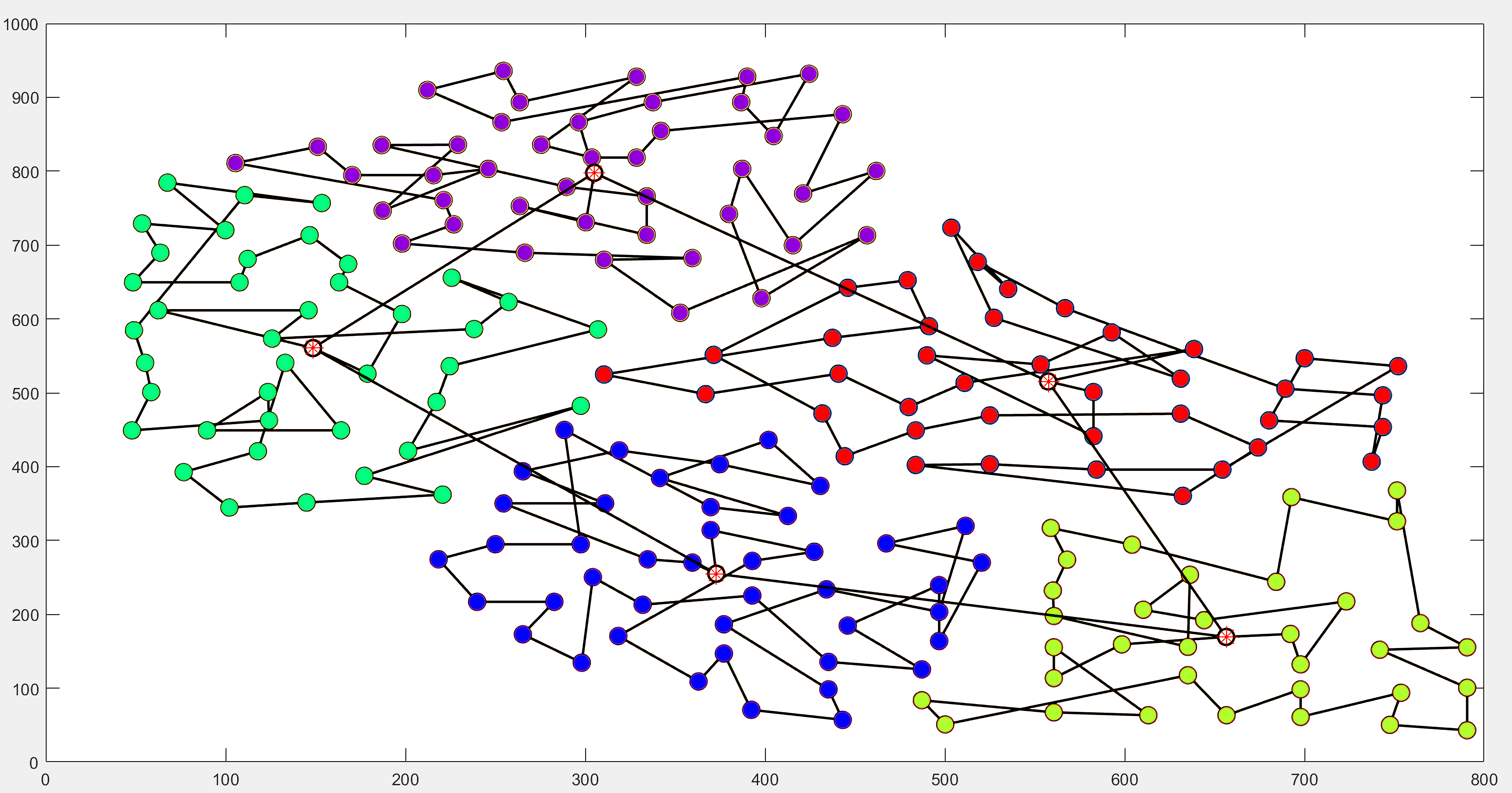

Supplement: Supplementary file 1 [file DataSheet1.zip › Table 4/GA(left).jpg]

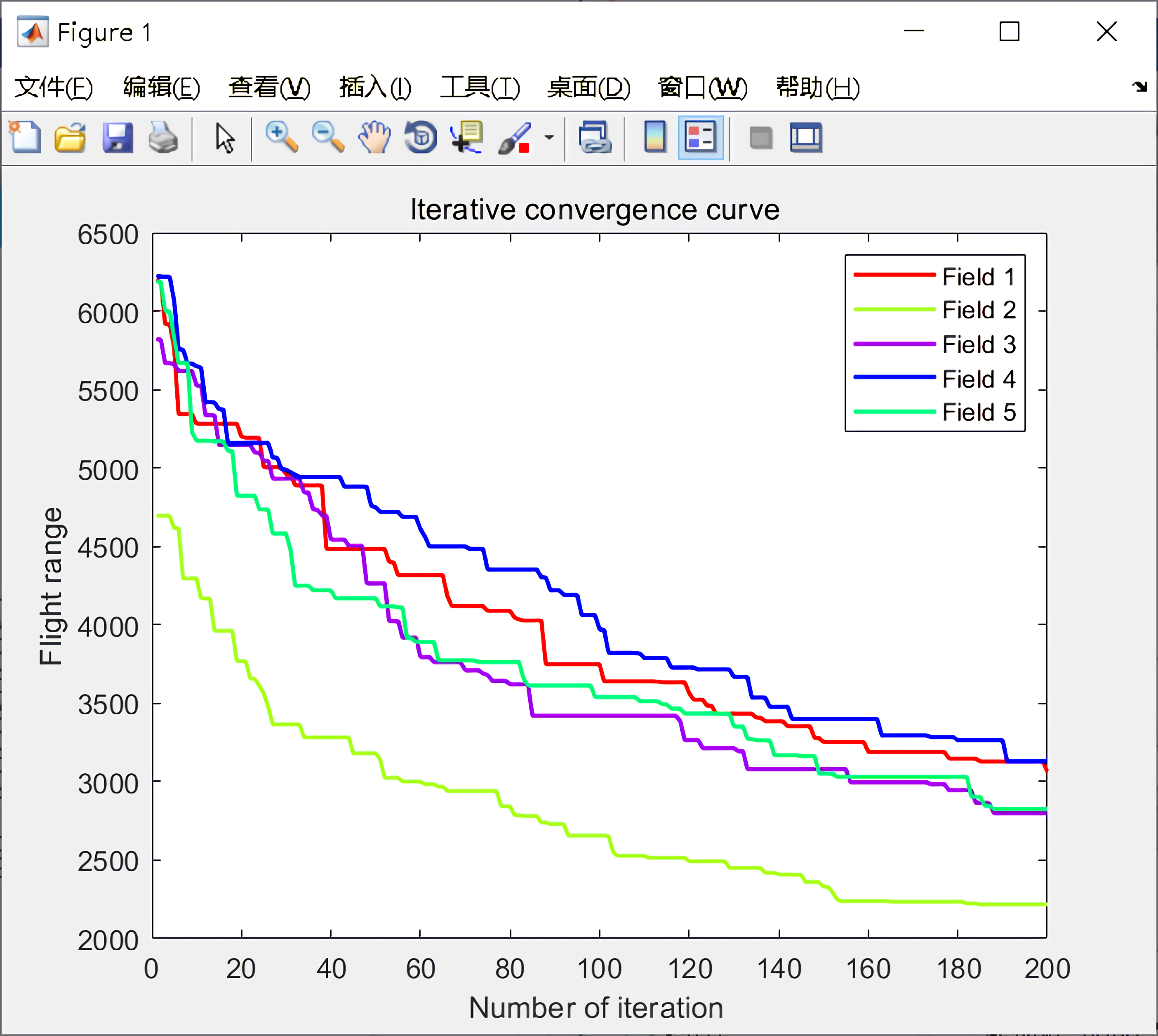

Supplement: Supplementary file 1 [file DataSheet1.zip › Table 4/GA(right).jpg]

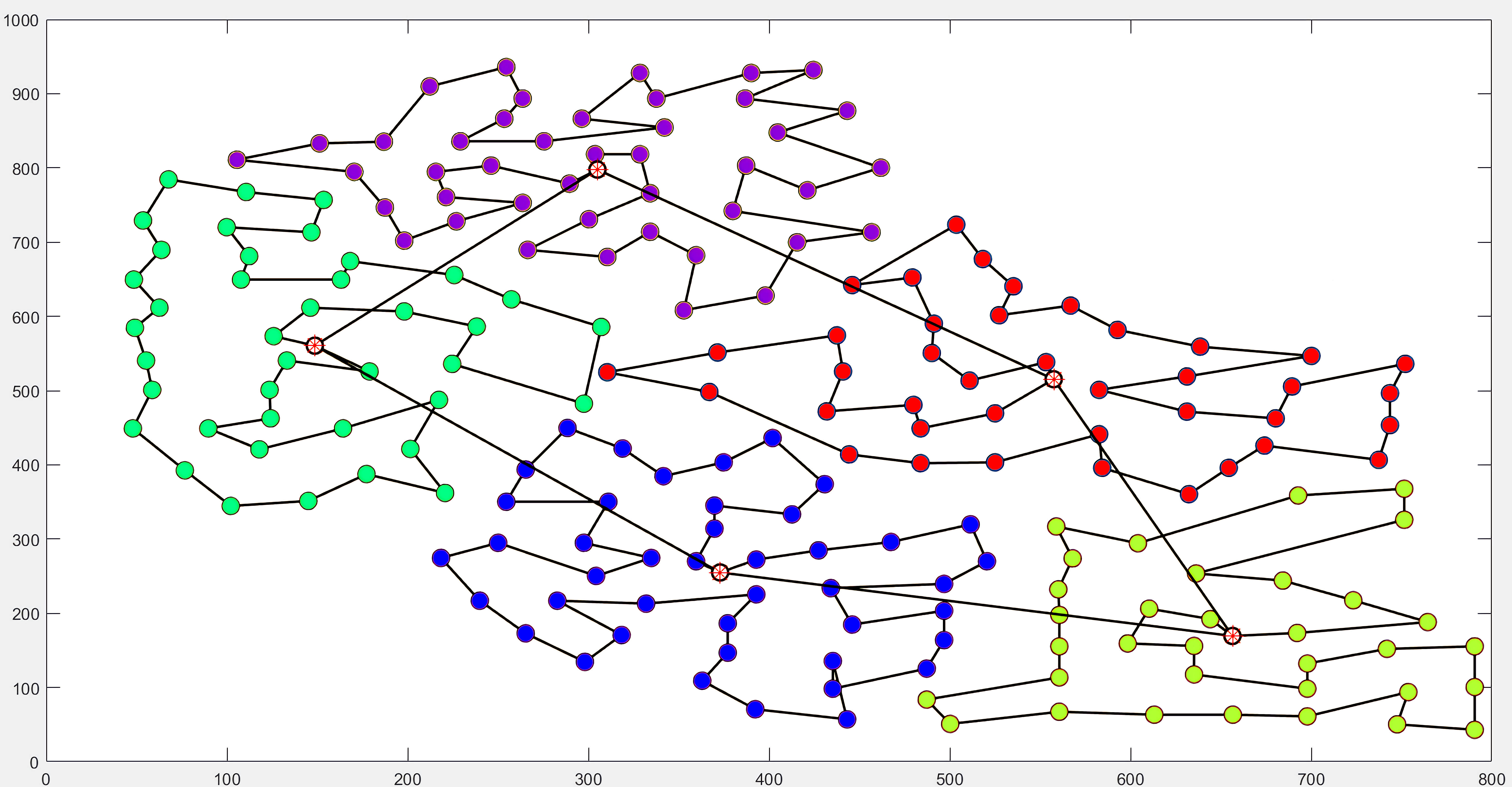

Supplement: Supplementary file 1 [file DataSheet1.zip › Table 4/GA-ACO(left).jpg]

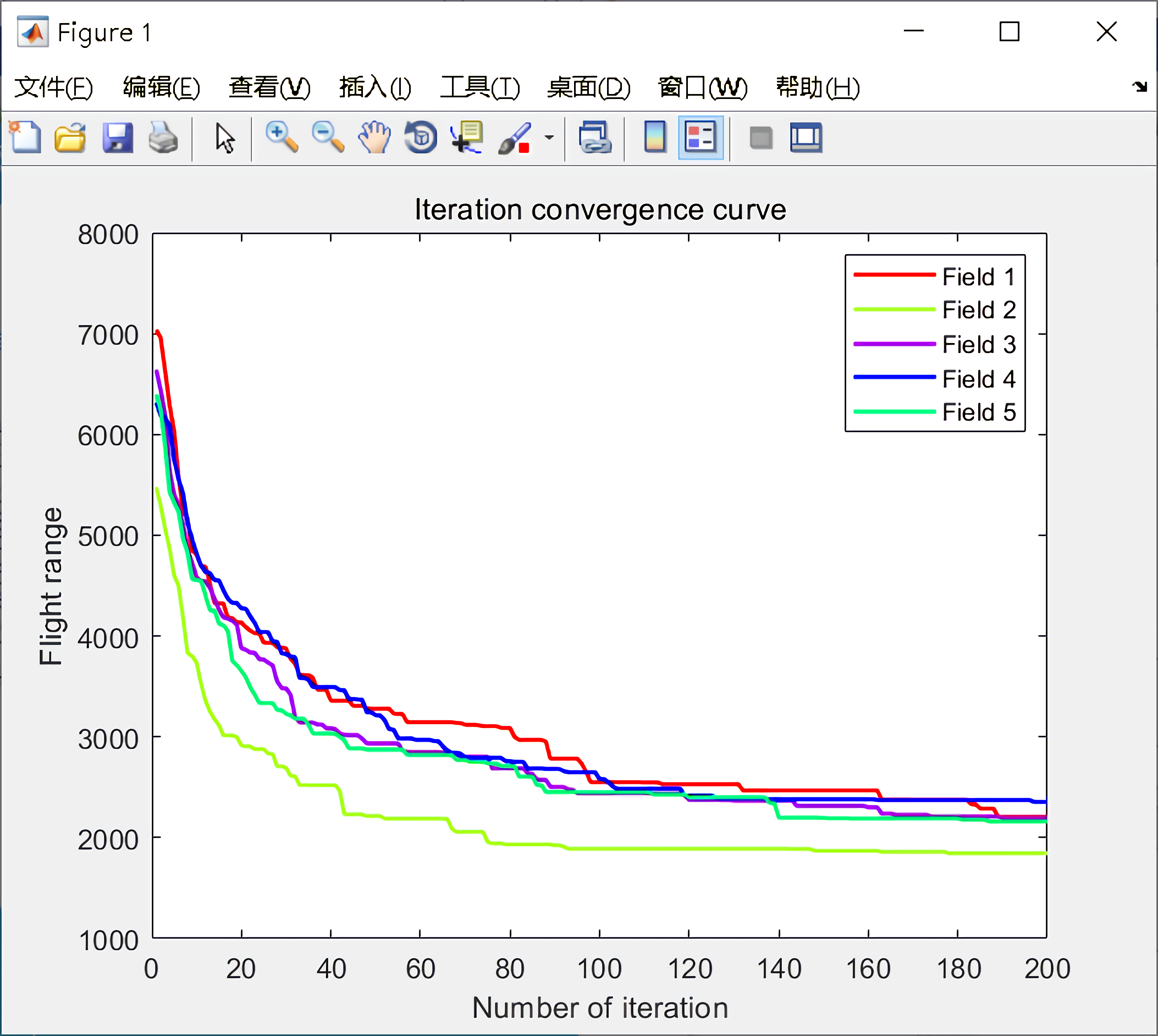

Supplement: Supplementary file 1 [file DataSheet1.zip › Table 4/GA-ACO(right).jpg]

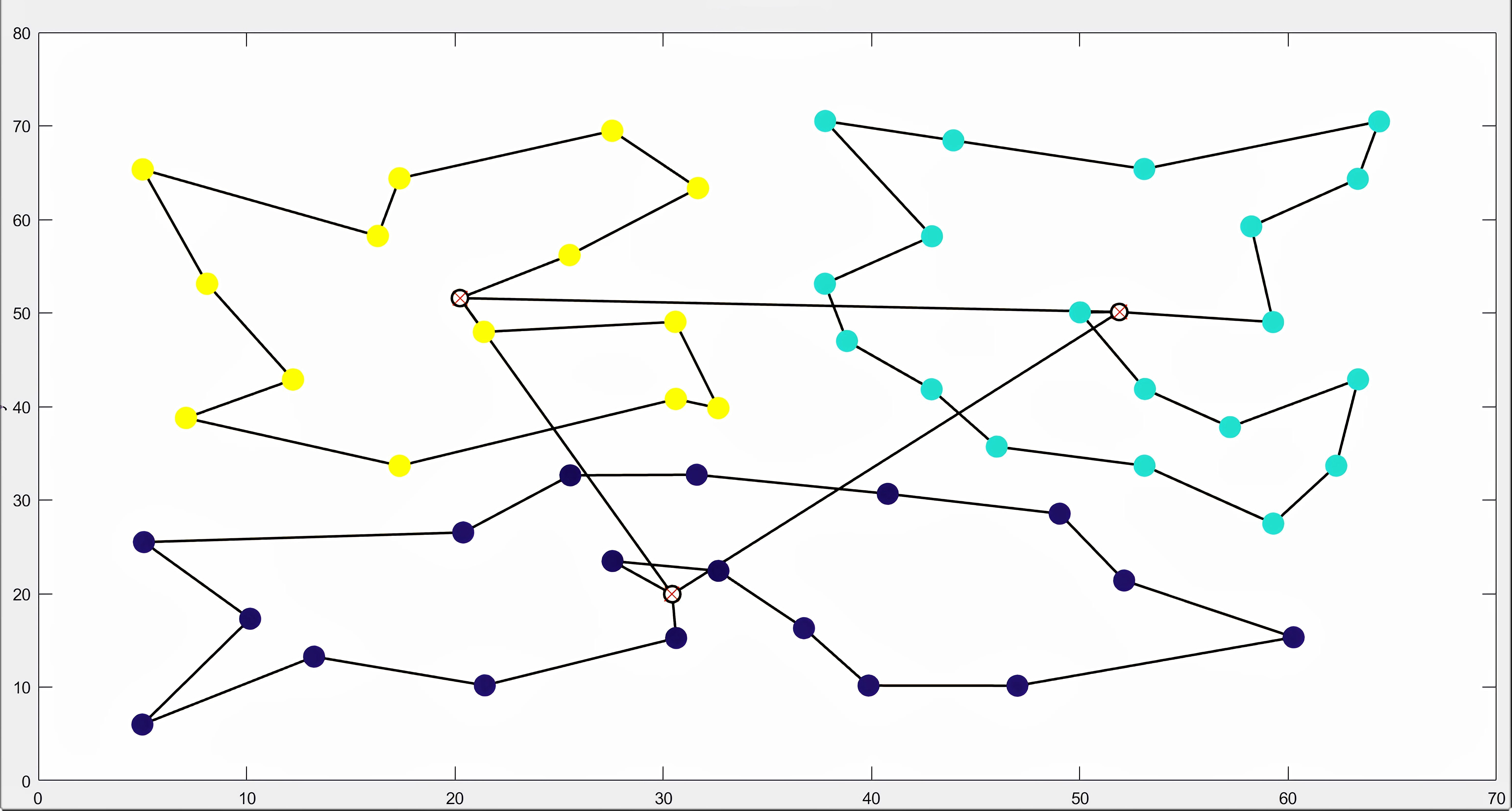

Supplement: Supplementary file 2 [file DataSheet2.zip › Table 5/ACHAGA(left).jpg]

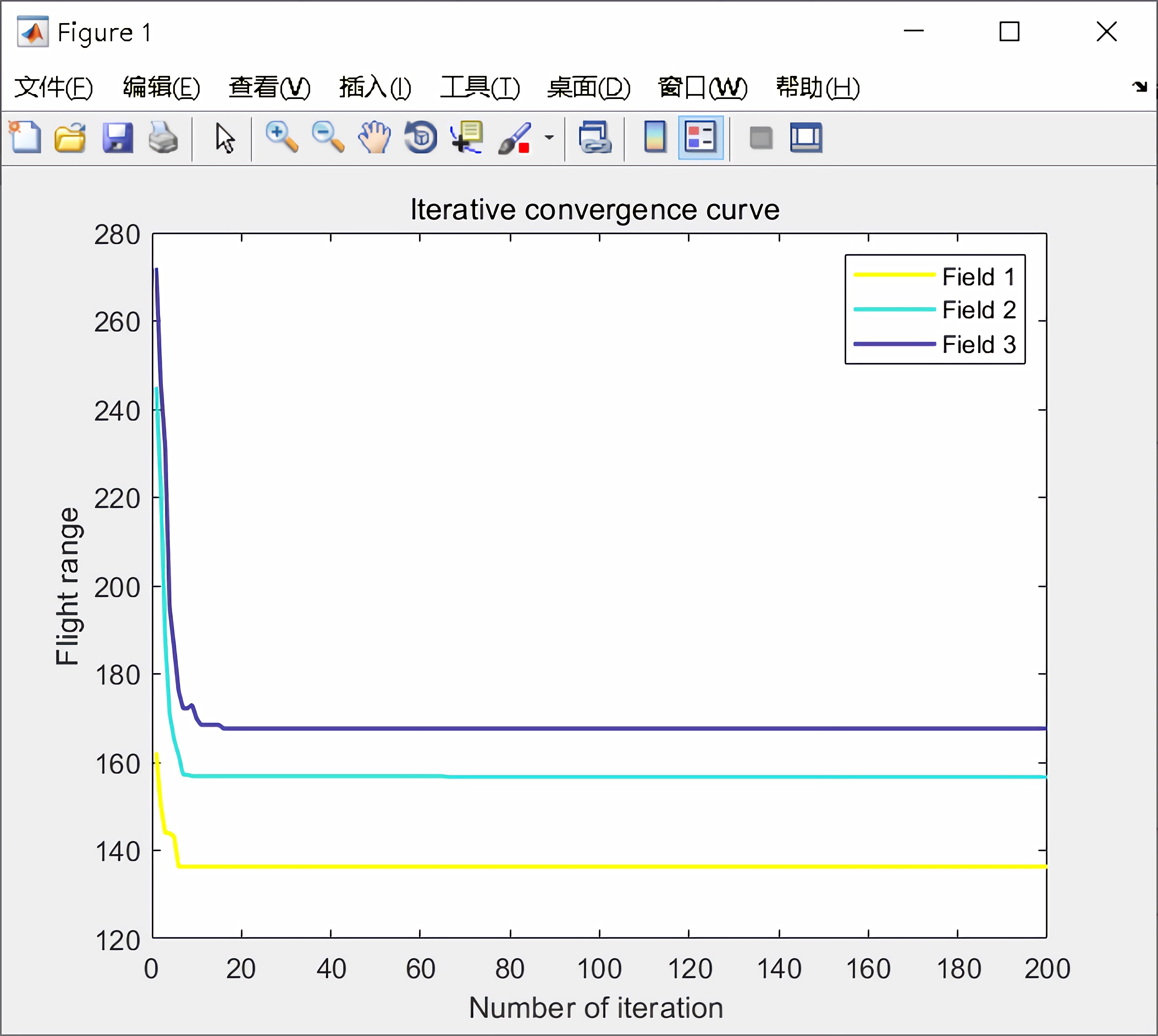

Supplement: Supplementary file 2 [file DataSheet2.zip › Table 5/ACHAGA(right).jpg]

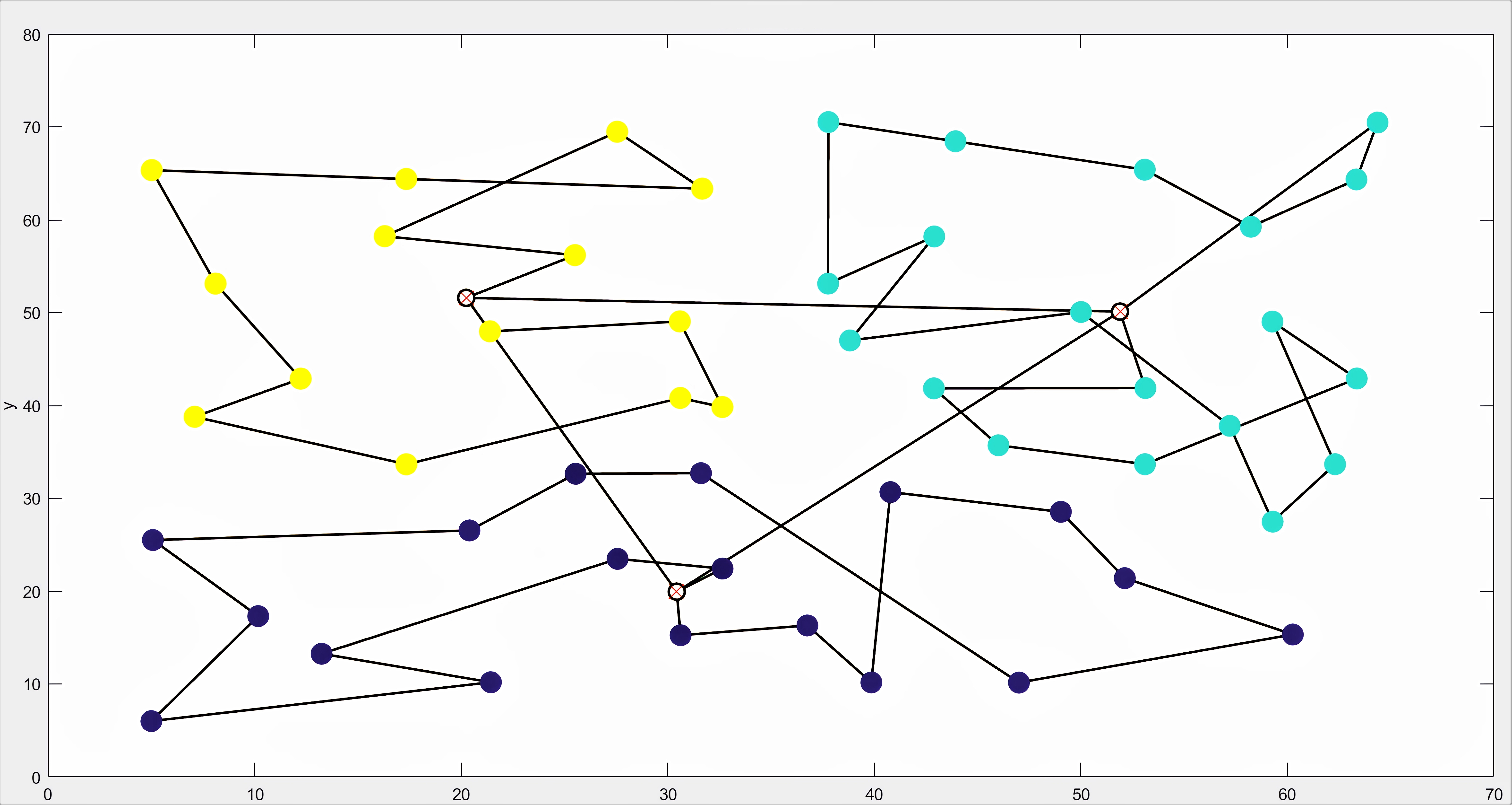

Supplement: Supplementary file 2 [file DataSheet2.zip › Table 5/AFSA(left).jpg]

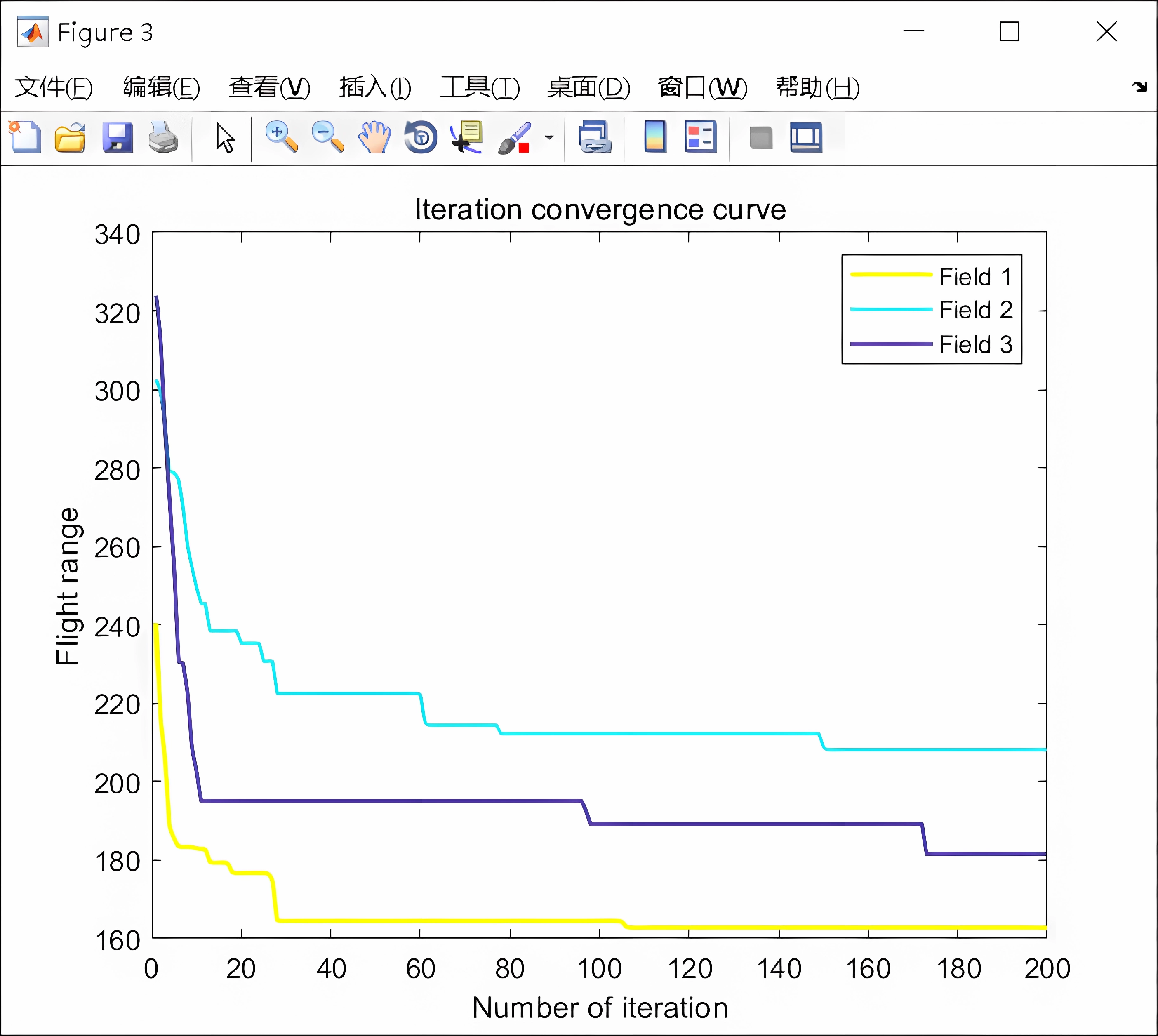

Supplement: Supplementary file 2 [file DataSheet2.zip › Table 5/AFSA(right).jpg]

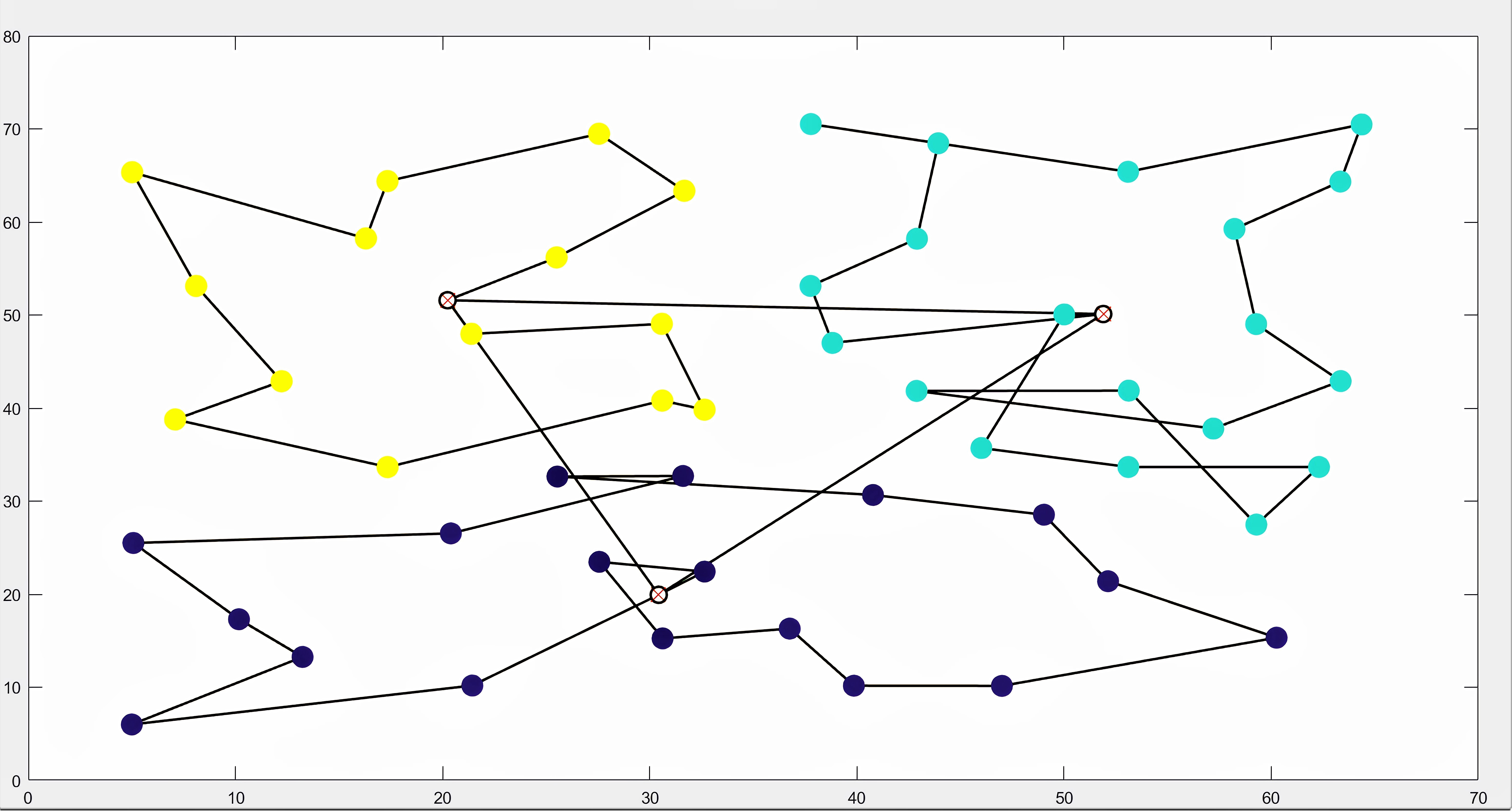

Supplement: Supplementary file 2 [file DataSheet2.zip › Table 5/BSO(left).jpg]

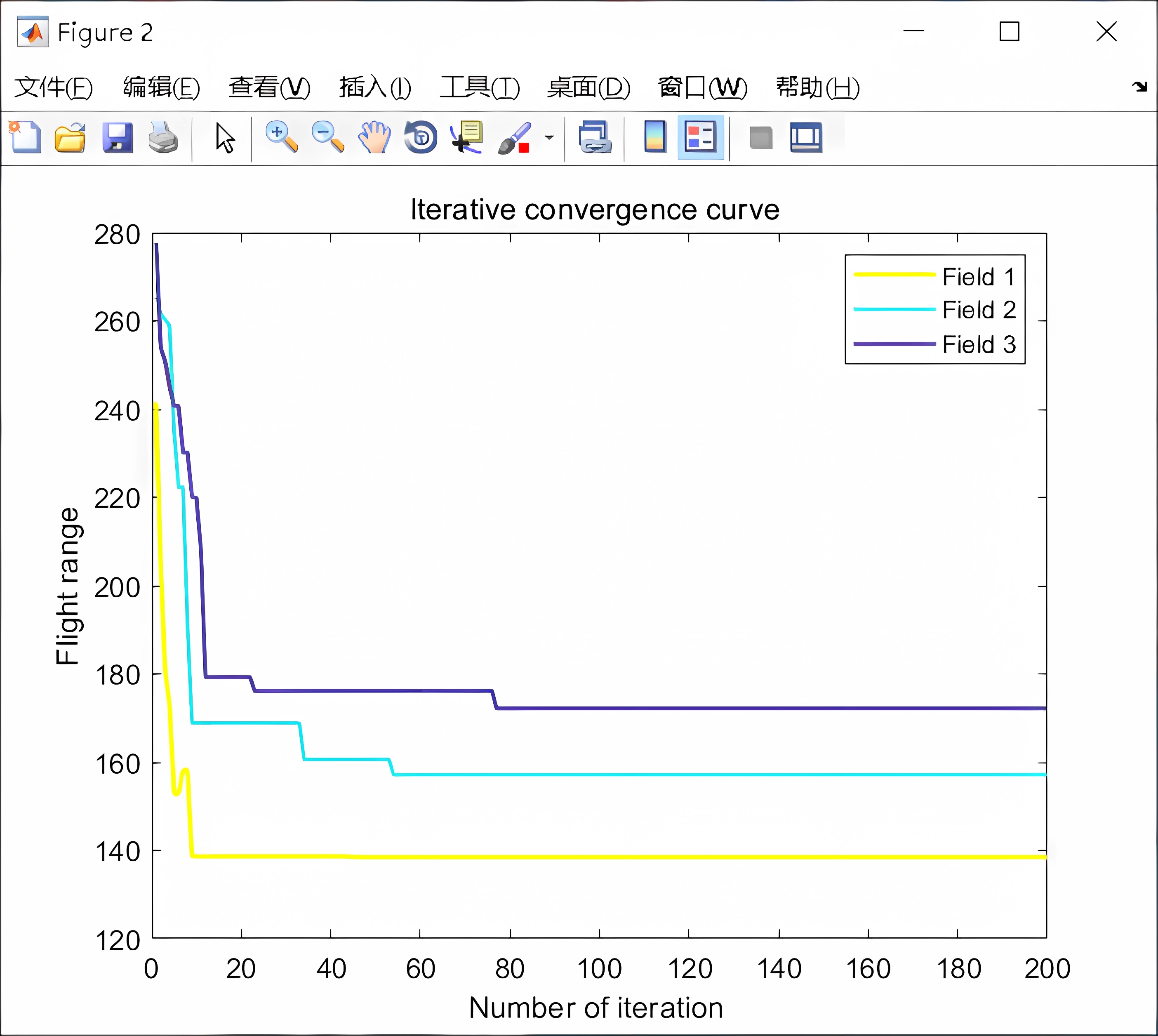

Supplement: Supplementary file 2 [file DataSheet2.zip › Table 5/BSO(right).jpg]

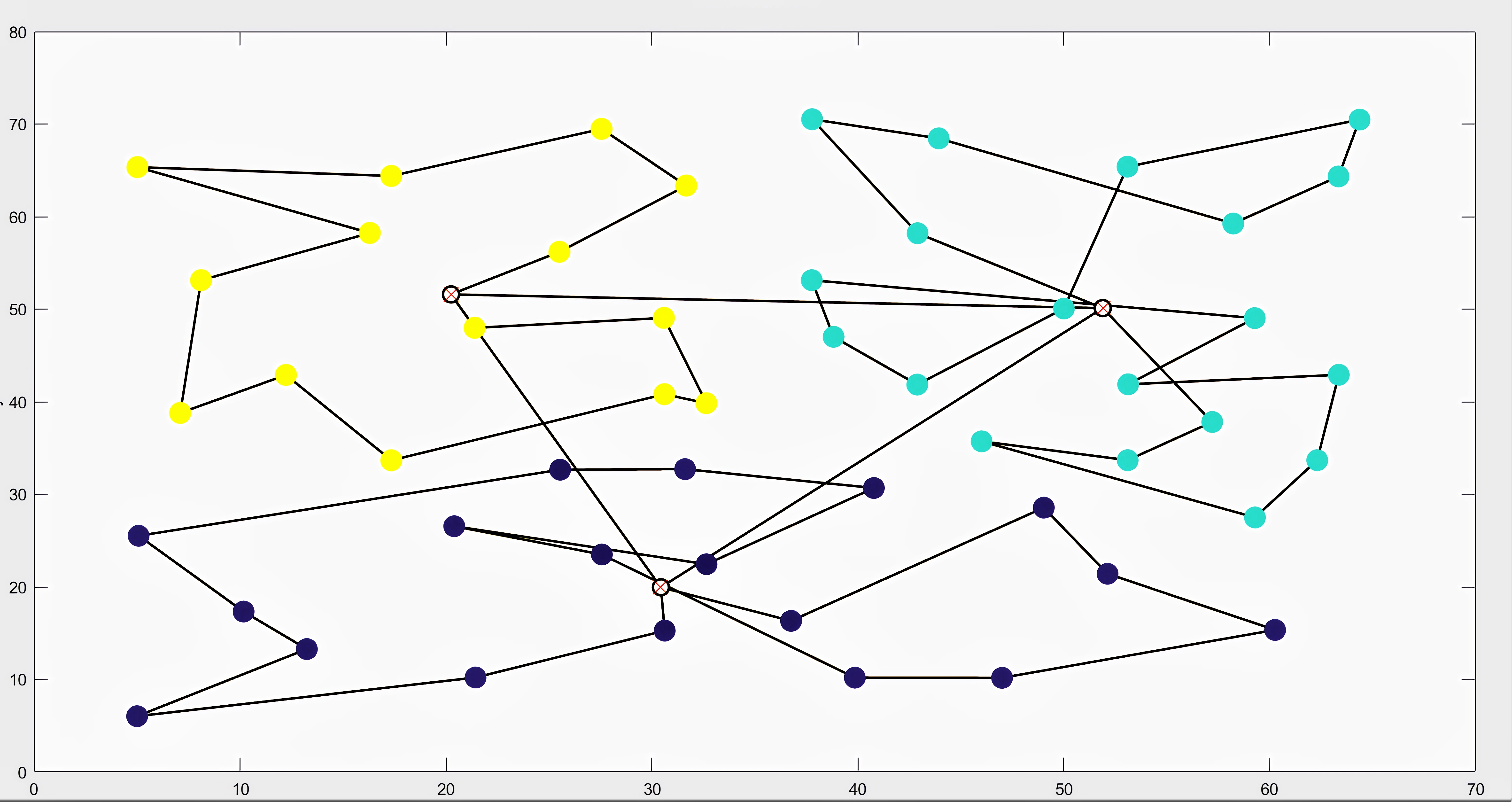

Supplement: Supplementary file 2 [file DataSheet2.zip › Table 5/GA(left).jpg]

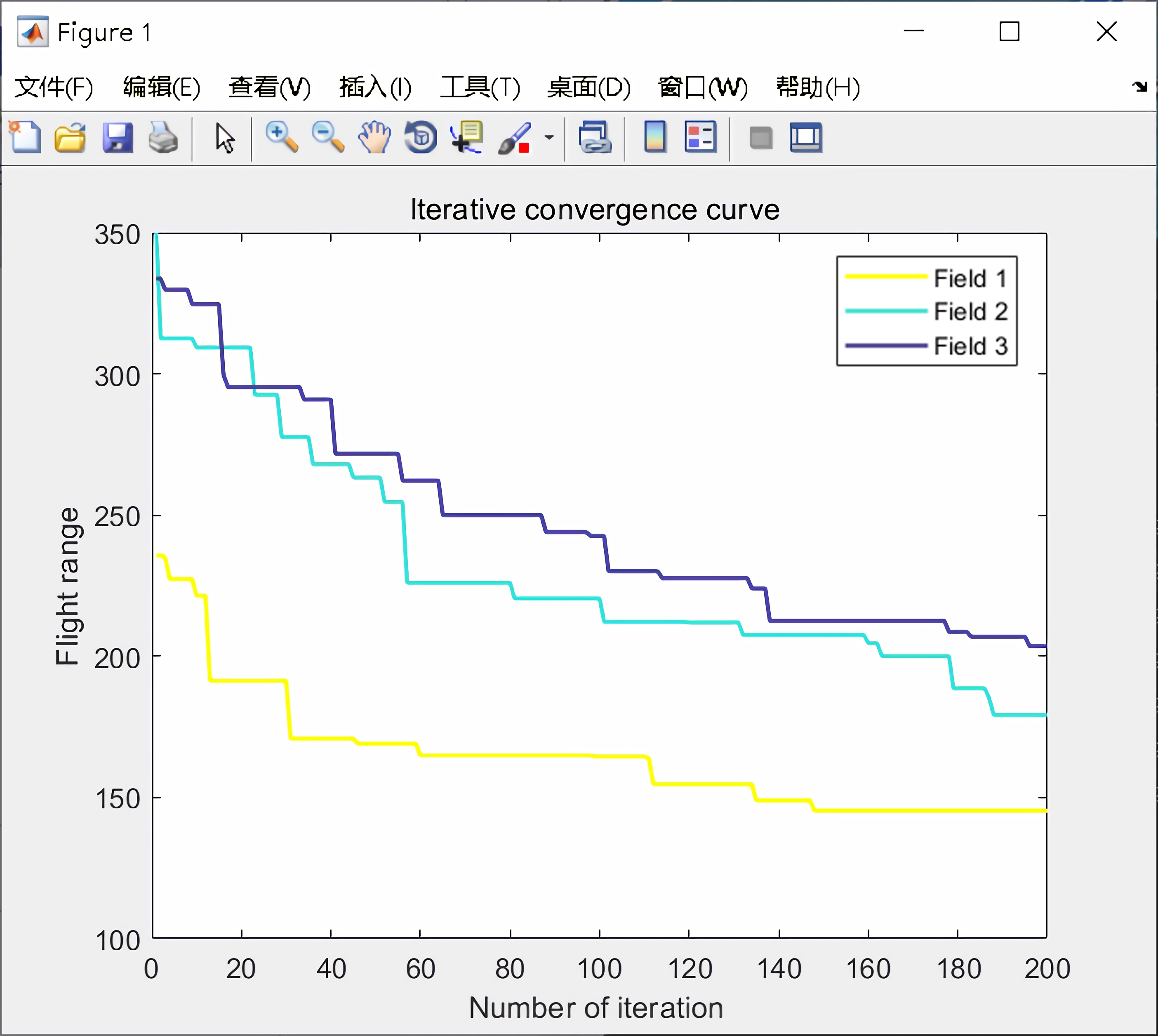

Supplement: Supplementary file 2 [file DataSheet2.zip › Table 5/GA(right).jpg]

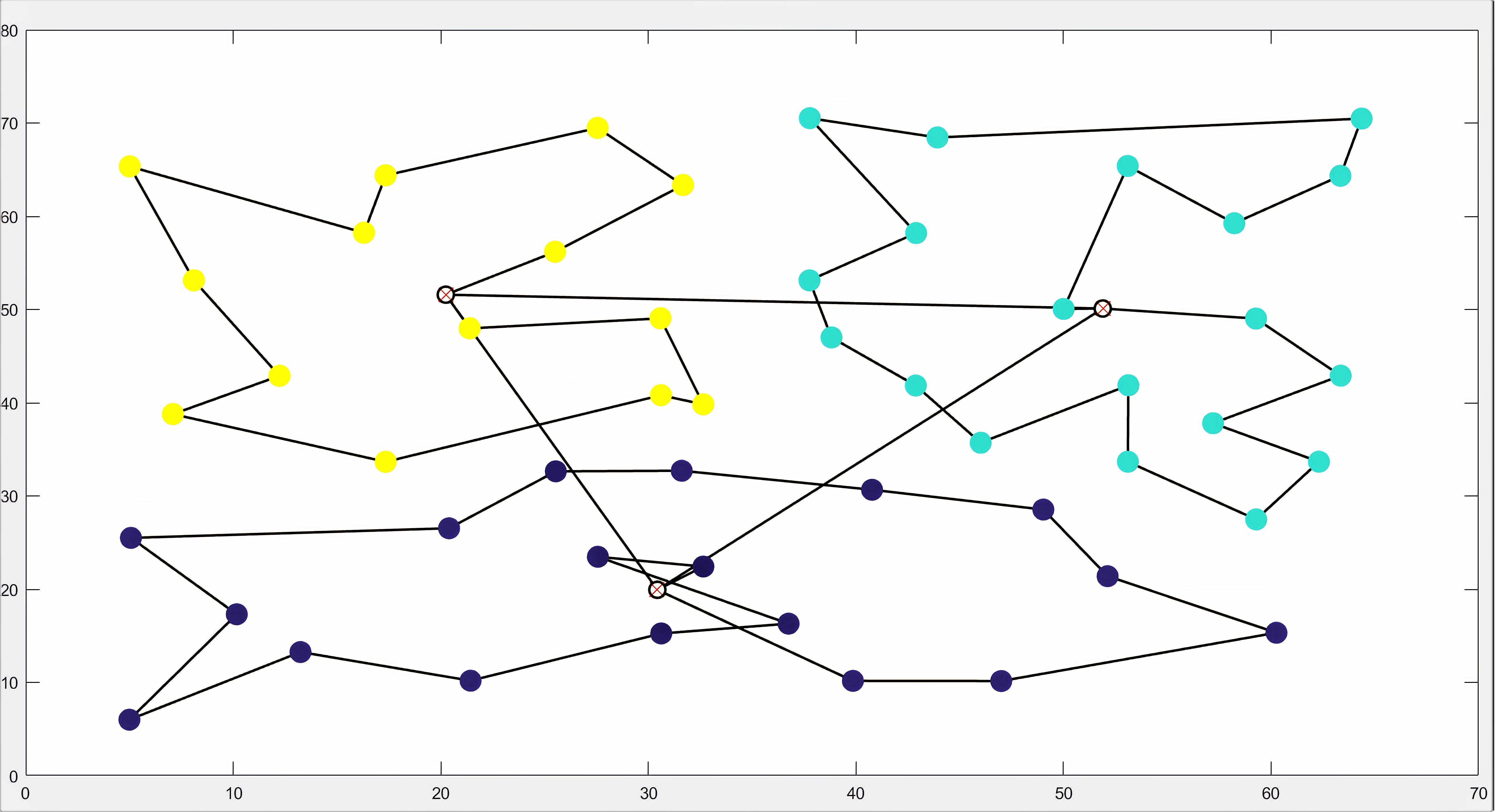

Supplement: Supplementary file 2 [file DataSheet2.zip › Table 5/GA-ACO(left).jpg]

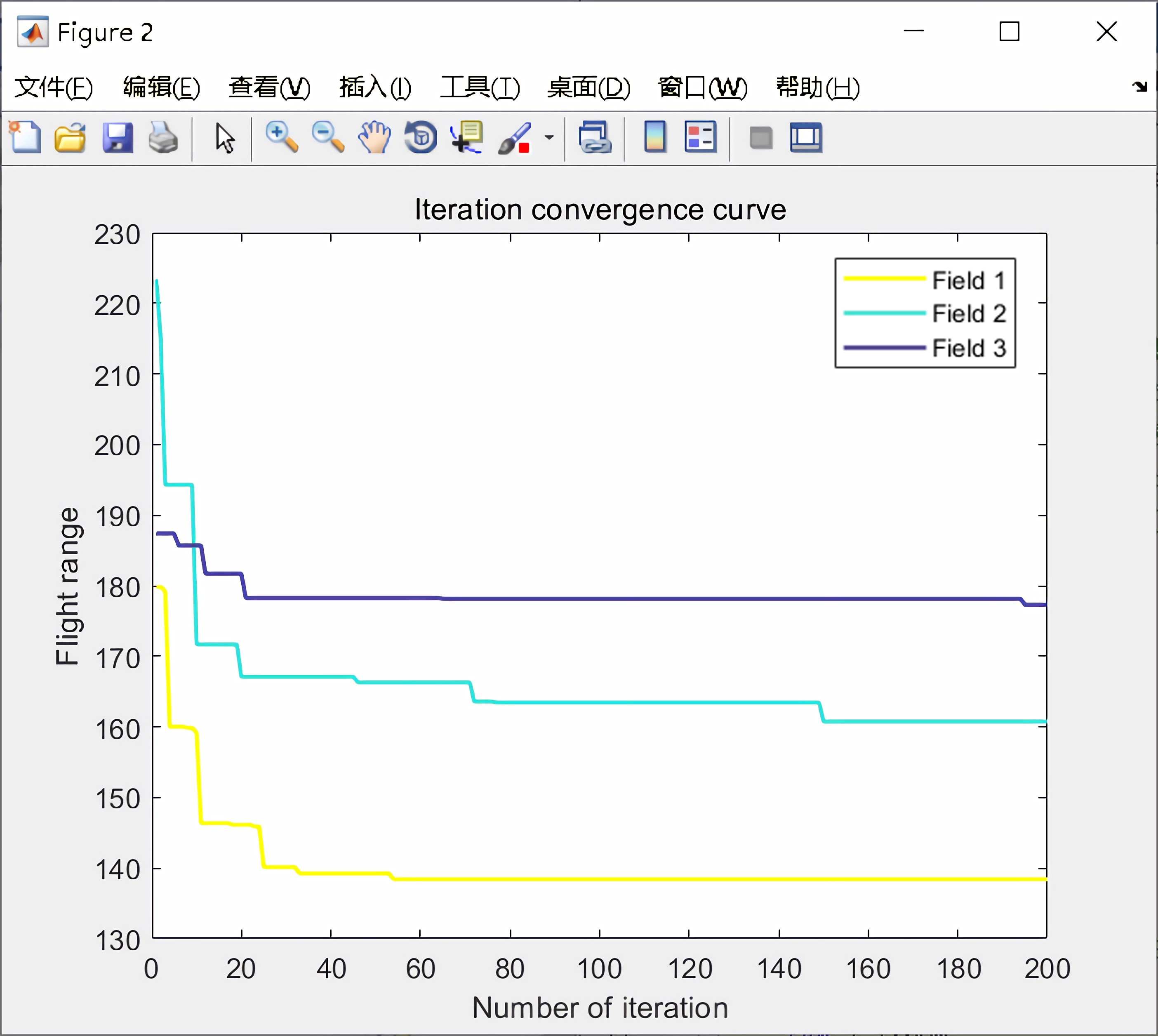

Supplement: Supplementary file 2 [file DataSheet2.zip › Table 5/GA-ACO(right).jpg]

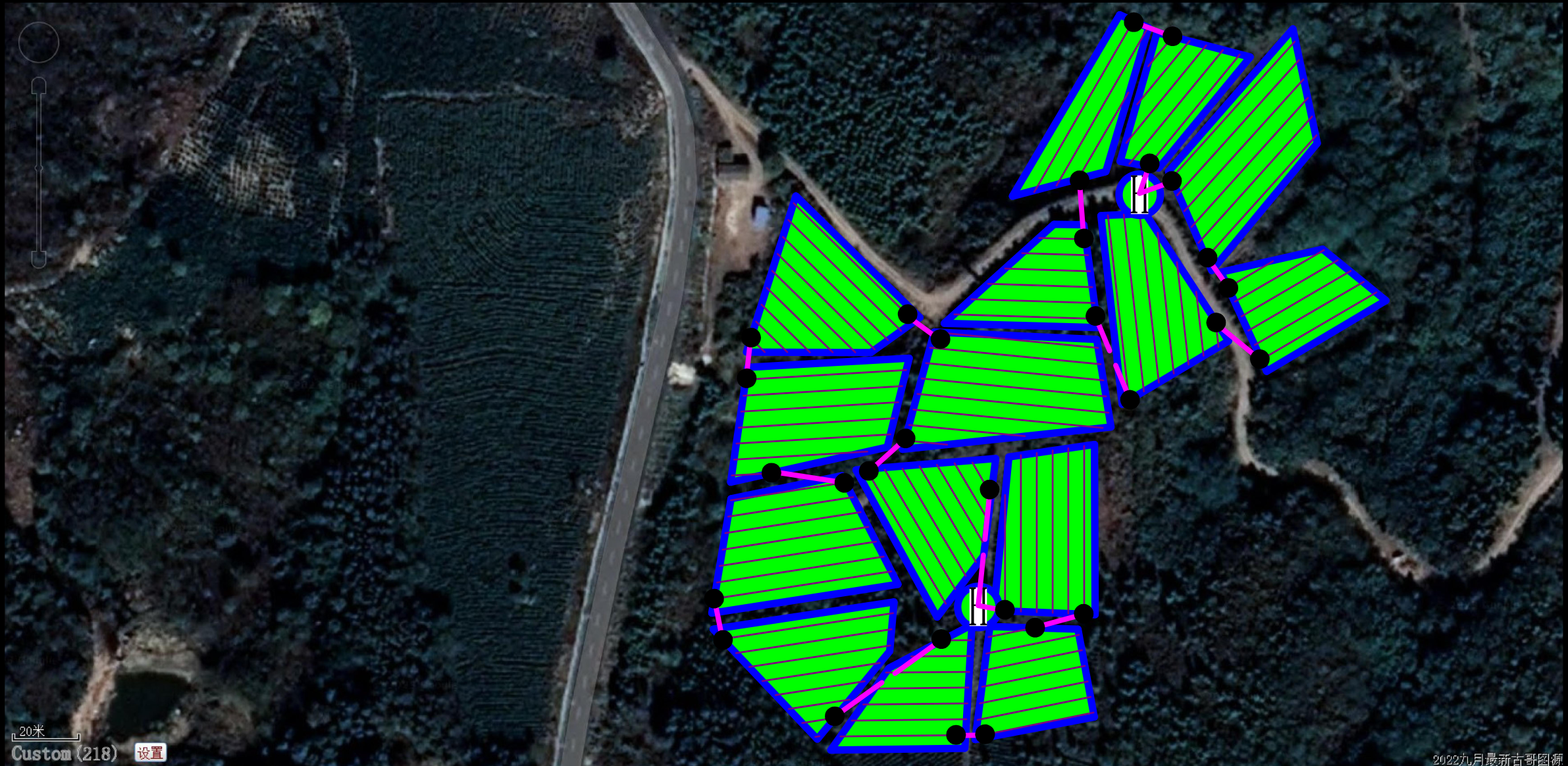

Supplement: Supplementary file 3 [file DataSheet3.zip › Table 8/ACHAGA(left).jpg]

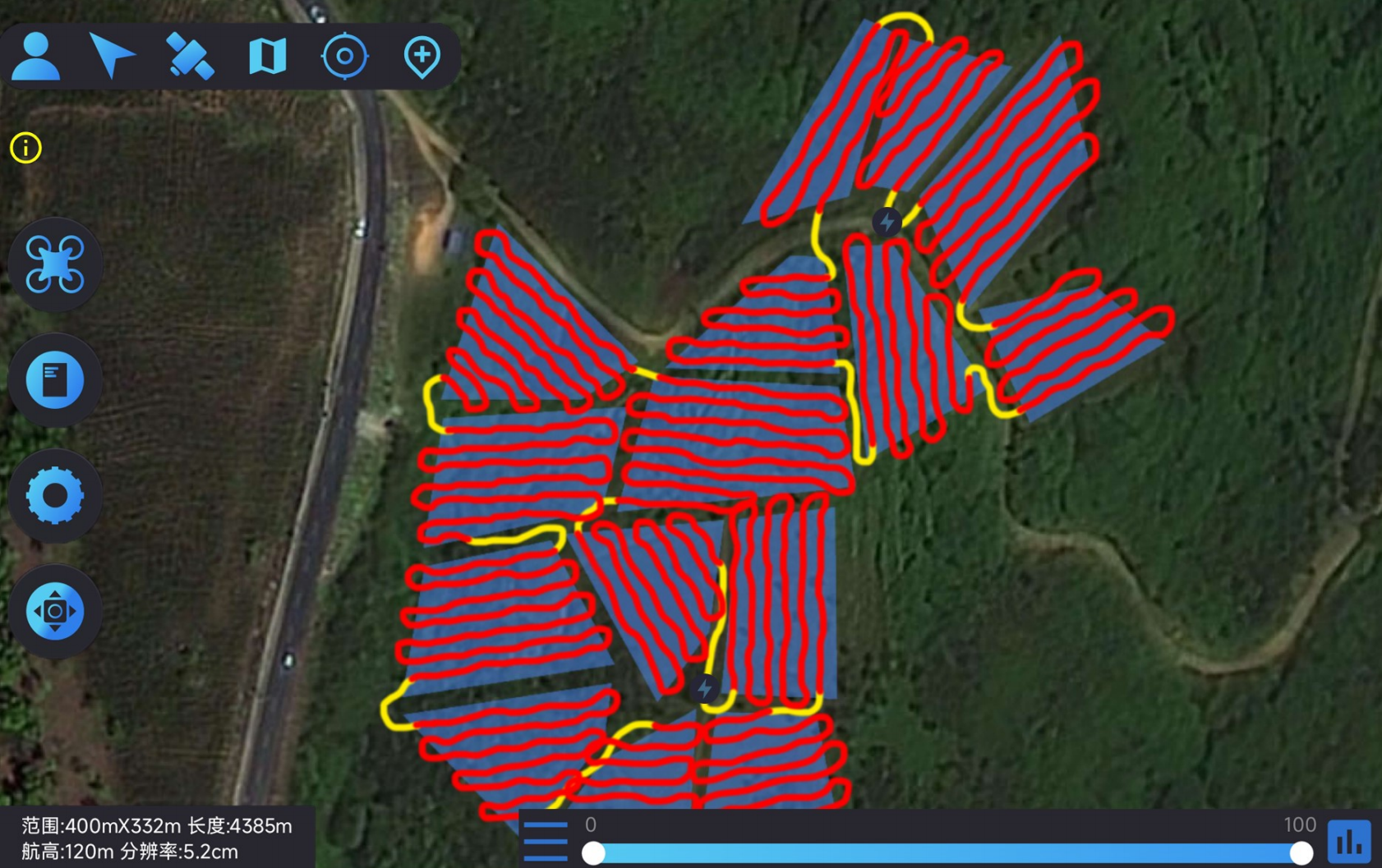

Supplement: Supplementary file 3 [file DataSheet3.zip › Table 8/ACHAGA(right).jpg]

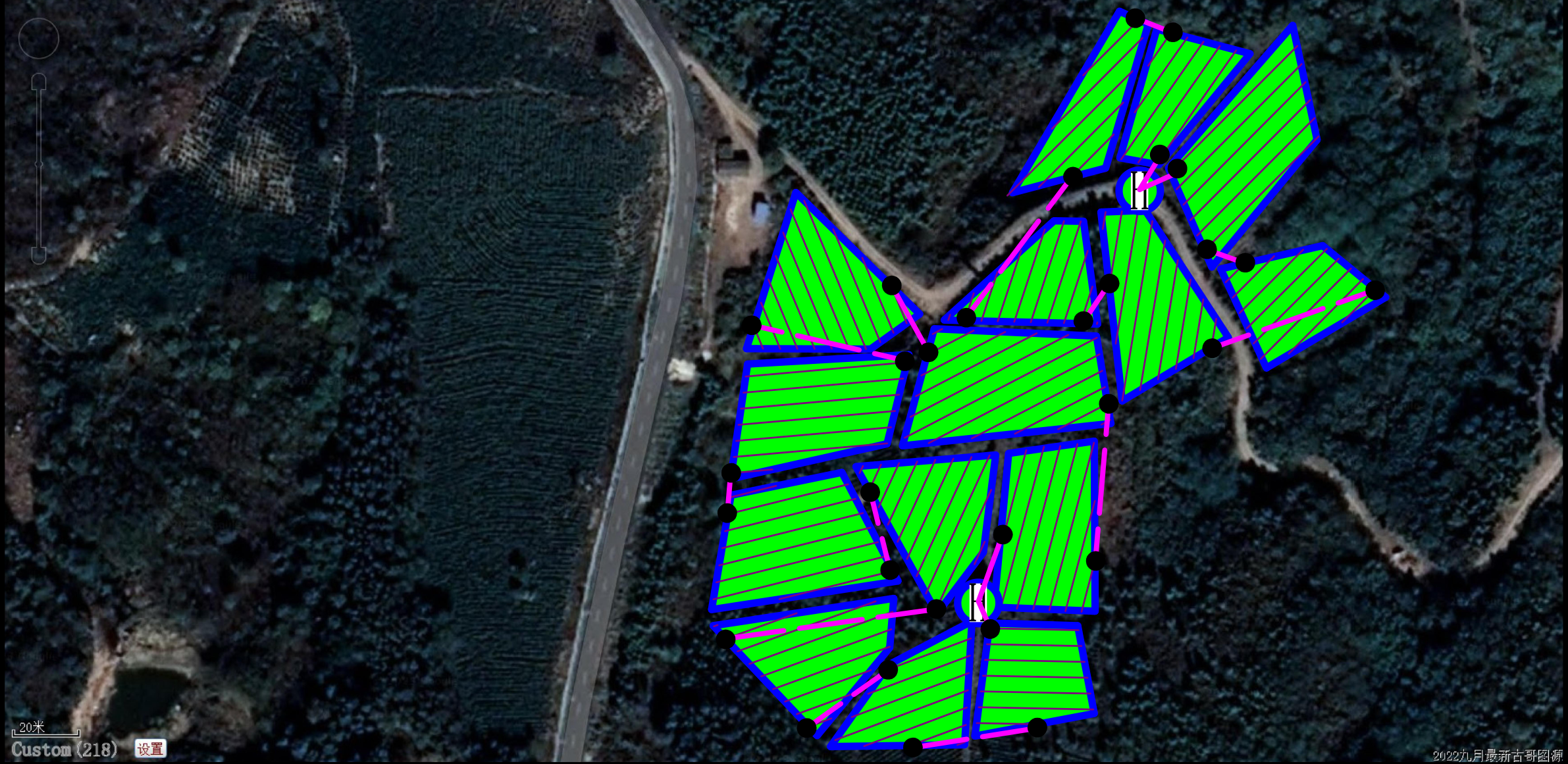

Supplement: Supplementary file 3 [file DataSheet3.zip › Table 8/AE(left).jpg]

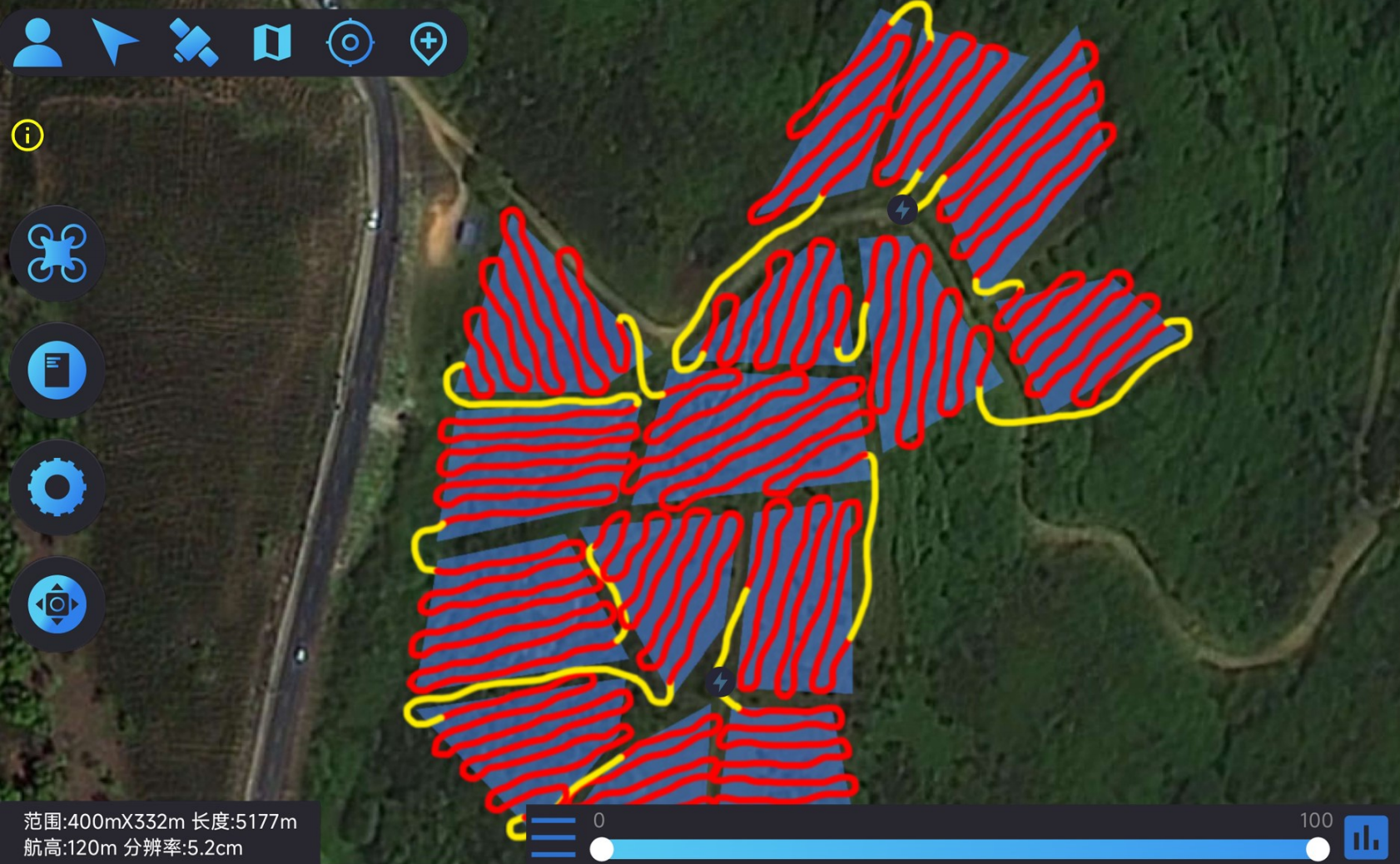

Supplement: Supplementary file 3 [file DataSheet3.zip › Table 8/AE(right).jpg]

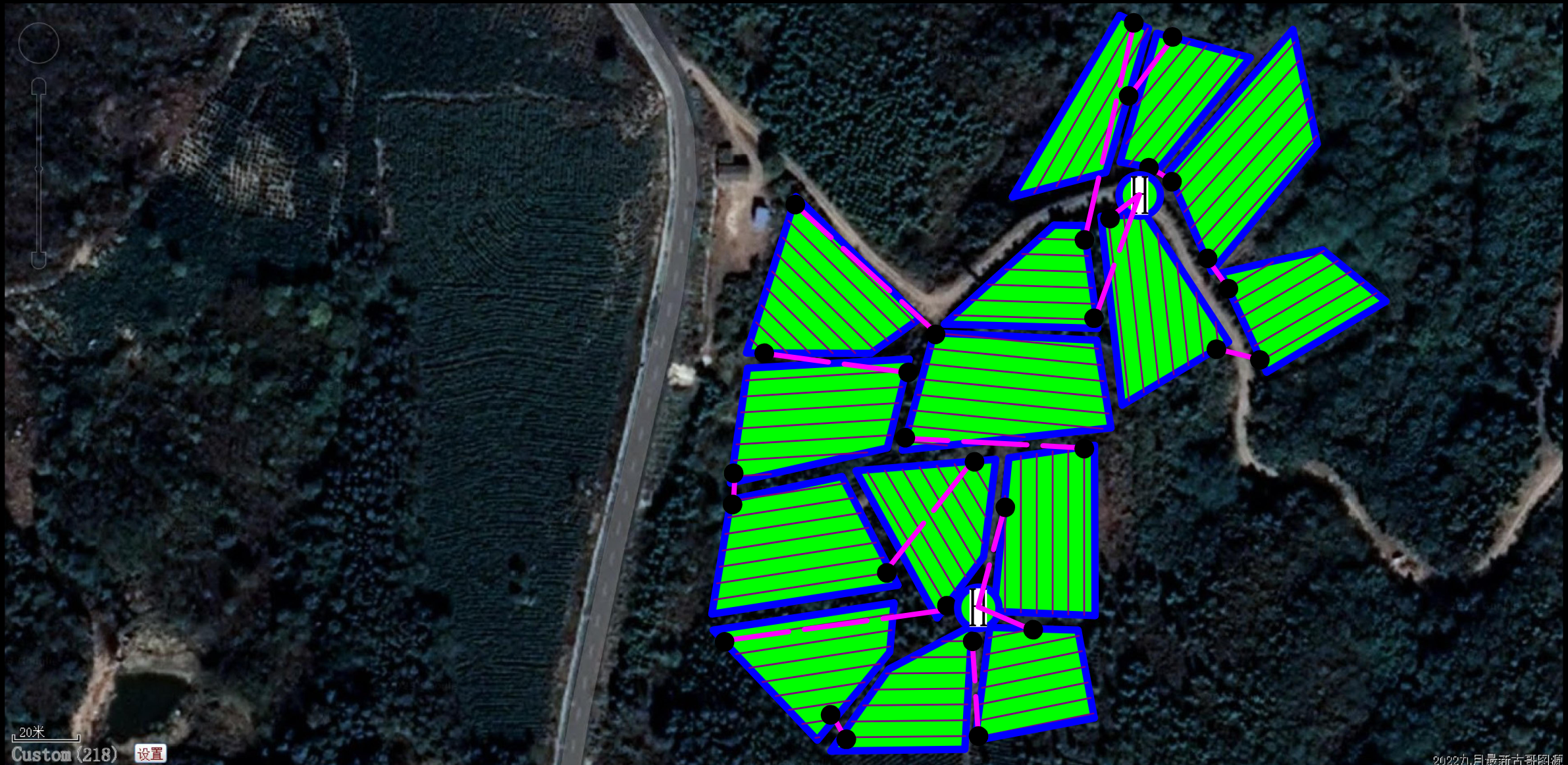

Supplement: Supplementary file 3 [file DataSheet3.zip › Table 8/BSO(left).jpg]

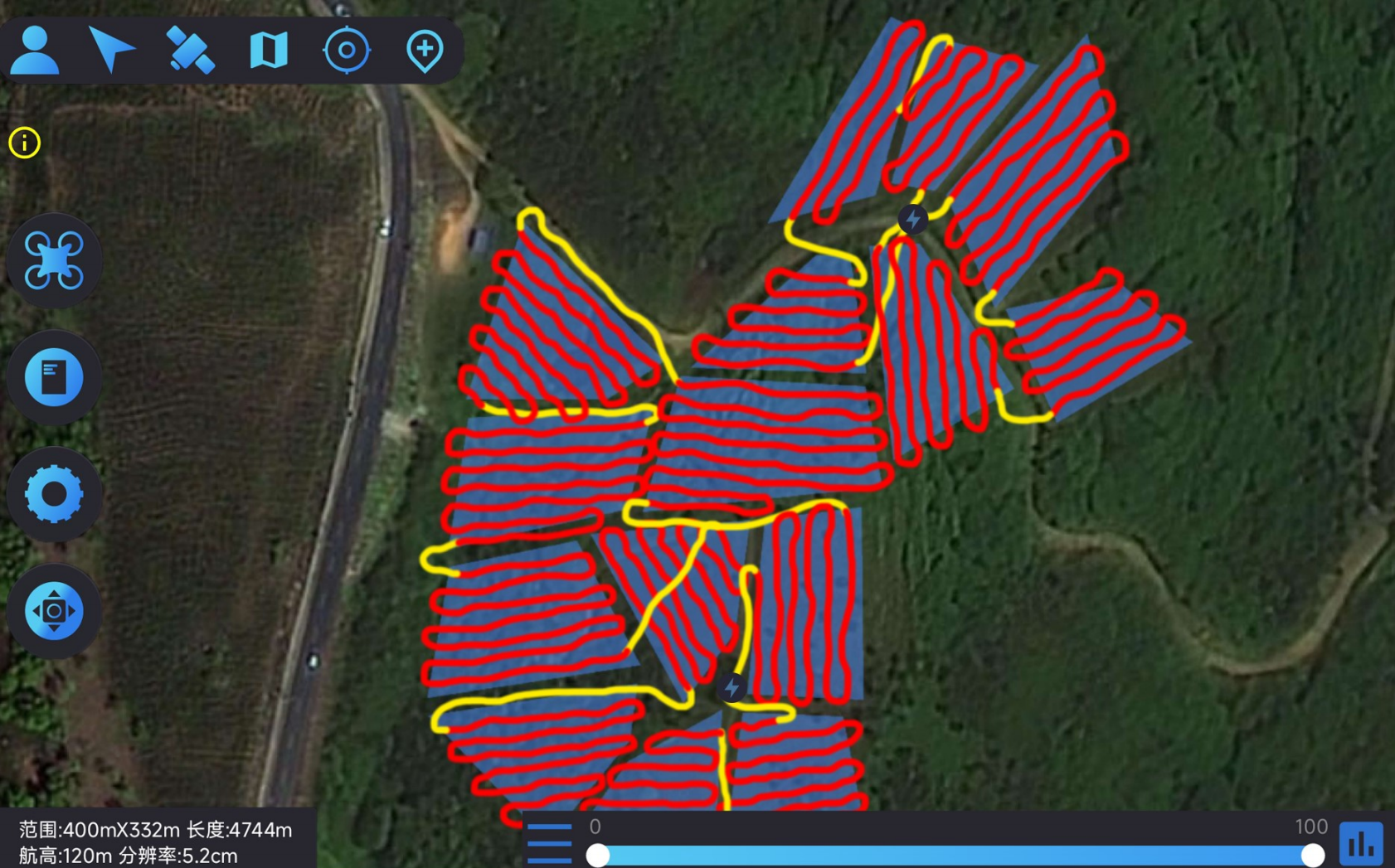

Supplement: Supplementary file 3 [file DataSheet3.zip › Table 8/BSO(right).jpg]

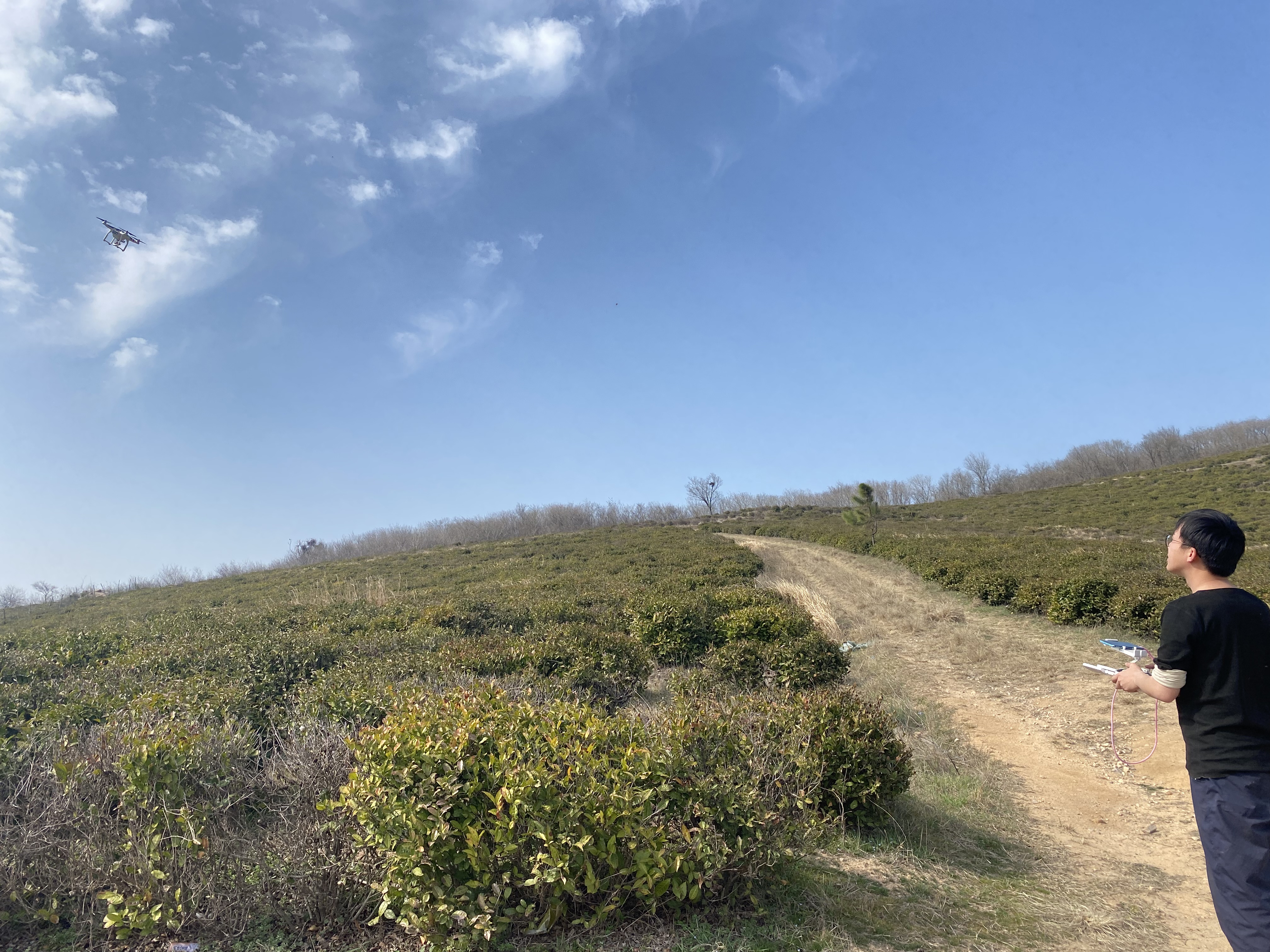

Supplement: Supplementary file 4 [file DataSheet4.zip › Supplementary images1/Fig. 11(a).jpg]

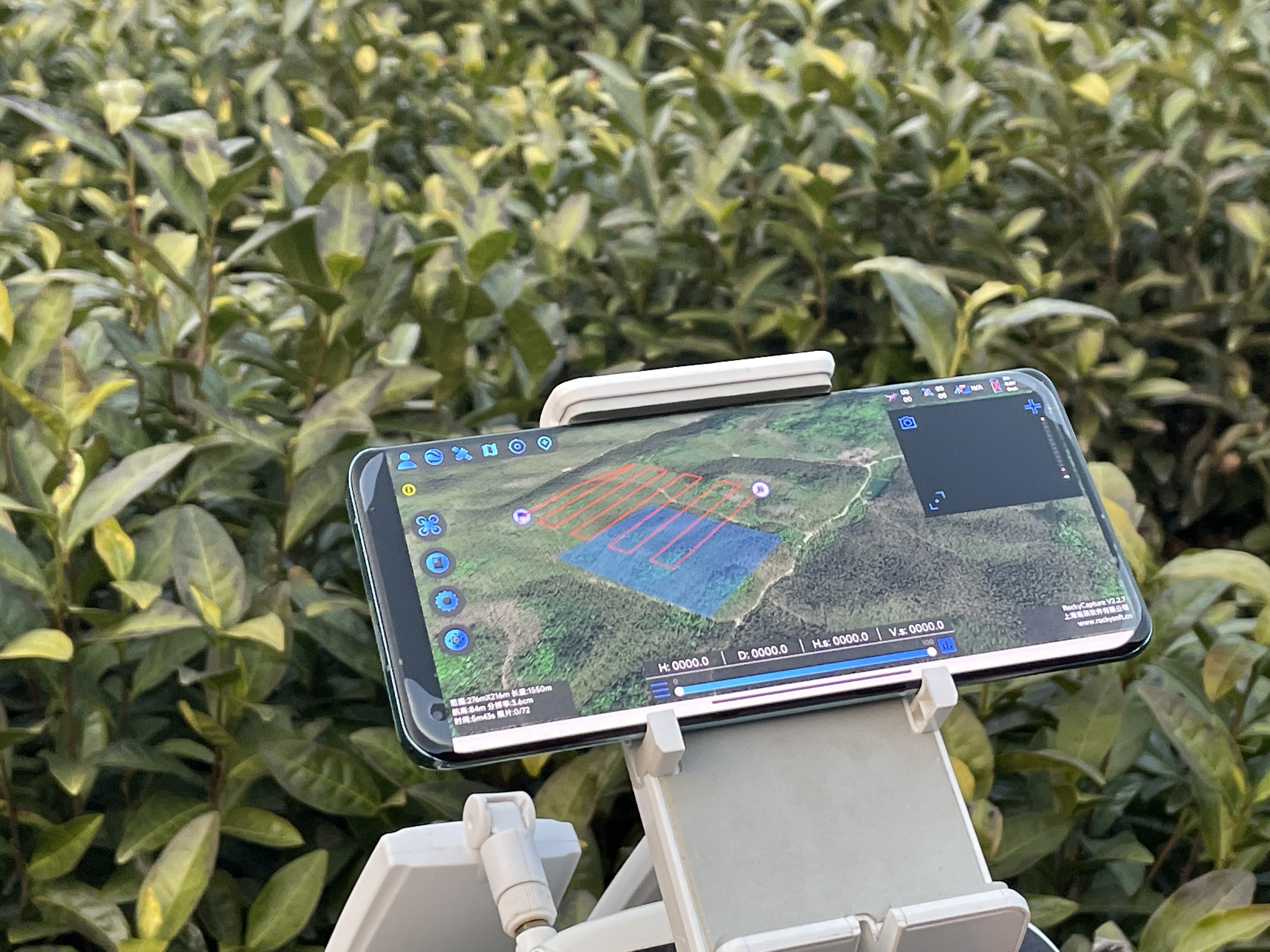

Supplement: Supplementary file 4 [file DataSheet4.zip › Supplementary images1/Fig. 11(b).jpg]

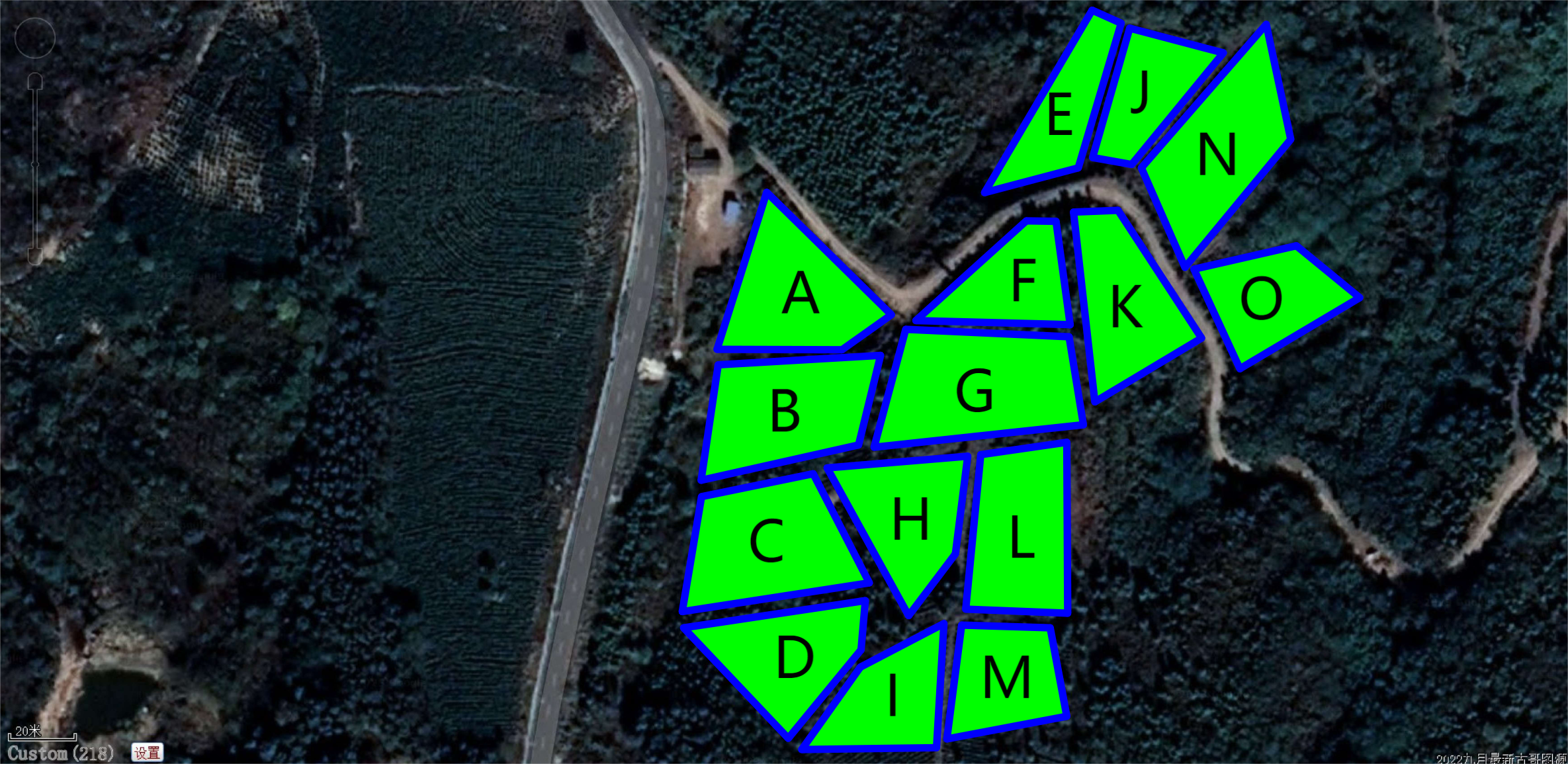

Supplement: Supplementary file 4 [file DataSheet4.zip › Supplementary images1/Fig. 12.jpg]

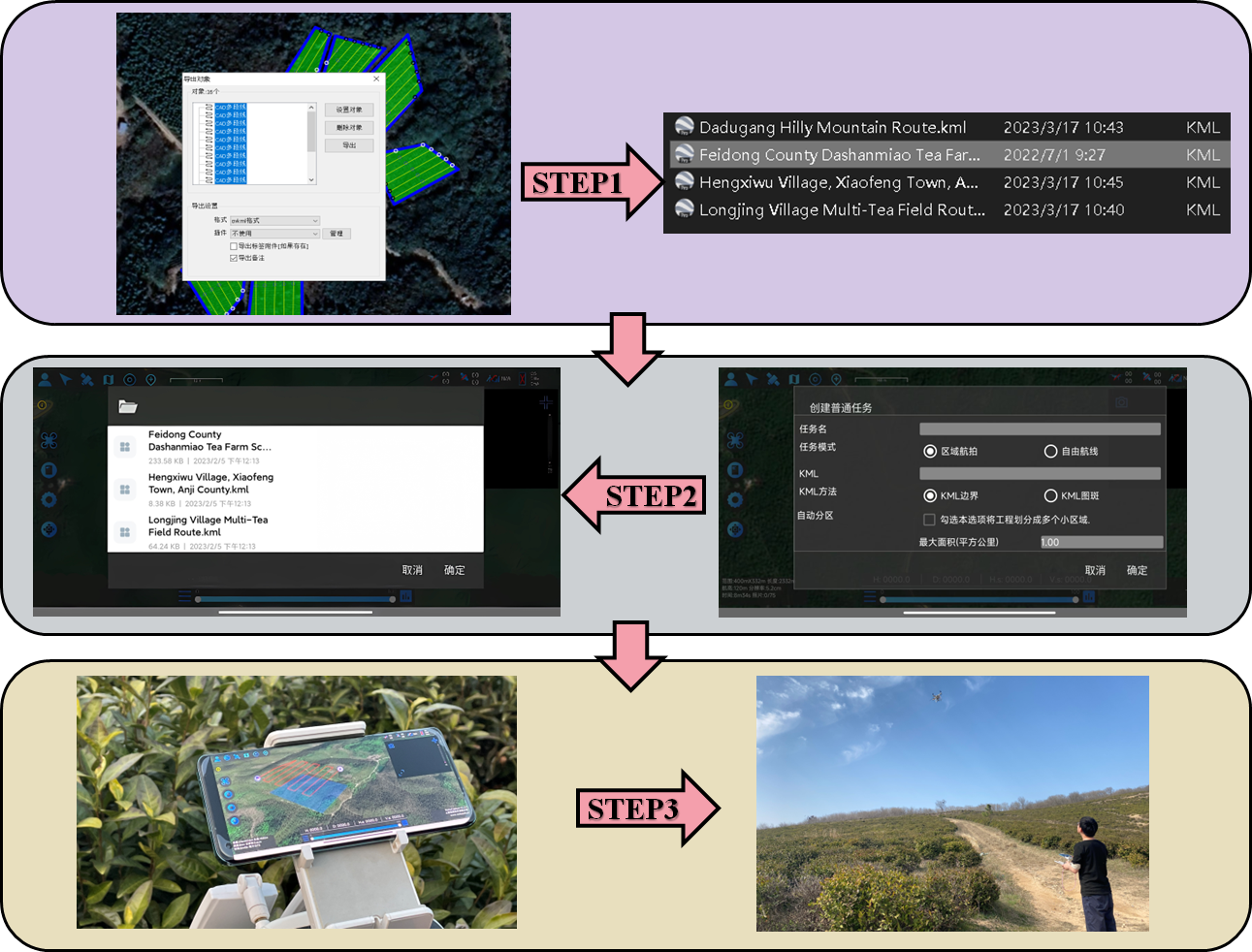

Supplement: Supplementary file 4 [file DataSheet4.zip › Supplementary images1/Fig. 13.jpg]

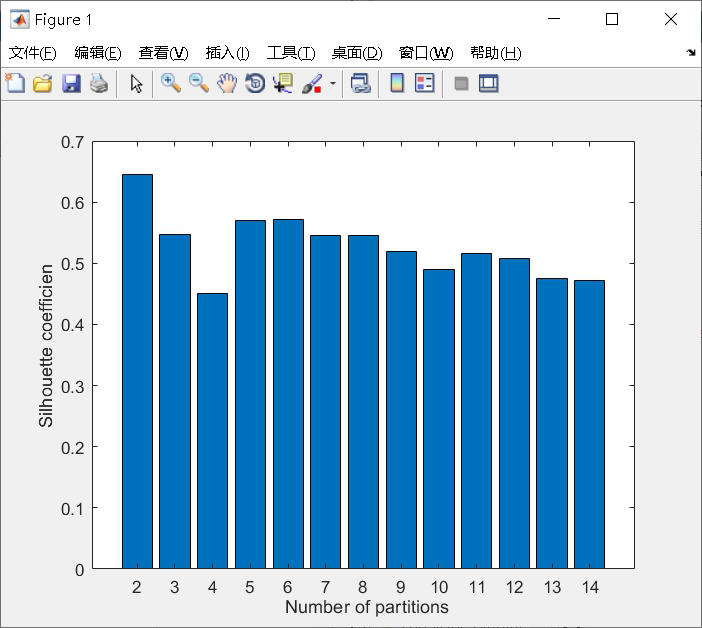

Supplement: Supplementary file 4 [file DataSheet4.zip › Supplementary images1/Fig. 14(a).jpg]

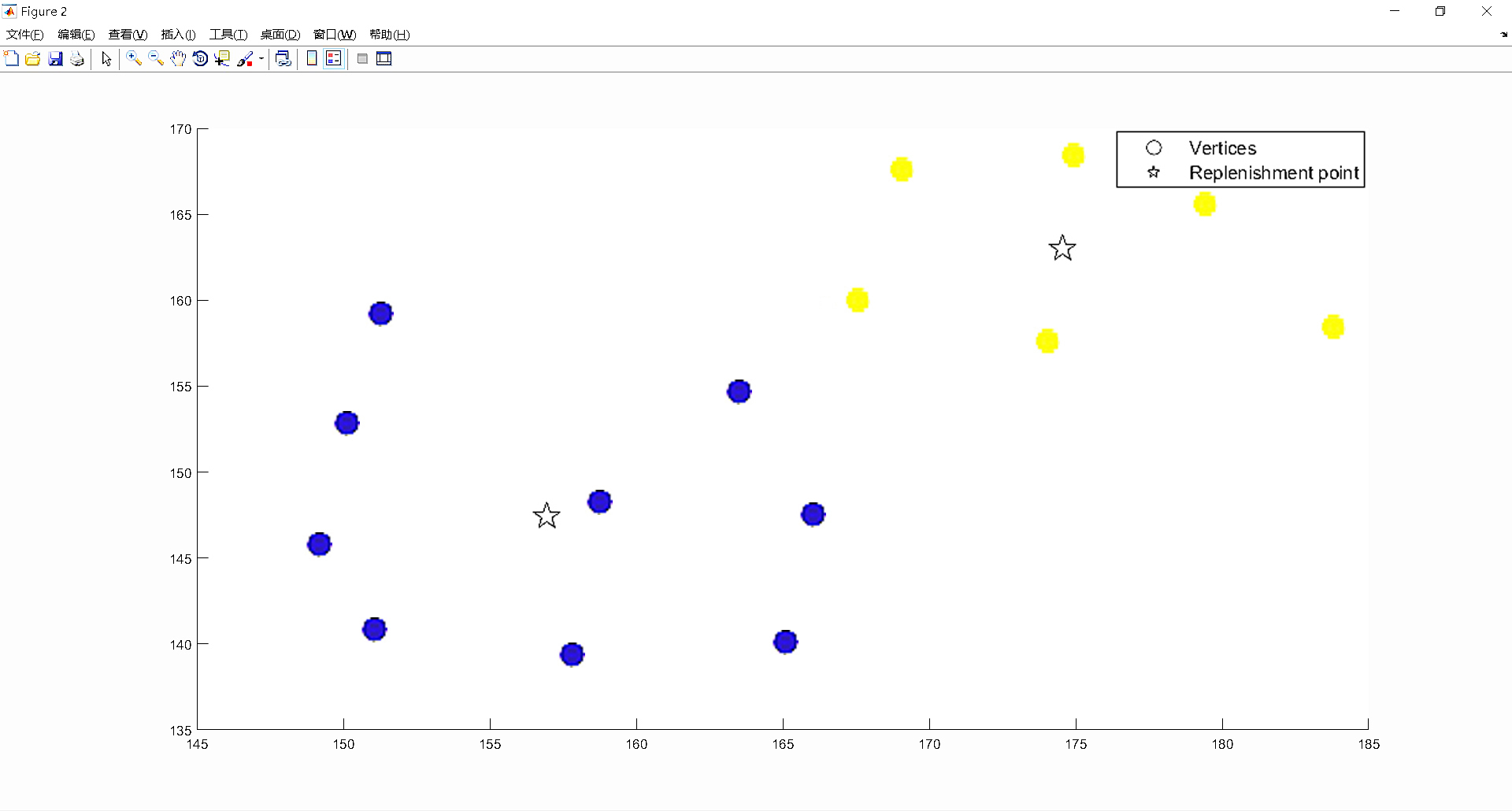

Supplement: Supplementary file 4 [file DataSheet4.zip › Supplementary images1/Fig. 14(b).jpg]

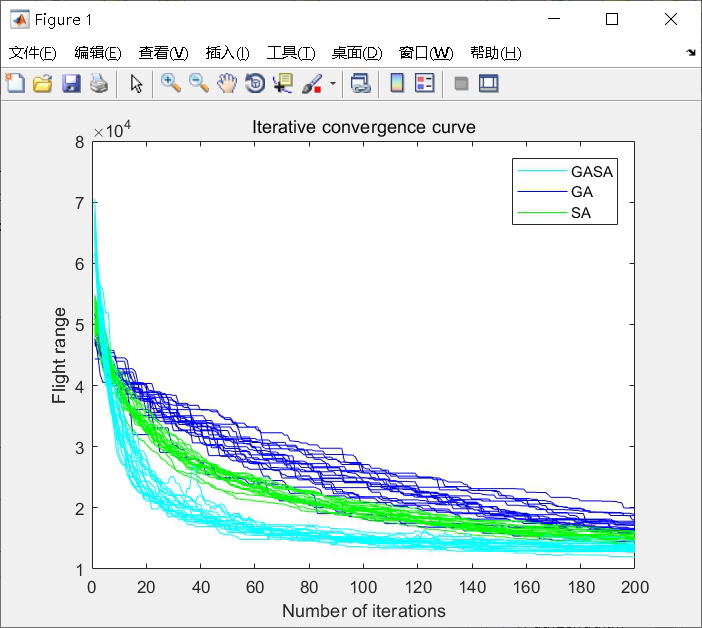

Supplement: Supplementary file 4 [file DataSheet4.zip › Supplementary images1/Fig. 15(a).jpg]

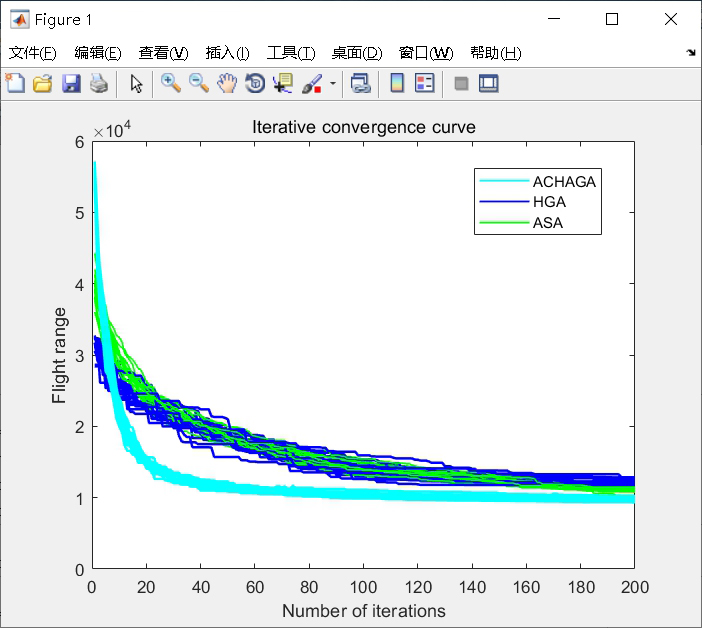

Supplement: Supplementary file 4 [file DataSheet4.zip › Supplementary images1/Fig. 15(b).jpg]

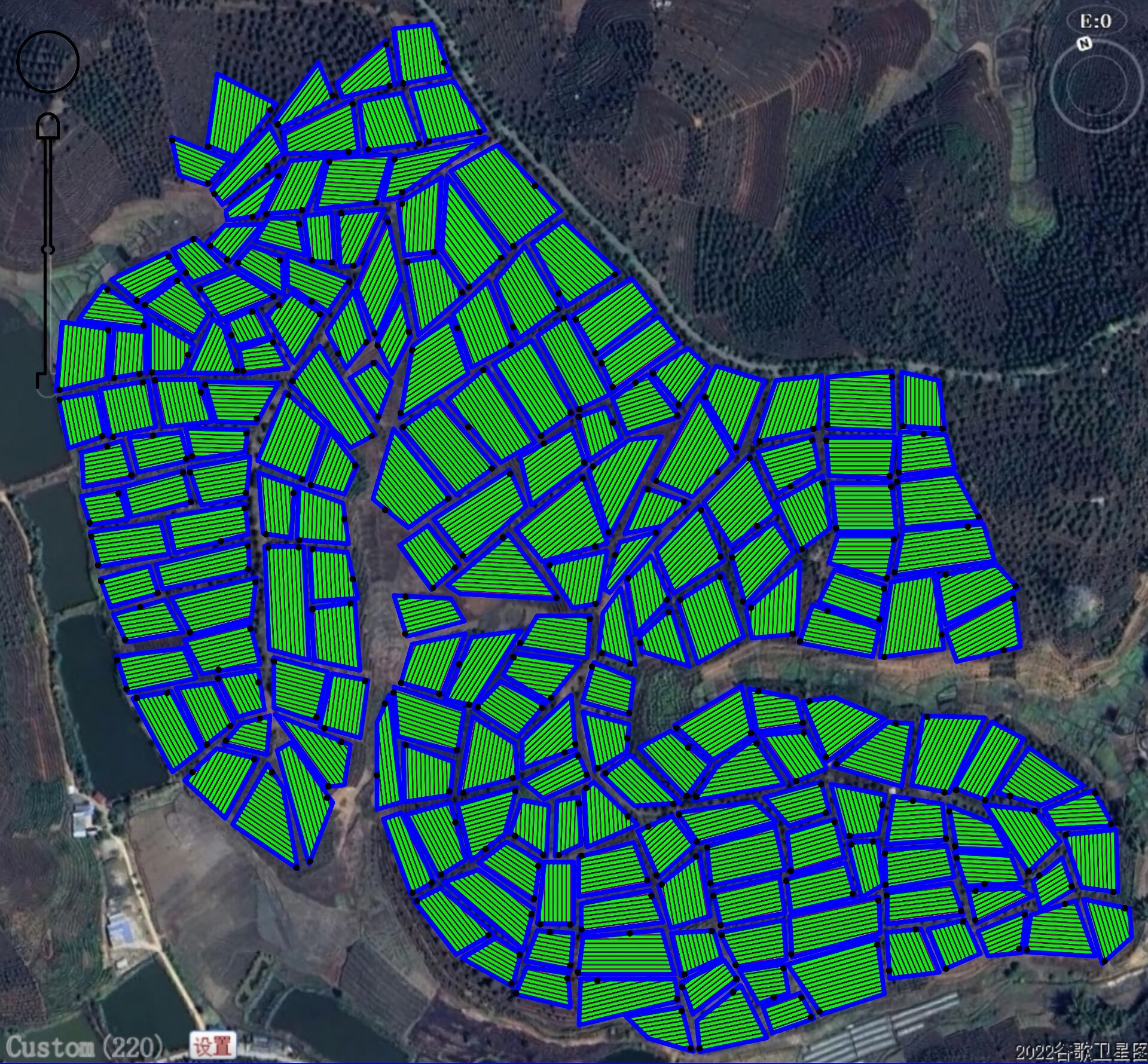

Supplement: Supplementary file 4 [file DataSheet4.zip › Supplementary images1/Fig.7b.jpg]
