# Supplementary material for: An integrated framework for UAV-based precision plant protection in complex terrain: the ACHAGA solution for multi-tea fields
Source: Front Plant Sci. 2024 Sep 26;15:1440234. doi: 10.3389/fpls.2024.1440234 (PMC11464358; doi:10.3389/fpls.2024.1440234)
Supplement: Supplementary file 6 [file Table2.docx]

**Appendix A.** Pseudo-code for a replenishment location planning algorithm

| **Algorithm 1: Replenishment Location Planning Algorithm** |
| --- |
| **Input:**  C: Cluster assignments for each data point in X  μ: Coordinates of the cluster centroids  tank_capacity: Capacity of the chemical tank in the UAV  efficiency: Efficiency of the UAV's plant protection  **Output:**  resupply_points: Coordinates of the resupply center points for each cluster  01: resupply_points ← ∅  02: **for** j = 1 . . . k  03: resupply_points(j,:) ← planResupply(Cj, tank_capacity, efficiency)  04: **end for**  Function: planResupply(Xj, tank_capacity, efficiency)  01: centroid ← mean(Xj)  02: total_distance ← 0  03: **for** i = 1 . . . \|Xj\|  04: total_distance ← total_distance + distance(Xj(i), centroid)  05: **end for**  06: total_time ← total_distance / efficiency  07: resupply_point ← centroid + (total_time * efficiency * tank_capacity)  08: return resupply_point |

**Appendix B.** Pseudo-code for algorithms of assigning flight sorties for UAV operations

| Algorithm 2: UAV Operations Sortie Allocation Algorithm |
| --- |
| **Input:**C: Cluster assignments for each data point in X  resupply_points：Coordinates of the resupply center points for each cluster  tank_capacity：Capacity of the chemical tank in the UAV  efficiency：Efficiency of the UAV's plant protection  **Output:**  flight_plan：A list of flight plans for each cluster  01：flight_plan ← []  02：**for** j = 1 . . . k  03：flight_plan.append(planFlight(Cj, resupply_points(j,:), tank_capacity, efficiency))  04：**end for**  Function: planFlight(Xj, S1, tank_capacity, efficiency)  01: flight_plan ← []  02: Sd ← tank_capacity * efficiency * 60 // single flight maximum area in minutes  03: Sm ← sum(Xj) // total area of the cluster  04: **if** Sm < Sd // case 1, no need to return to base  05: j ← 1 // number of flights is 1  06: payload ← Sm / Sd * tank_capacity // payload is proportional to the area ratio  07 **else** // case 2, need to return to base at least once  08 j ← int(Sm / Sd) + 1 // number of flights is the quotient plus one  09 payload ← tank_capacity // payload is full  10 **end if**  11 flight_plan.append(j) // add number of flights to the plan  12 flight_plan.append(payload) // add payload to the plan  13 route ← [] // initialize route list  14 centroid ← mean(Xj) // calculate cluster centroid  15 **for** i = 1 . . . j // loop over flights  16 start_point ← resupply_point // start from resupply point  17 end_point ← centroid + (i / j) * (resupply_point - centroid) // end at a point between centroid and resupply point  18 route.append([start_point, end_point]) // add start and end points to route  19 **end for**  20 flight_plan.append(route) // add route to the plan  21 return flight_plan |
